# Fluorescent 4‑Nitrobenzo-2-oxa-1,3-diazole-Coupled Bile Acids as Probe Substrates of Hepatic and Intestinal Bile Acid Transporters of the Solute Carrier Families SLC10 and SLCO

**DOI:** 10.1021/acs.jmedchem.5c00589

**Published:** 2025-05-17

**Authors:** Celine Drossel, Sebastian Kunz, Christopher Neelen, Mats Georg, Yohannes Hagos, Dieter Glebe, Richard Göttlich, Joachim Geyer

**Affiliations:** 1 Institute of Organic Chemistry, 9175 Justus Liebig University Giessen, Heinrich-Buff-Ring 17, Giessen 35392, Germany; 2 Institute of Pharmacology and Toxicology, 9175 Justus Liebig University Giessen, Schubertstr. 81, Giessen 35392, Germany; 3 PortaCellTec Biosciences GmbH, Science Park Va, Marie-Curie-Strasse 8, Göttingen 37079, Germany; 4 National Reference Centre for Hepatitis B viruses and Hepatitis D viruses, German Center for Infection Research (DZIF), Institute of Medical Virology, Justus Liebig University of Giessen, partner site Giessen-Marburg-Langen, Schubertstr. 81, Giessen 35392, Germany

## Abstract

Several bile acid (BA) transporters are involved in the enterohepatic BA circulation between the liver and gut, including the hepatic Na^+^/taurocholate cotransporting polypeptide (NTCP) and the intestinal apical sodium-dependent BA transporter (ASBT). Fluorescent BA derivatives are helpful to measure and visualize BA transport *in vitro* and *in vivo*. We used 4-nitrobenzo-2-oxa-1,3-diazole (NBD) as the labeling fluorophore and synthesized a series of 3-NBD-coupled BA. While 3α-NBD-taurocholic acid, 3β-NBD-taurocholic acid, 3α-NBD-glycocholic acid, and 3β-NBD-glycocholic acid showed significant transport rates for human NTCP, mouse mNtcp, and mouse mAsbt, human ASBT only showed reliable transport activity for 3α-NBD-glycocholic acid. In general, NBD coupling to the 3α-position proved superior to the 3β-position, and the NBD-BA with glycine conjugation exhibited the highest overall transport rates. None of the synthesized NBD-BA was transported by the organic anion transporting polypeptides OATP1B1 and OATP1B3. Overall, 3α-NBD-glycocholic acid is most appropriate for fluorescence-based transport assays to evaluate NTCP and ASBT inhibitors.

## Graphical abstract

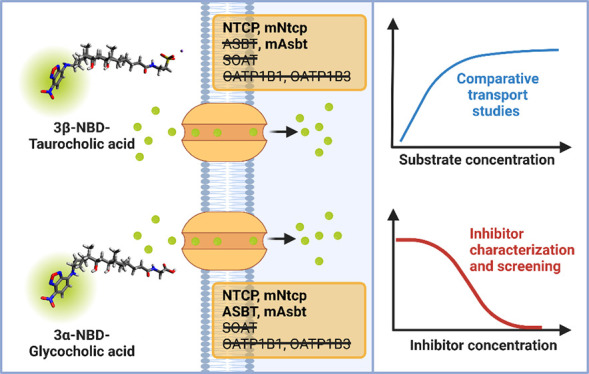

## Introduction

Bile acids (BA) are synthesized in the liver from cholesterol and circulate between the liver and the intestine, a process called enterohepatic circulation (EHC).^1,2^ Several BA transporters in the liver and the gut are involved in this process.^3^ After their synthesis, BA are largely conjugated with taurine (T) or glycine (G), and form a pool of primary conjugated BA such as taurocholic acid (TCA) or glycocholic acid (GCA).^4^ As these conjugated BA are mostly deprotonated at physiological pH, they are commonly referred to as bile salts (BS).^5^ After their synthesis and conjugation, BS are excreted from hepatocytes into the bile canaliculi mainly by the bile salt efflux pump BSEP, an ATP-driven efflux transporter from the ATP-binding cassette transporter family (gene symbol *ABCB11*).^6^ With the bile flow, BS reach the intestinal lumen, where they are important for the absorption of dietary lipids and fat-soluble vitamins.^4^ At the terminal ileum, more than 90% of conjugated BS are actively reabsorbed via the apical sodium-dependent bile acid transporter ASBT (gene symbol *SLC10A2*)^7^ that is localized at the apical brush border membrane of ileal enterocytes.^8^ By bypassing this absorption, BS enter the colon, where they are partially deconjugated and modified by the gut microbiome, ultimately resulting in a pool of unconjugated secondary BA that can be passively absorbed along the colon.^1^ Primary and secondary BS and BA then are transported back to the liver, where they are taken up from the portal blood by two different transport systems.^9^ The Na^+^/taurocholate cotransporting polypeptide NTCP (gene symbol *SLC10A1*) is localized at the basolateral membrane of hepatocytes^10^ and preferentially transports conjugated BS by secondary active Na^+^-dependent transport.^11^ In addition, three members of the organic anion transporting polypeptide transporter (OATP) family, namely OATP1B1, OATP1B3, and OATP2B1 are involved in the hepatic BA uptake.^5,12^ OATPs mediate sodium-independent transport and prefer unconjugated over conjugated BA.^13–15^ Within hepatocytes, unconjugated primary and secondary BA are reconjugated to BS and then are excreted into bile.^1^ Apart from its role as BA carrier, NTCP has been identified as high-affinity hepatic entry receptor for the hepatitis B and D viruses (HBV/HDV).^16^

Besides its intestinal expression, ASBT is also localized in the apical domain of cholangiocytes that line the bile ducts in the liver.^17^ Here, ASBT is involved in the process of so-called chole-hepatic shunting of BS that plays a regulatory role for the hepatic bile flow.^18,19^ In the kidney, ASBT is localized in the apical membrane domain of proximal tubule cells, where it mediates the reabsorption of BS that are filtered through the glomeruli causing a minimal loss of BS with the urine.^4,20^ However, under pathological cholestatic conditions, plasma BS concentrations significantly rise so that BS appear in larger amounts in the proximal tubule. As renal ASBT is downregulated under cholestatic conditions,^21,22^ BS can then be excreted with the urine as an alternative excretion route.^23^

As illustrated, membrane carriers are essential for the maintenance of the EHC of BS.^9^ This circulation can be disturbed or interrupted by genetic polymorphisms in one of these carriers or by drugs blocking the carrier-mediated BS transport, e.g. via BSEP.^3,24^ Furthermore, different types of liver diseases, such as cholestasis, hamper carrier-mediated hepatobiliary excretion of BS.^18,25^ On the other hand, dynamic liver function tests in patients make use of the active carrier-mediated hepatobiliary clearance of fluorescent dyes (e.g., indocyanine green), positron emission tomography (PET) probes (e.g., ^99m^Tc-mebrofenin), or magnetic resonance imaging (MRI) probes (e.g., Gd-EOB-DTPA) as diagnostic parameters.^5^ *In vitro*, BA transport can be investigated in cell culture models expressing one or more of the respective hepatic BA carriers. Most of these studies use radiolabeled BA as probe substrates, such as [^3^H]­taurocholic acid.^11,26,27^ In other publications, fluorescent BA such as cholyl-glycyl-amido-fluorescein (CGamF) or cholyl-l-lysyl-fluorescein (CLF) were used.^28–32^ Fluorescent BA as probe substrates for NTCP and ASBT are of particular interest for establishing high-throughput drug screening assays for the development of novel BA reabsorption inhibitors (BARIs), acting via ASBT inhibition, or HBV/HDV virus entry inhibitors, acting via NTCP inhibition. ASBT inhibitors such as elobixibat, linerixibat, maralixibat, and odevixibat are used to treat cholestatic disorders such as primary biliary cholangitis, intrahepatic cholestasis of pregnancy, Alagille syndrome or primary familial intrahepatic cholestasis. Their mode of action is to diminish the BS reabsorption from the gut and to lower the hepatic BS load.^33–35^ Several NTCP inhibitors are under development that block the binding of HBV/HDV virus particles to NTCP and thereby act as virus entry inhibitors.^36–38^

In the present study, we used 4-nitrobenzo-2-oxa-1,3-diazole (NBD) as the labeling fluorophore and synthesized a series of 3-NBD-coupled BA, with the NBD-fluorophore attached to position 3 of the steroid nucleus. These included the primary unconjugated BA, cholic acid (CA) and chenodeoxycholic acid (CDCA), the unconjugated secondary BA, deoxycholic acid (DCA), as well as the conjugated primary BS, TCA, GCA, and TCDCA. All these compounds showed stable and intensive fluorescence in cell culture models and were evaluated systematically as substrates of the BS carriers NTCP, ASBT, OATP1B1, and OATP1B3. The NTCP and ASBT homologue sodium-dependent organic anion transporter (SOAT, gene symbol *SLC10A6*) that is known to not transport BA served as a reasonable negative control. Apart from the human NTCP and ASBT carriers, also the mouse orthologs, namely mouse Ntcp (mNtcp) and mouse Asbt (mAsbt), were included in the functional analysis to identify potential species differences in the transport of the 3-NBD-BA derivatives. In addition to carrier screening, the present study investigated whether the orientation of the fluorophore label at the 3α- or 3β-position affects substrate recognition by the respective BS carrier.

## Results: Chemistry

The naturally occurring and commercially available BA cholic acid (CA), chenodeoxycholic acid (CDCA), and deoxycholic acid (DCA) represent the starting point for the synthesis of all 3-NBD-BA derivatives examined in the present study (see Scheme 1). Syntheses of 3-NBD-BA have been described in the literature before and these protocols served as inspiration for the following synthetic approach of the present study.^39,40^ As reported recently by our group for the synthesis of 3β-NBD-TCA,^41^ initial methyl esterification was accomplished utilizing thionyl chloride in methanol (**1a**–**c**). The 3 hydroxy group was then selectively mesylated with methanesulfonyl chloride to introduce a leaving group retaining the stereochemistry (**2a**–**c**). Regioselectivity was hereby mainly achieved due to the orientation of the hydroxy groups. While the axial 7 and 12 hydroxy groups encounter 1,3 diaxial interactions as well as greater steric hindrance, the equatorial 3 hydroxy group is more nucleophilic.^40,42,43^ Thereafter, azide formation was performed in S_N_2 fashion under stereoinversion. The resulting 3β-azido-BA (**3a**–**c**) were converted to the respective primary amines (**4a**–**c**) in a Staudinger reduction. Finally, the fluorophore was attached via S_N_Ar reaction using NBD chloride (**5a**–**c**). The substitution was hereby facilitated by the electron withdrawing properties of the substituents of the aromatic fluorophore.^44^ Saponification of the methyl ester gave the respective final 3β-NBD-BA, 3β-NBD-CA (**6a**), 3β-NBD-CDCA (**6b**), and 3β-NBD-DCA (**6c**) in six steps and overall yields of 17–34% (Scheme 1).

**Scheme 1:**
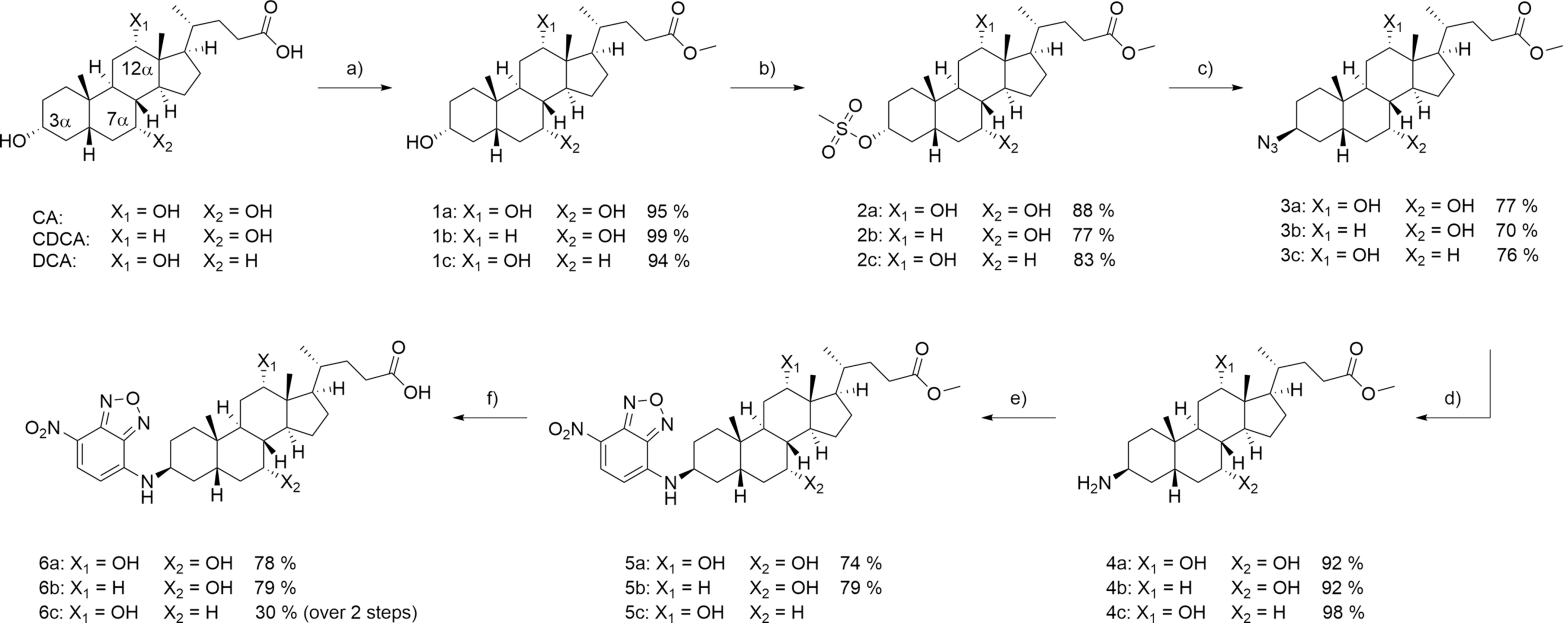
Synthesis of 3β-NBD-BA Derivatives Starting from the Natural BA, CA, CDCA, and DCA^a^

a a) SOCl_2_, dry MeOH, 0 °C → rt, 18 h; b) Et_3_N, CH_3_SO_2_Cl, dry DCM, 0 °C, 2 h; c) NaN_3_, dry DMF, 80 °C, 48 h; d) PPh_3_, H_2_O, THF, 50 °C, 18 h; e) NBDCl, NaHCO_3_, MeOH, 50 °C, 18 h; f) 2N LiOH, MeOH, 40 °C, 3 h.

For 3α-NBD-CA synthesis, it was aspired to adopt the strategy of the β-derivatives adding an additional stereoinversion prior to azide formation (Scheme 2). Mitsunobu reaction was chosen to introduce a leaving group under the inversion of stereochemistry. Therefore, CA methyl ester (**1a**) was reacted with DIAD, triphenylphosphine, and methanesulfonic acid to obtain **7**. Subsequent treatment with sodium azide resulted in the formation of 3α-azido-CA methyl ester (**3d**). Contrary to the literature, the conversion merely gave a yield of 13% over two steps.^42^ It was assumed that the poor yield correlates with the strong acidity of methanesulfonic acid in the Mitsunobu reaction as well as the sterically challenging azide attack from the α-face of the steroid. Intended to optimize the Mitsunobu reaction, TFA was used as the nucleophile exploiting its diminished acidity (**8**). Unfortunately, a significant increase in the yield could not be observed. Hence, it was sought to clarify whether the low yield resulted from the conditions of Mitsunobu reaction. Mitsunobu reaction was therefore performed with CA methyl ester (**1a**) under standard conditions utilizing DIAD, triphenylphosphine, and acetic acid.^45^ The corresponding 3β-acetate was obtained in 81% yield (see Supporting Information), supporting the hypothesis of an ineffective Mitsunobu reaction by enhanced acidity of the aforementioned nucleophiles methanesulfonic acid and TFA. An alternative synthetic approach via Mitsunobu reaction forming the acetate followed by saponification, mesylation, and azide formation was considered. Unfortunately, acetate saponification resulted in cleavage of the methyl ester, making it necessary to modify the ester protection group. However, this approach would require at least two additional steps and thus could not compensate for the low yield of the initial strategy of introducing a leaving group via Mitsunobu reaction. Hence, the synthesis was continued from Mitsunobu reaction using methanesulfonic acid or trifluoroacetic acid. As described for the β-derivatives (see Scheme 1), azide formation, Staudinger reduction, NBD-coupling, and saponification gave 3α-NBD-CA (**6d**) in a six-step synthesis (Scheme 2).

**Scheme 2:**
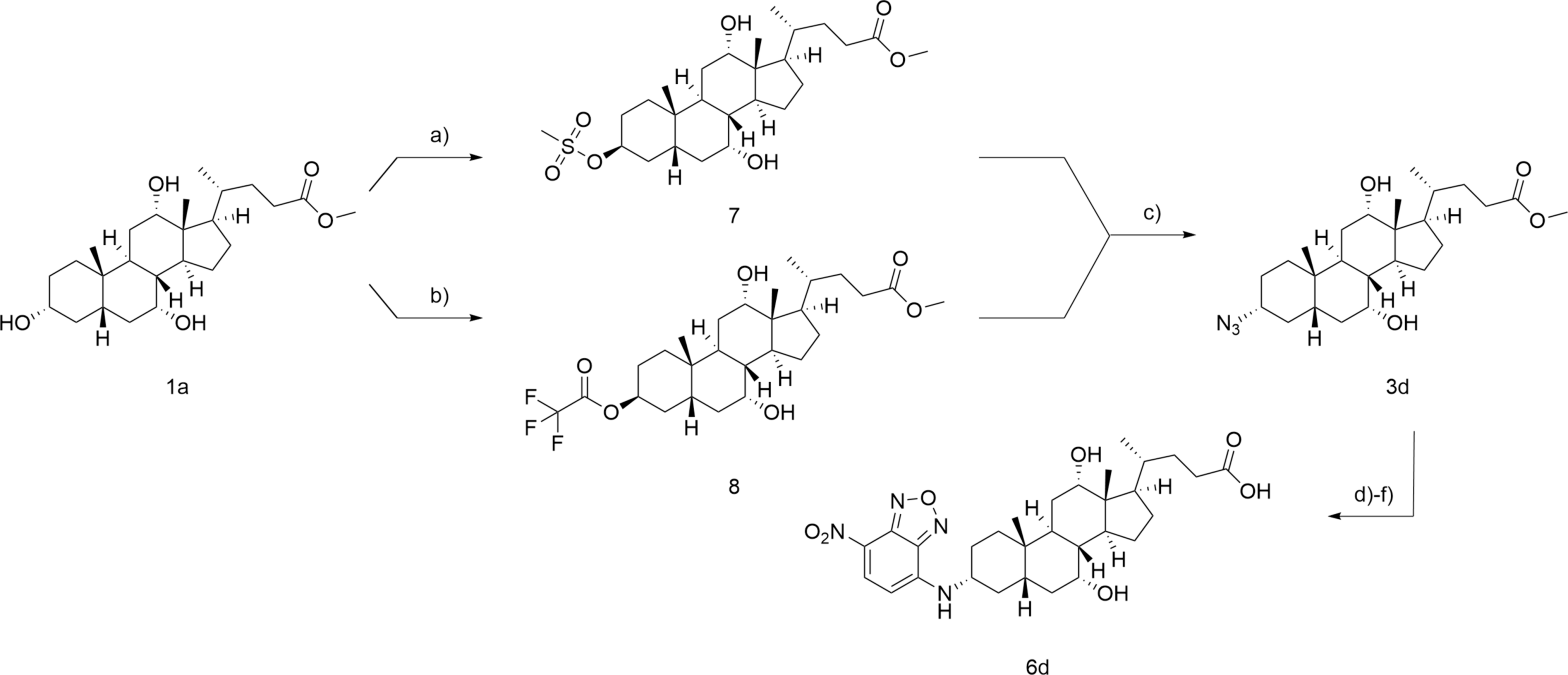
Synthesis of 3α-NBD-CA *via* Mitsunobu Reaction^a^

a a) DIAD, PPh_3_, CH_3_SO_3_H, dry THF, 50 °C, 18 h; b) DIAD, PPh_3_, TFA, dry THF, 50 °C, 18 h; c) NaN_3_, dry DMF, 80 °C, 48 h; d) PPh_3_, H_2_O, THF, 50 °C, 18 h; e) NBDCl, NaHCO_3_, MeOH, 50 °C, 18 h; f) 2N LiOH, MeOH, 40 °C, 3 h.

To further mimic physiological BA most efficiently, a selection of 3-NBD-BA was conjugated with taurine (T) and glycine (G). Unmodified taurine was reacted with 3β-NBD-CA in a peptide coupling reaction using HOBt·H_2_O and TBTU. Due to the terminal sulfonate group, taurine derivatives are soluble in water, whereas solubility in organic solvents is limited. Thus, 3β-NBD-CA remained in the aqueous phase resulting in contamination of the product with various salts and impeding sufficient purification by filtration or column chromatography. The strategy of choice for circumventing those issues was the retention of solubility in organic solvents by sulfonate protection as previously reported by our group for the synthesis of 3β-NBD-TCA.^41^ As protection group, trifluoroethylsulfonic ester was regarded suitable due to its lability toward bases and stability under acidic conditions.^46^ Taurine (**9**) was Boc-protected utilizing tetrabutylammonium hydroxide to provide a hydrophobic counterion (**10**).^47^ *In situ* chlorination followed by protection of the sulfonate moiety with trifluoroethanol resulted in formation of the corresponding sulfonic ester (**11**). By deprotection of the Boc protected amine, compound **12** was obtained. Subsequent peptide coupling to the 3-NBD-BA, **6a**, **6b**, and **6d**, led to the respective sulfonic esters, **13a**, **13b**, and **13d**, which proved to be purifiable by column chromatography. At last, the sulfonate protection group was removed under alkaline conditions,^46^ giving the respective T-conjugated 3-NBD-BA, namely 3β-NBD-TCA (**14a**), 3β-NBD-TCDCA (**14b**), and 3α-NBD-TCA (**14d**) (Scheme 3).

**Scheme 3:**
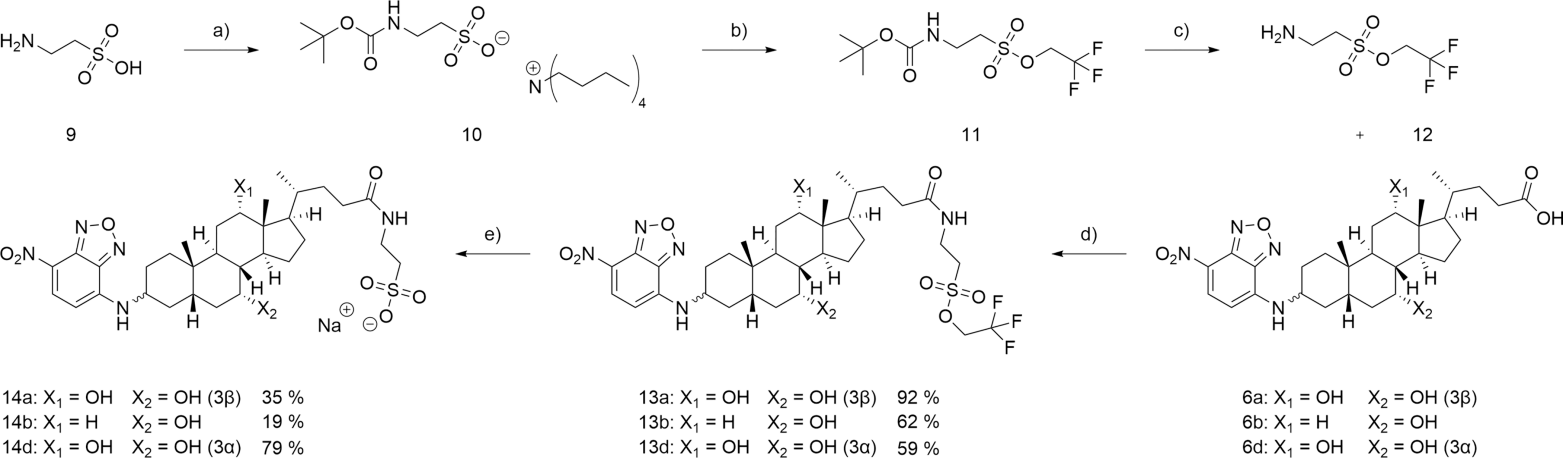
Synthesis of T-Conjugated 3-NBD-BA Derivatives^a^

a a) 40% aq. *n*Bu_4_NOH in H_2_O, Boc_2_O, acetone, rt, 18 h; b) (COCl)_2_ in dry DCM, dry DMF, 0 °C, 1 h, then Et_3_N, CF_3_CH_2_OH in dry DCM, rt, 18 h; c) TFA, DCM, rt, 4 h; d) TBTU, HOBt·H_2_O, Et_3_N, dry DMF, 45 min, then **12** in DMF, rt, 18 h; e) 2N NaOH in MeOH, DCM, rt, 3 h.

G-conjugation was conducted for 3β- and 3α-NBD-CA and proved to be more straightforward. In a peptide coupling, the respective 3-NBD-CA (**6a** or **6d**) was reacted with glycine methyl ester to obtain **15a** and **15d**, respectively. Saponification using sodium hydroxide then gave the respective G-conjugated 3-NBD-BA, namely 3β-NBD-GCA (**16a**) and 3α-NBD-GCA (**16d**) (Scheme 4).

**Scheme 4:**
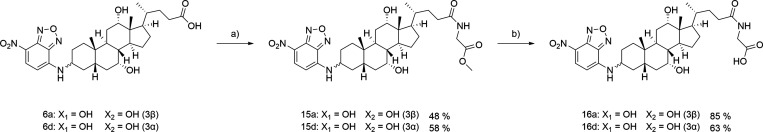
Synthesis of G-Conjugated 3-NBD-CA Derivatives^a^

a a) TBTU, HOBt·H_2_O, Et_3_N, dry DMF, 45 min, then glycine methyl ester · HCl in DMF, rt, 18 h; b) 2N NaOH in MeOH, MeOH, rt, 3 h.

After the synthesis of this set of unconjugated as well as T- and G-conjugated 3β- and 3α-NBD-BA derivatives, namely 3β-NBD-CA (**6a**), 3β-NBD-CDCA (**6b**), 3β-NBD-DCA (**6c**), 3α-NBD-CA (**6d**), 3β-NBD-TCA (**14a**), 3β-NBD-TCDCA (**14b**), 3α-NBD-TCA (**14d**), 3β-NBD-GCA (**16a**), and 3α-NBD-GCA (**16d**) comprehensive transport studies were conducted with all fluorescent BA listed in Figure 1. Prior to the transport studies, the excitation and emission spectra of all fluorescent BA derivatives were determined to obtain the optimal emission and excitation wavelengths for subsequent fluorescent measurements at the fluorescence microscope and the fluorescent reader (Figure 2). The excitation maxima (Ex_Peak_) were at 468–470 nm and the emission maxima (Em_Peak_) at 538–540 nm for all fluorescent NBD-BA.

**Figure 1:**
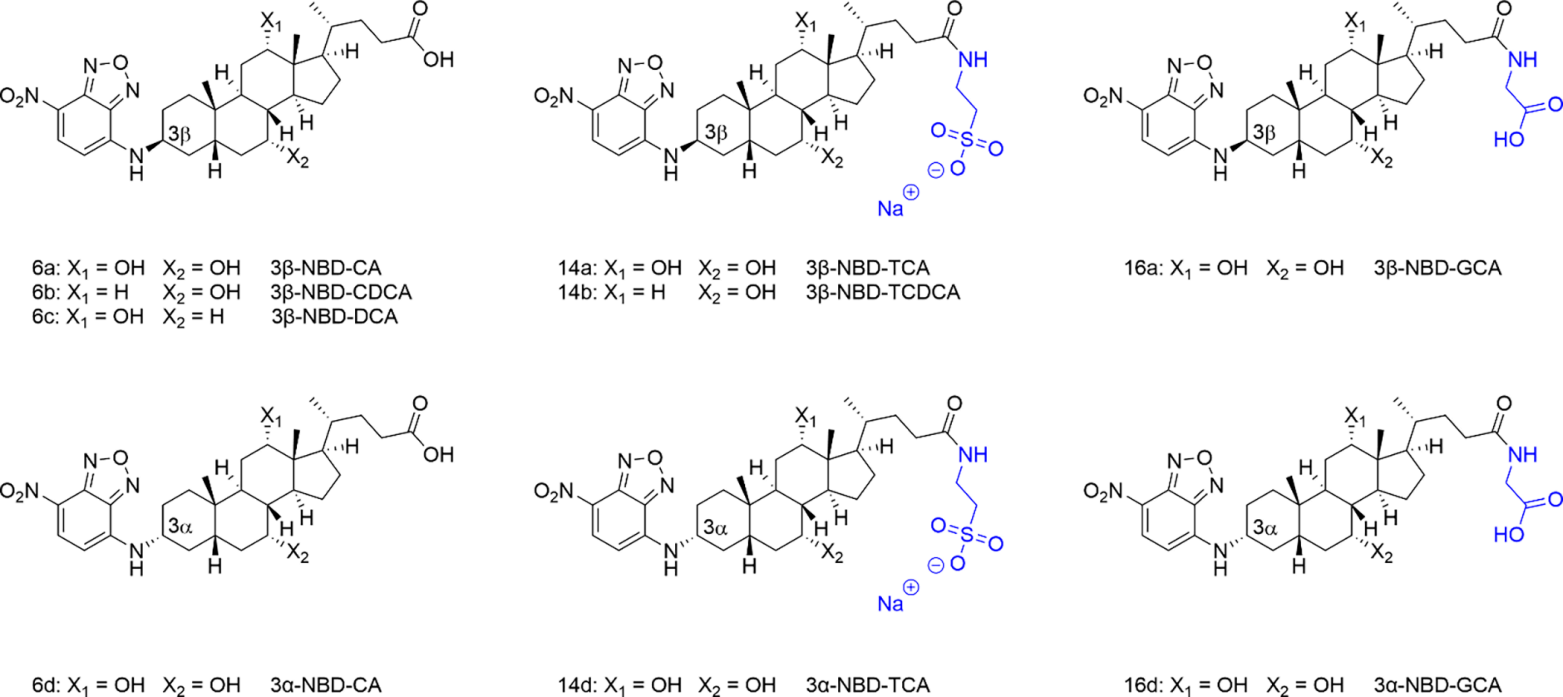
Structures of the unconjugated, T-, and G-conjugated 3-NBD-coupled BA that were synthesized and functionally characterized in the present study. Taurine and glycine side chains are highlighted in blue and the 3α/3β position is indicated. See Supporting Information for additional compound characterization.

**Figure 2:**
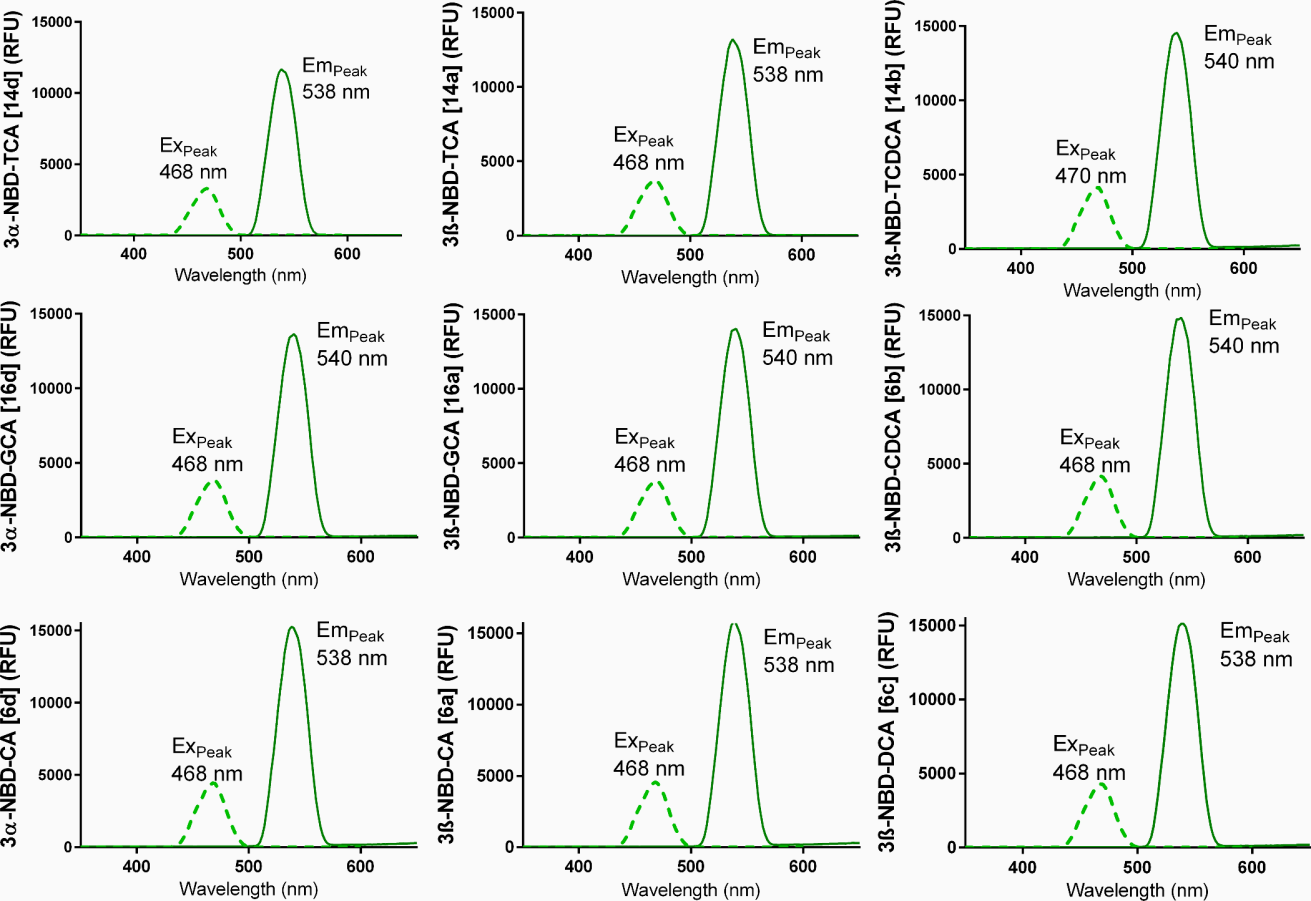
Excitation–emission-spectra of the 3-NBD-BA measured in NaCl-transport buffer at 1 pM compound concentration. The excitation (dotted line, Ex_Peak_) and emission (solid line, Em_Peak_) peak wavelengths (in nm) are indicated for each compound. RFU, relative fluorescence units.

## Results: Biological Evaluation

All synthesized BA were tested as substrates of the BA carriers of the SLC10 carrier family, namely human NTCP, human ASBT, mouse mNtcp, and mouse mAsbt. Human SOAT that does not transport conjugated BA served as control carrier. In addition, two representative carriers of the OATP family were included, namely human OATP1B1 and OATP1B3. All carriers have been stably transfected into HEK293 cells in previous studies and prior to the NBD-BA transport experiments functional expression of the respective carriers was analyzed with prototypic substrates. As shown in Figure 3, NTCP, ASBT, mNtcp, and mAsbt showed significant sodium-dependent transport of [^3^H]­TCA. Among them, the by far highest transport rates were detected for mNtcp with 176-fold higher uptake in the presence of sodium compared to sodium-free control conditions. The other three SLC10 carriers showed comparable transport rates ranging from 30- to 43-fold. As expected, SOAT did not show any transport activity for [^3^H]­TCA, but significant sodium-dependent transport was detected for tritium-labeled dehydroepiandrosterone sulfate ([^3^H]­DHEAS). In addition, [^3^H]­DHEAS was also a substrate of NTCP, but not of ASBT. The functional carrier expression of the OATPs was demonstrated with the prototypic tritium-labeled substrates estrone-3-sulfate ([^3^H]­E1S) for OATP1B1 and bromosulfophthalein ([^3^H]­BSP) for OATP1B3. Both carriers showed significant transport activity compared to non-carrier-expressing HEK293 cells (HEK), although their transport rates were generally lower than those for the SLC10 carriers.

**Figure 3:**
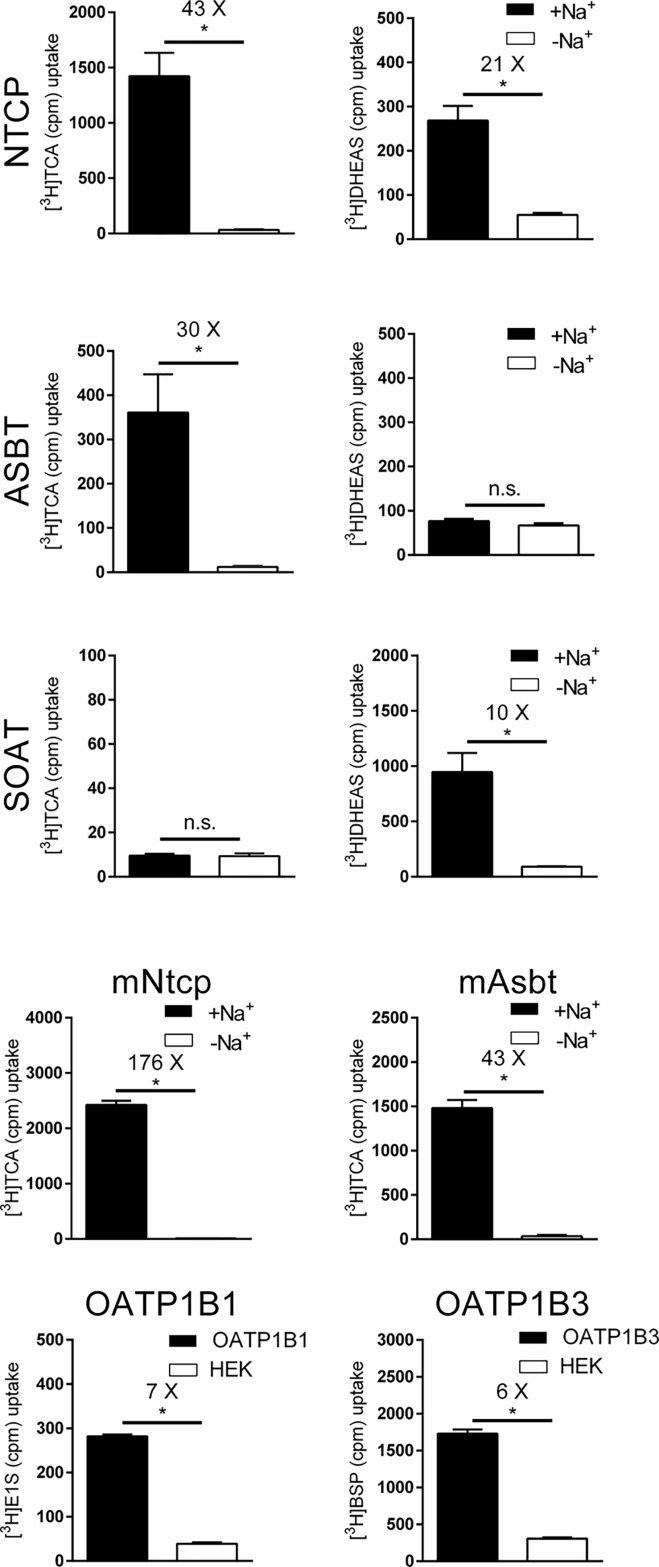
Transport of the indicated [^3^H]-labeled substrates via the carriers NTCP, ASBT, SOAT, mNtcp, mAsbt, OATP1B1, and OATP1B3, all stably expressed in HEK293 cells. Uptake experiments were performed over 10 min at 1 μM substrate concentrations in the presence (+Na^+^) or absence (-Na^+^, negative control) of sodium in the transport buffer as indicated. For human OATP1B1 and OATP1B3 non-carrier-expressing HEK293 cells (HEK) were used as control. Transport ratios are indicated as x-fold higher uptake compared to control. Data represent means ± SD of quadruplicate determinations. *Significantly higher uptake compared to control conditions following Student’s *t*-test with *p* < 0.05; n.s., not significantly different.

Then, all cell lines were cultivated in chamber slides and were grown to confluence to screen for carrier-mediated cellular accumulation of the synthesized NBD-BA. Incubation with the NBD-BA was done in the presence of sodium (+Na^+^) as well as in the absence of sodium (-Na^+^, negative control) for all SLC10 carriers, namely NTCP, ASBT, mNtcp, mAsbt, and SOAT. For OATP1B1 and OATP1B3, nontransfected HEK293 cells were used as negative control. As shown in Figure 4, NTCP-, mNtcp-, and mAsbt-expressing HEK293 cells demonstrated a significant increase in cellular NBD-fluorescence compared to the negative control after incubation with the conjugated NBD-BA, 3β-NBD-TCA (**14a**), 3β-NBD-TCDCA (**14b**), 3α-NBD-TCA (**14d**), 3β-NBD-GCA (**16a**), and 3α-NBD-GCA (**16d**). In contrast, ASBT, SOAT, OATP1B1, and OATP1B3 showed either none or very low background fluorescence. In contrast, incubation with the unconjugated NBD-BA 3β-NBD-CA (**6a**), 3β-NBD-CDCA (**6b**), 3β-NBD-DCA (**6c**), and 3α-NBD-CA (**6d**) resulted in a significant increase in cellular NBD-fluorescence, independent from the control conditions.

**Figure 4:**
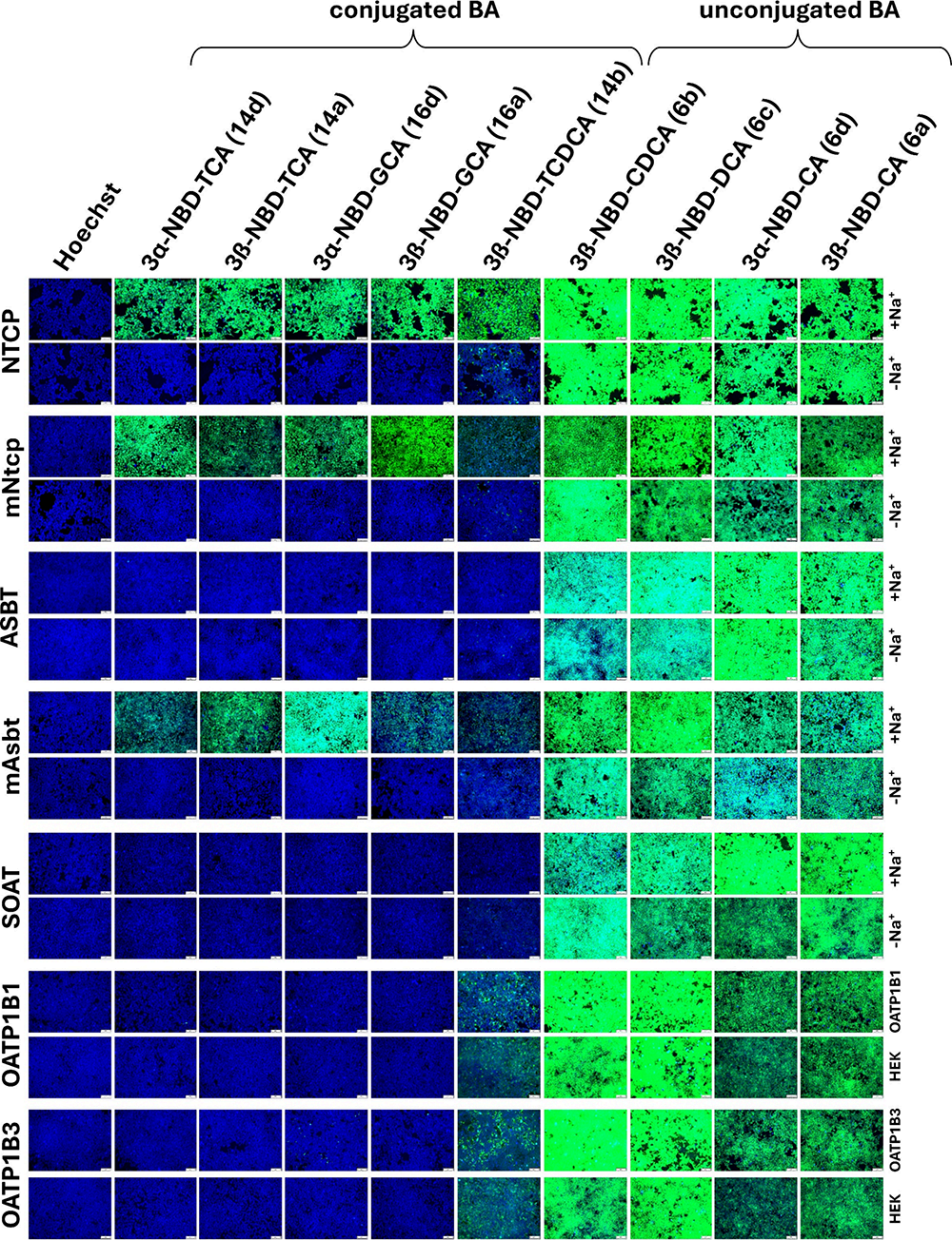
Screening of the cellular uptake of 3-NBD-BA in HEK293 (HEK) cells stably expressing the human carriers NTCP, ASBT, SOAT, OATP1B1, and OATP1B3, or the mouse carriers mNtcp and mAsbt. For the SLC10 carriers, sodium-free conditions (-Na^+^) were used as control. For the OATPs, experiments in non-carrier-expressing HEK293 cells (HEK) served as control. All cell lines were incubated for 10 min with the indicated 3-NBD-BA at 50 μM concentration. Then, cells were washed with PBS and subjected to fluorescence microscopy. Identical microscope settings were used for all images. Scale bar: 75 μm.

For quantification of the transport activity of the synthesized NBD-BA, all cell lines were seeded on 96 well plates and transport experiments were performed under the same control conditions (-Na^+^ control for the SLC10 carriers and nontransfected HEK293 cells for the OATP carriers) as before (Figure 5). For each NBD-BA, a substrate concentration of 25 μM was used, and the uptake phase was terminated after 10 min of incubation at 37 °C. Again, the conjugated NBD-BA, 3β-NBD-TCA (**14a**), 3β-NBD-TCDCA (**14b**), 3α-NBD-TCA (**14d**), 3β-NBD-GCA (**16a**), and 3α-NBD-GCA (**16d**) showed significant transport rates in a sodium-dependent manner in the NTCP-, mNtcp-, and mAsbt-HEK293 cells. The transport ratios ranged from 3-fold to 39-fold. In direct comparison, 3α-NBD-TCA (**14d**) and 3α-NBD-GCA (**16d**) were transported better than their 3β-analogs 3β-NBD-TCA (**14a**) and 3β-NBD-GCA (**16a**), respectively, clearly pointing to a preference for the α-position over the β-position of the 3-NBD-label. In direct comparison between the taurine- or glycine-conjugation of the respective NBD-BA, there was a clear preference for 3α-NBD-GCA (**16d**) over 3α-NBD-TCA (**14d**), at least for NTCP and mAsbt, pointing to a favorable substrate recognition for glycine-conjugated NBD-BA. In contrast, mNtcp showed nearly identical transport rates for the 3α-NBD-GCA (**16d**) and 3α-NBD-TCA (**14d**). Considering the general preference for the α-position of the NBD-label, it is interesting to note that ASBT showed at least some minor but significant sodium-dependent transport activity for 3α-NBD-TCA (**14d**) and 3α-NBD-GCA (**16d**), whereas all β-coupled NBD-BA, 3β-NBD-TCA (**14a**), 3β-NBD-GCA (**16a**) and, 3β-NBD-TCDCA (**14b**), were not transported. The carriers SOAT, OATP1B1, and OATP1B3 were completely inactive for the conjugated NBD-BA.

**Figure 5:**
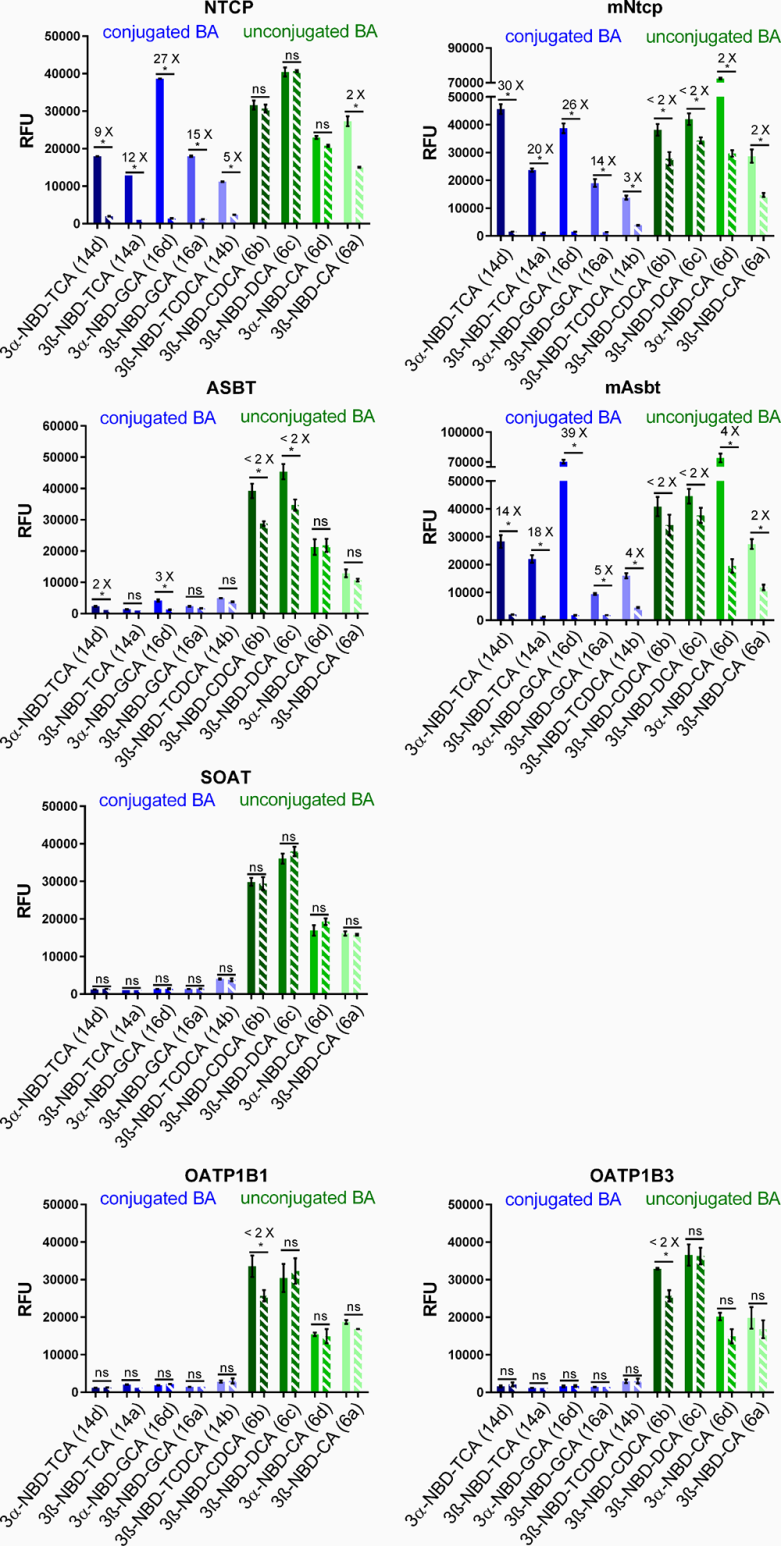
Quantitative uptake experiments with the 3-NBD-BA in HEK293 cells stably expressing the human carriers NTCP, ASBT, SOAT, OATP1B1, and OATP1B3, or the mouse carriers mNtcp and mAsbt. For the SLC10 carriers, all transport experiments were performed in the presence (filled bars) and the absence (hatched bars) of sodium. For the OATP carriers, uptake into carrier-expressing cells (filled bars) was compared to uptake into non-carrier-expressing HEK293 cells (hatched bars). All cell lines were incubated at 37 °C for 10 min with the indicated 3-NBD-BA at 25 μM. Then, cells were washed with PBS and subjected to fluorescence detection. The following fluorescent BA were analyzed: 3α-NBD-TCA (**14d**), 3β-NBD-TCA (**14a**), 3α-NBD-GCA (**16d**), 3β-NBD-GCA (**16a**), 3β-NBD-TCDCA (**14b**), 3β-NBD-CDCA (**6b**), 3β-NBD-DCA (**6c**), 3α-NBD-CA (**6d**), and 3β-NBD-CA (**6a**). Data represent means ± SD of triplicate determinations of representative experiments. Transport ratios are indicated as x-fold higher uptake compared to control. *Significantly higher uptake compared to control according to two-tailed *t*-test (parametric, unpaired) with *p* < 0.01; ns, not significantly different. RFU, relative fluorescence units.

In the case of the unconjugated NBD-BA **6a**–**6d**, there was no clear transport activity for any of the carriers, even if all of them showed relatively high cellular accumulation irrespective of the control conditions. The only exception was a 4-fold higher sodium-dependent transport rate in mAsbt-expressing HEK293 cells for 3α-NBD-CA (**6d**). All other NBD-BA showed no significant differences or transport rates ≤ 2x. Since a meaningful transport rate should be at >2x, the significant 2-fold transport rates for 3β-NBD-CA (**6a**) via NTCP, mNtcp, and mAsbt, as well as for 3α-NBD-CA (**6d**) via mNtcp must be interpreted with caution.

In addition to these comprehensive transport experiments with a single substrate concentration for all NBD-BA, transport kinetic studies were conducted for the NBD-BA that showed significant >2x transport rates in order to determine *K*_*m*_ Michaelis Menten substrate affinity. In a first step, the time-dependent transport of all conjugated NBD-BA was analyzed. Transport via NTCP, mNtcp, ASBT, and mAsbt demonstrated initial linear uptake rates for up to 15 min (see Supporting Information). Based on these findings, an uptake phase of 10 min was selected as the most practical for subsequent kinetic transport measurements. As shown in Figure 6 and Figure 7, the conjugated NBD-BA, 3β-NBD-TCA (**14a)**, 3β-NBD-TCDCA (**14b**), 3α-NBD-TCA (**14d**), 3β-NBD-GCA (**16a**), and 3α-NBD-GCA (**16d**) showed clear saturable concentration-dependent transport kinetics for NTCP, mNtcp, and mAsbt that allowed calculation of Michaelis Menten *K*_*m*_ and *V*_*max*_ values. In contrast, for ASBT, reliable carrier-specific concentration-dependent transport rates and *K*_*m*_ values could only be determined for 3α-NBD-GCA (**16d**). The transport rates for 3β-NBD-TCA (**14a**), 3β-NBD-TCDCA (**14b**), 3β-NBD-GCA (**16a**), and 3α-NBD-TCA (**14d**) were at ≤ 2 across the entire concentration range and were therefore not considered biologically meaningful.

**Figure 6:**
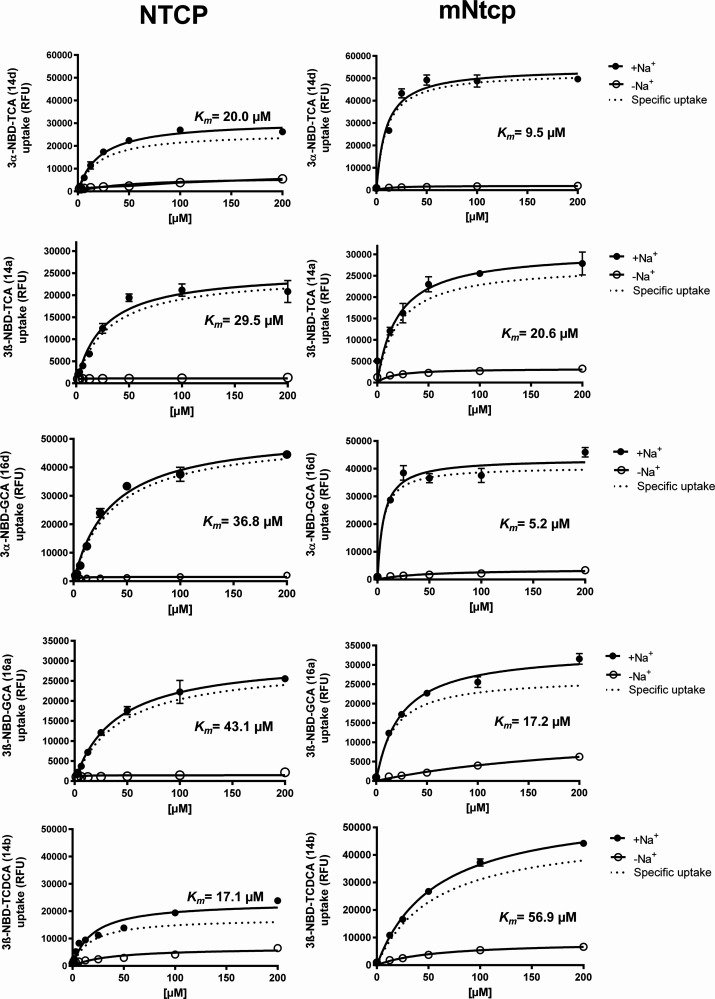
NTCP and mNtcp transport kinetics for conjugated 3-NBD-BA. Concentration-dependent uptake of the indicated NBD-coupled BA was analyzed in HEK293 cells stably expressing human NTCP or mouse mNtcp at increasing substrate concentrations. Control experiments were performed in the absence of sodium (-Na^+^). Uptake was analyzed for 10 min at 37 °C with transport buffer containing the indicated 3-NBD-BA. Afterward, the transport medium was removed, and each cell monolayer was washed and processed for fluorescence detection. Specific uptake was calculated by subtracting the nonspecific uptake in the absence of sodium (-Na^+^, open symbols) from the uptake in the presence of sodium (+Na^+^, closed symbols) and is shown by dotted lines. Values represent means ± SD of quadruplicate determinations. Michaelis Menten *K*_*m*_ values were calculated by nonlinear regression analysis based on the specific uptake data.

**Figure 7:**
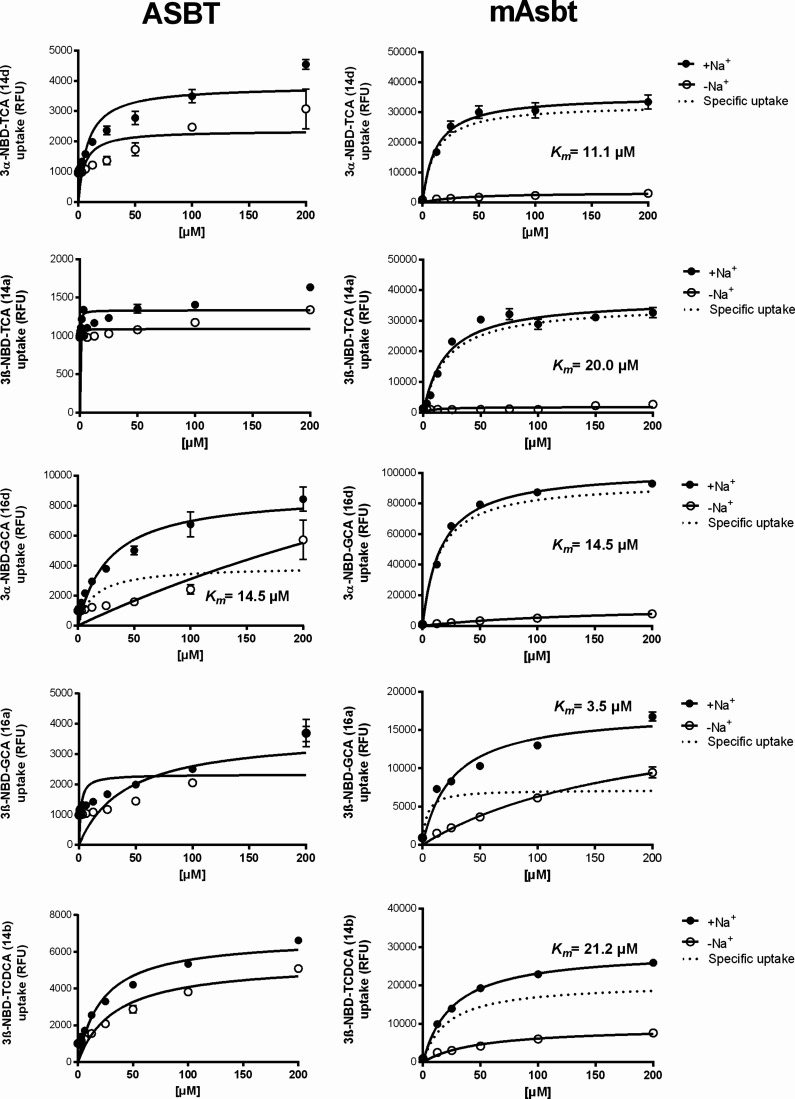
ASBT and mAsbt transport kinetics for conjugated 3-NBD-BA. Concentration-dependent uptake of the indicated NBD-coupled BA was analyzed in HEK293 cells stably expressing human ASBT or mouse mAsbt at increasing substrate concentrations. Control experiments were performed in the absence of sodium (-Na^+^). Uptake was analyzed for 10 min at 37 °C with transport buffer containing the indicated 3-NBD-BA. Afterward, the transport medium was removed, and each cell monolayer was washed and processed for fluorescence detection. Specific uptake was calculated by subtracting the nonspecific uptake in the absence of sodium (-Na^+^, open symbols) from the uptake in the presence of sodium (+Na^+^, closed symbols) and is shown by dotted lines. Values represent means ± SD of quadruplicate determinations. Michaelis Menten *K*_*m*_ values were calculated by nonlinear regression analysis based on the specific uptake data. For ASBT, reliable specific uptake data could only be determined for 3α-NBD-GCA (**16d**).

Among the five analyzed conjugated 3-NBD-BA (namely **14a**, **14b**, **14d**, **16a**, and **16d**), the *K*_*m*_ values ranged between 17.1 and 43.1 μM for NTCP, 5.2–56.9 μM for mNtcp, and 3.5–21.2 μM for mAsbt (Table 1). Considering the wide range of *K*_*m*_ values measured in previous studies for [^3^H]- or [^14^C]-labeled BA molecules as substrates, the *K*_*m*_ values determined in the present study for the NBD-BA fall within this published range. As an example, *K*_*m*_ values for [^3^H]­TCA transport via NTCP varied from 2 to 46 μM depending of the cell model (human hepatocytes, NTCP recombinantly expressed in *Xenopus laevis* oocytes, COS cells, HeLa cells, CHO cells, or HEK293 cells).^48^ Analyzing the transport kinetic data in more detail, 3β-NBD-TCA (**14a**), 3α-NBD-TCA (**14d**), 3β-NBD-GCA (**16a**), and 3α-NBD-GCA (**16d**) differed in means of affinities (*K*_*m*_ values) and maximal transport rates (*V*_*max*_ values) (Table 1). Overall, the degree of variation was much more pronounced for mAsbt, than for NTCP and mNtcp. In direct comparison, the *K*_*m*_ values were generally higher for NTCP compared to mNtcp, except for 3β-NBD-TCDCA (**14b**). Most interestingly, 3β-NBD-TCA (**14a**) showed the highest degree of similarity for the substrate affinities with *K*_*m*_ values of 29.5 μM for NTCP, 20.6 μM for mNtcp, and 20.0 μM for mAsbt. For glyco-conjugated 3-NBD-CA, the 3α-derivative (**16d**) had higher transport rates for NTCP, mNtcp, and mAsbt compared to the 3β-derivative (**16a**) and 3α-NBD-GCA (**16d**) was the only BA for which a *K*_*m*_ value could be determined for ASBT (Table 1, Figure 7).

**Table 1:** Summary of the Mean Transport Kinetic Data *K*_*m*_ (in μM) and *V*_*max*_ (in RFU/10 min) for the Indicated NBD-BA *via* the Carriers NTCP, mNtcp, ASBT, and mAsbt^a^

	**NTCP**		**mNtcp**		**ASBT**		**mAsbt**	
	*K*_*m*_ [μM]	*V*_*max*_ [RFU/10 min]	*K*_*m*_ [μM]	*V*_*max*_ [RFU/10 min]	*K*_*m*_ [μM]	*V*_*max*_ [RFU/10 min]	*K*_*m*_ [μM]	*V*_*max*_ [RFU/10 min]
3α-NBD-TCA **(14d)**	20.0	25,742	9.5	52,588			11.1	32,567
3β-NBD-TCA **(14a)**	29.5	24,712	20.6	27,562			20.0	35,190
3α-NBD-GCA **(16d)**	36.8	50,932	5.2	40,662	14.5	3,945	14.5	94,113
3β-NBD-GCA **(16a)**	43.1	29,152	17.2	26,736			3.5	7,175
3β-NBD-TCDCA **(14b)**	17.1	17,371	56.9	48,820			21.2	20,535

a Additional 95% confidence intervals for the mean *K*_*m*_ and *V*_*max*_ values are provided in the Supporting Information. For ASBT, reliable transport kinetic data could only be determined for 3α-NBD-GCA (**16d**).

The lack of efficient transport of the conjugated NBD-BA via ASBT was unexpected considering the high transport rates of the mouse homologue mAsbt. This effect needs further explanation and investigation. The NBD-label might sterically block substrate binding to the transporter protein or efficient substrate translocation. However, it is surprising that this effect is only seen for ASBT, while mAsbt, mNtcp, and NTCP show efficient transport for all the conjugated NBD-BA of the present study. To address this question, we directly compared the AlphaFold structures of all four proteins and analyzed the volumes of potential substrate binding site cavities. As shown in Figure 8, NTCP, mNtcp, and mAsbt revealed quite large solvent accessible cavities with total volumes of 387, 487, and 306 Å^3^, respectively. In contrast, ASBT revealed a more compact structure with a very low volume of the potential substrate binding site cavity of only 115 Å^3^.

**Figure 8:**
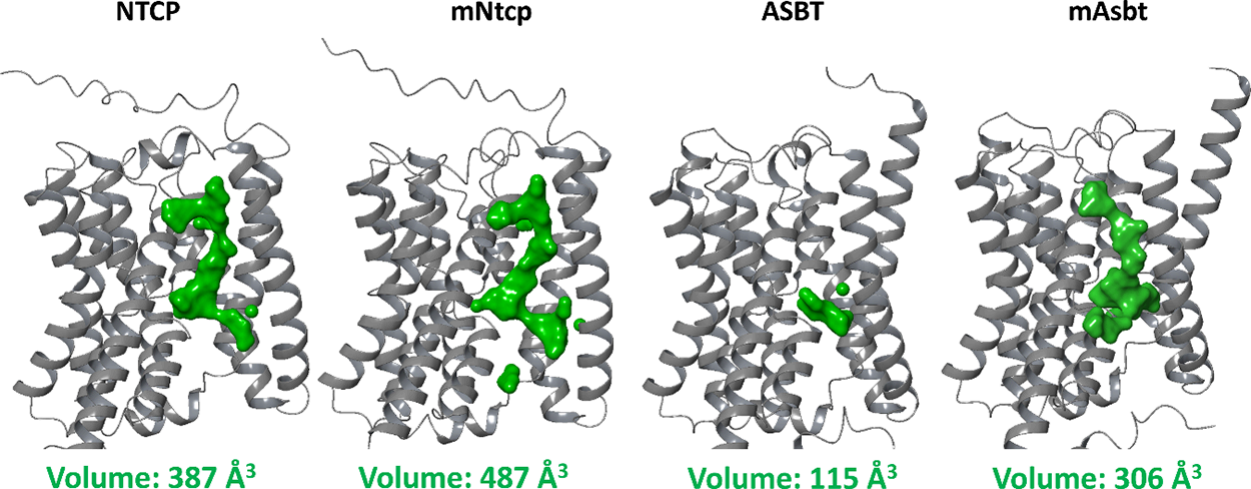
Comparison of the solvent accessible potential substrate binding site cavities of human NTCP (UniProt Q14973), mouse mNtcp (UniProt O08705), human ASBT (UniProt Q12908), and mouse mAsbt (UniProt P70172). The volumes of the potential substrate binding site cavities are depicted in green and were calculated using MAESTRO SiteMap. Total volumes are indicated for each carrier.

As shown in Figure 4 and Figure 5, we observed very low carrier-independent accumulation of the conjugated NBD-BA, but high cellular accumulation of the unconjugated NBD-BA. To further investigate this difference, the logP values of all NBD-BA were experimentally determined using octanol/water partition coefficients. In addition to the NBD-BA, [^3^H]­TCA and [^3^H]­CA were included in this analysis as reference compounds for 3α-NBD-TCA (**14d**) and 3α-NBD-CA (**6d**), respectively. As illustrated in Figure 9A, the logP­(o/w) values were generally lower for the conjugated NBD-BA compared with the unconjugated NBD-BA, pointing to higher lipophilicity of the unconjugated NBD-BA. A direct comparison between [^3^H]­TCA with logP­(o/w) of −0.8 and 3α-NBD-TCA (**14d**) with logP­(o/w) of 0.03, suggests that the NBD-label slightly increases the lipophilicity of the TCA molecule. Similarly, 3α-NBD-CA (**6d**) exhibited a higher logP­(o/w) of 2.8 compared to [^3^H]­CA with logP­(o/w) of 0.7 (Figure 9A). Of note, the logP­(o/w) values correlated quite well with the quantitative carrier-independent cellular fluorescence levels that were determined for the SLC10 carriers in the absence of sodium or in nontransfected HEK293 control cells (Figure 9B). In addition, it was interesting to note that the conjugated NBD-BA after 10 min of carrier-mediated transport via NTCP, mNtcp, or mAsbt reached fluorescence levels that were obtained for the unconjugated NBD-BA independent from carrier overexpression (Figure 5).

**Figure 9:**
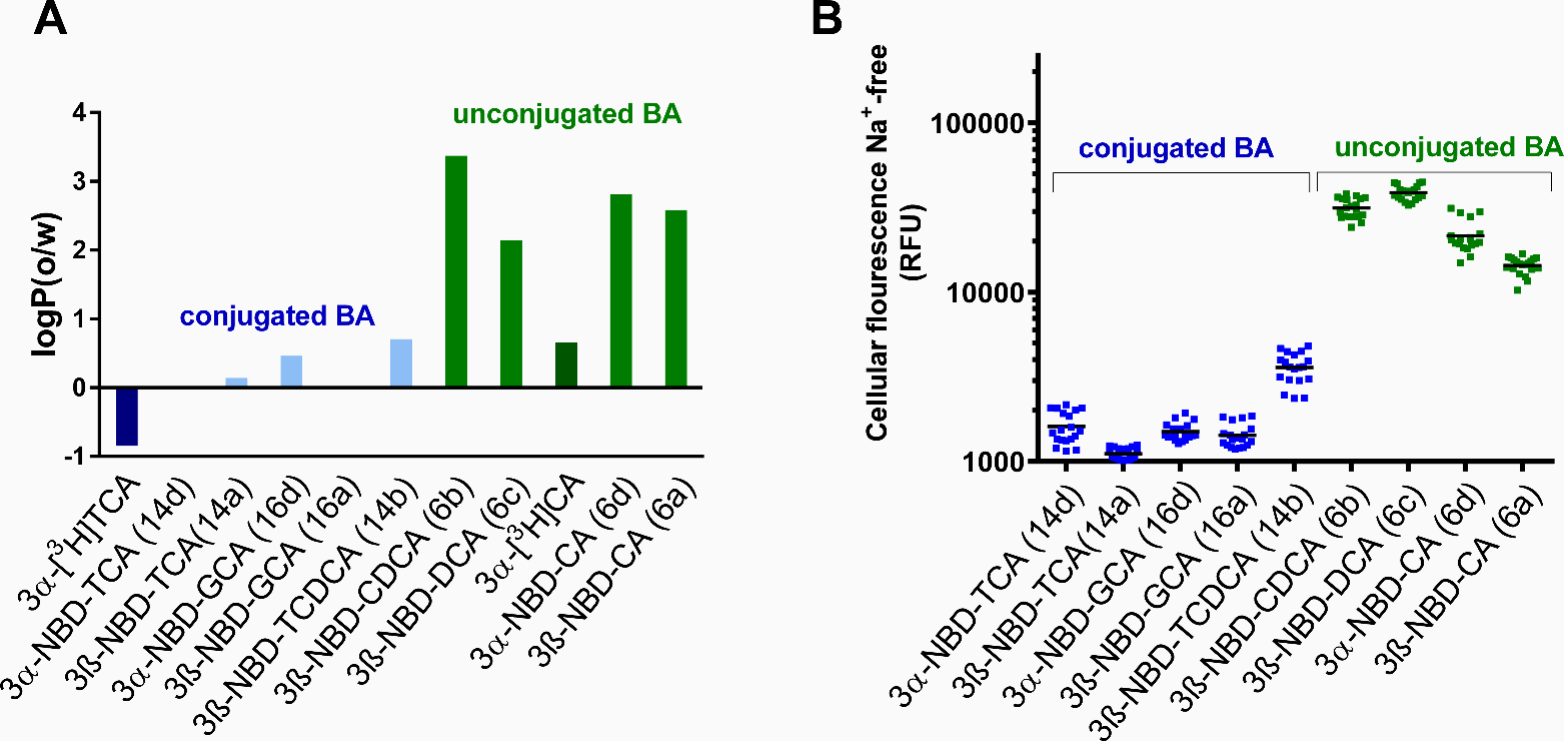
Membrane permeability of the 3-NBD-BA and logP­(o/w) values. (**A**) The logP­(o/w) values were measured in 1 to 1 mixture of 500 μL water and 500 μL 1-octanol. For each 3-NBD-BA, 1 μL of a 25 μM concentration was added. In the case of [^3^H]­TCA and [^3^H]­CA, 1 μL of respective 1 μM concentrations was used. The lipophilic and hydrophilic phases were separated by centrifugation and both phases were analyzed by fluorescence detection or liquid scintillation, respectively. (**B**) The scatter plot shows the cellular 3-NBD-BA accumulation at 25 μM concentration in the absence of sodium in NTCP, ASBT, SOAT, mNtcp, mAsbt, and in nontransfected HEK293 cell lines.

As mentioned above, NTCP and ASBT are established drug targets. So, finally we investigated if the 3-NBD-BA are appropriate probe substrates for inhibitor testing approaches. We used the well-established NTCP inhibitor cyclosporine A to inhibit the transport of 3α-NBD-GCA (**16d**) via NTCP and mNtcp at increasing inhibitor concentrations. As shown in Figure 10, cyclosporine A showed significant inhibition with comparable IC_50_ values for NTCP and mNtcp of 2.8 μM and 2.7 μM, respectively. Even if the transport rates for human ASBT were quite low, transport inhibition experiments with 3α-NBD-GCA (**16d**) as the substrate and troglitazone as the reference inhibitor exhibited comparable IC_50_ values of 19.5 μM for ASBT and 18.9 μM for mAsbt.

**Figure 10:**
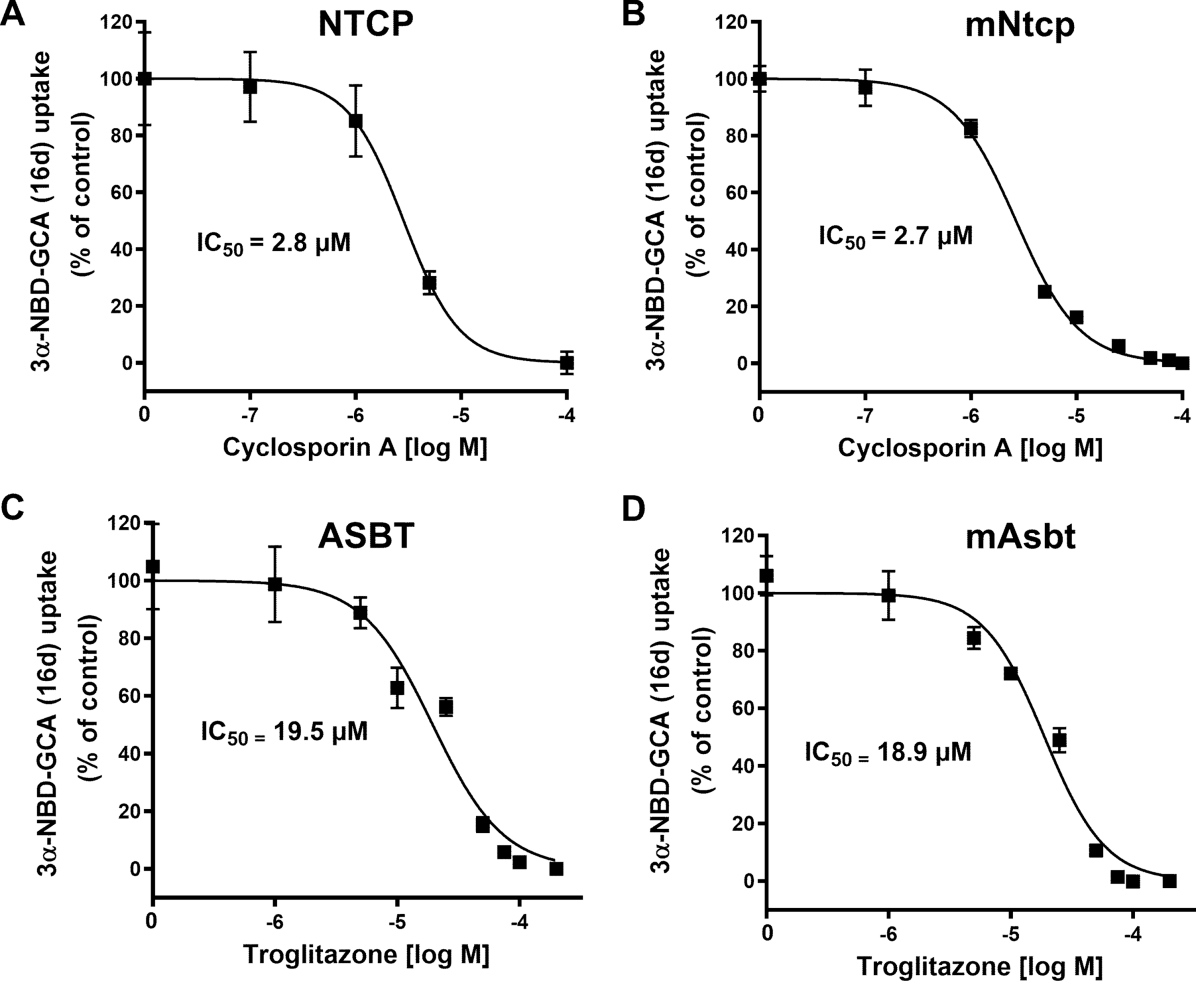
Inhibition of 3α-NBD-GCA (**16d**) transport with prototypic inhibitors, namely cyclosporine A and troglitazone. HEK293 cells stably expressing human NTCP (**A**), mouse mNtcp (**B**), human ASBT (**C**), or mouse mAsbt (**D**) were used for transport experiments with 3α-NBD-GCA (**16d**) as the substrate. Fluorescence data in the absence of inhibitors were set as 100% and the fluorescence levels in sodium-free transport buffer were set to 0%. Cyclosporine A was used as inhibitor of NTCP and mNtcp, and troglitazone as inhibitor of ASBT and mAsbt, respectively. Half-maximal inhibitory concentrations (IC_50_) were calculated by nonlinear regression analysis. The mean IC_50_ values are depicted in the Figure. The following 95% confidence intervals were determined: 2.2–3.8 μM for cyclosporine A inhibition of NTCP, 2.0–3.5 μM for cyclosporine A inhibition of mNtcp, 16.3–23.2 μM for troglitazone inhibition of ASBT, and 16.8–21.3 μM for troglitazone inhibition of mAsbt.

## Discussion

The investigation of BA transport processes in the liver, kidney, and intestine *in vivo* and *in vitro* primarily requires the traceability of the BA molecules of interest. BA quantification and profiling from biological samples can be performed with analytical methods such as LC-MS/MS,^49–51^ but this requires immense analytical and technical efforts. Therefore, radiolabeled BA such as [^3^H]­TCA are widely used to study BA transport.^11,26,52^ Even if radiolabeled BA perfectly mimic the parent molecule regarding structure and physicochemical properties, the handling of radioisotopes requires specific radiological safety protocols as well as appropriate equipment and facilities. Furthermore, real-time monitoring of the body distribution of radiolabeled BA in animal models is limited and their use for dynamic liver function tests in patients is excluded.^53,54^ The use of fluorescent BA derivatives on the other hand enables broader application, including *in vitro* BA transport studies using fluorescence microscopy or fluorescence readers (see Figures 4 and 5) even in a high-throughput setting. In addition, *in vivo* application is possible, e.g. for *in situ* rat liver perfusion^28^ or for intravital fluorescence microscopy^41^ to monitor hepatobiliary elimination and distribution of BA in animal models in real-time. Finally, fluorescent BA molecules are more convenient to handle. However, attachment of a fluorophore moiety to a BA molecule significantly changes the chemical structure with potential consequences for the physiochemical properties of the BA conjugate.^54–56^ As a consequence, substrate recognition by BA transporters could be abolished because the fluorophore sterically blocks the binding of the fluorescent BA conjugate to the substrate binding site. Therefore, not all fluorescent BA derivatives are generally suitable probe substrates for *in vitro* and *in vivo* experiments. Many different fluorophore-coupled BA have already been developed that are discussed below in more detail. Unfortunately, not all of them have been comprehensively tested to answer the question if and to what extend the fluorophore label really affects the transport behavior and physiochemical properties of the fluorescent BA molecules.^57,58^

The present study used NBD as the labeling fluorophore to synthesize a series of 3-NBD-BA with the fluorophore attached to position 3 of the steroid nucleus. Even if 3-NBD-BA have already been synthesized in previous studies,^28,44,59–61^ their interactions with particular BA carriers have not been analyzed in detail so far. In a previous study, we already used 3β-NBD-TCA for *in vitro* and *in vivo* testing. In this study, 3β-NBD-TCA showed similar transport behavior at mNtcp and mAsbt compared to the radiolabeled [^3^H]-3α-TCA and was well suitable for intravital imaging of BA distribution in the liver and kidney.^41^ The present study aimed to investigate whether 3-NBD-BA are also accepted as transport substrates of relevant human BA transporters, and if the orientation of the 3-NBD group in α- or β-position as well as the type of side-chain conjugation makes a difference for substrate recognition.

The main findings of the present study are as follows: (I) All conjugated 3-NBD-BA – namely 3β-NBD-TCA (**14a**), 3β-NBD-TCDCA (**14b**), 3α-NBD-TCA (**14d**), 3β-NBD-GCA (**16a**), and 3α-NBD-GCA (**16d**) – were significantly transported by NTCP, mNtcp, and mAsbt in a sodium-dependent manner. The *K*_*m*_ values ranged from 17.1 to 43.1 μM for NTCP, 5.2–56.9 μM for mNtcp, and 3.5–21.2 μM for mAsbt, aligning with previously published data.^48^ The transport rates were generally higher for the 3α-NBD compared to the 3β-NBD derivatives, or they remained at the same level, indicating a preference for the α-position over the β-position of the 3-NBD-label. However, among all tested NBD-BA, 3β-NBD-TCA (**14a**) showed the most congruent *K*_*m*_ values for NTCP, mNtcp, and mAsbt. (II) ASBT exhibited biologically relevant, sodium-dependent transport activity only for 3α-NBD-GCA (**16d**) with a *K*_*m*_ value of 14.5 μM. In contrast, 3α-NBD-TCA (**14d**), 3β-NBD-TCA (**14a**), 3β-NBD-GCA (**16a**), and 3β-NBD-TCDCA (**14b**) were either not transported or did not reach consistent transport rates of >2x. This suggests inefficient transport via ASBT when BA are 3-NBD coupled. Conversely, all these conjugated 3-NBD-BA were well transported via mAsbt. This significant species difference between ASBT and mAsbt may be attributed to the exceptionally low volume of the potential substrate binding site cavity of ASBT. While the smaller parent BA molecules might fit into this volume-restricted binding cavity, the NBD label enlarges the BA molecules in a way that sterically hinders substrate binding. (III) Consequently, 3α-NBD-GCA (**16d**) is the most suitable probe substrate for comparative transport studies involving all carriers: NTCP, ASBT, mNtcp, and mAsbt. Notably, 3α-NBD-GCA (**16d**) has been successfully used as a probe substrate for the inhibition of NTCP and ASBT with the established inhibitors cyclosporine A and troglitazone, respectively. Moreover, this compound shows promise as a probe substrate for screening approaches to identify novel NTCP and ASBT inhibitors. However, this would require more extensive inhibition studies with different classes of transport inhibitors. (IV) SOAT, OATP1B1, and OATP1B3 were completely inactive for all the conjugated 3-NBD-BA. (V) The unconjugated 3-NBD-BA – 3β-NBD-CA (**6a**), 3β-NBD-CDCA (**6b**), 3β-NBD-DCA (**6c**), and 3α-NBD-CA (**6d**) – were highly lipophilic with logP values >2 and demonstrated strong cellular accumulation, which may occur via passive diffusion or via BA carriers naturally expressed in HEK293 cells. Notably, the carrier-mediated transport of the conjugated NBD-BA achieved cellular fluorescence levels like those obtained by the unconjugated NBD-BA without carrier overexpression. This underscores the significant role of membrane carriers such as NTCP and ASBT for the cellular transport and distribution of hydrophilic conjugated BA.

In addition to the 3 position, previous studies coupled the NBD-label to the BA molecule also at different positions of the steroid nucleus (see Figure 11). A first series of 3β-, 3α-, 7β-, 7α-, 12β-, and 12α-NBD-CA and -TCA derivatives had been synthesized and characterized already in the early 1990s.^59,62^ Distribution of these NBD-coupled BA was investigated e.g. by *in situ* liver perfusion with bile duct cannulation.^44,59,62^ In these experiments, 3β-NBD-TCA showed rapid hepatobiliary elimination and 95% of the amount injected into the mesenterial vein was recovered in the bile within 30 min.^59^ In contrast, the 3α-NBD-TCA derivative was completely transformed within the liver cells to a polar fluorescent metabolite that showed sparse secretion into bile. Based on this, it can be concluded that even if the orientation of the NBD label in 3β-orientation does not reflect the physiological 3α-orientation of the hydroxy group at this position, 3β-NBD-TCA is the most appropriate 3-NBD-BA derivative for *in vivo* studies on hepatobiliary BA elimination and distribution^41^ as well as for comparative transport studies on mNtcp and mAsbt due to comparable transport kinetics (Figure 6 and 7).

**Figure 11:**
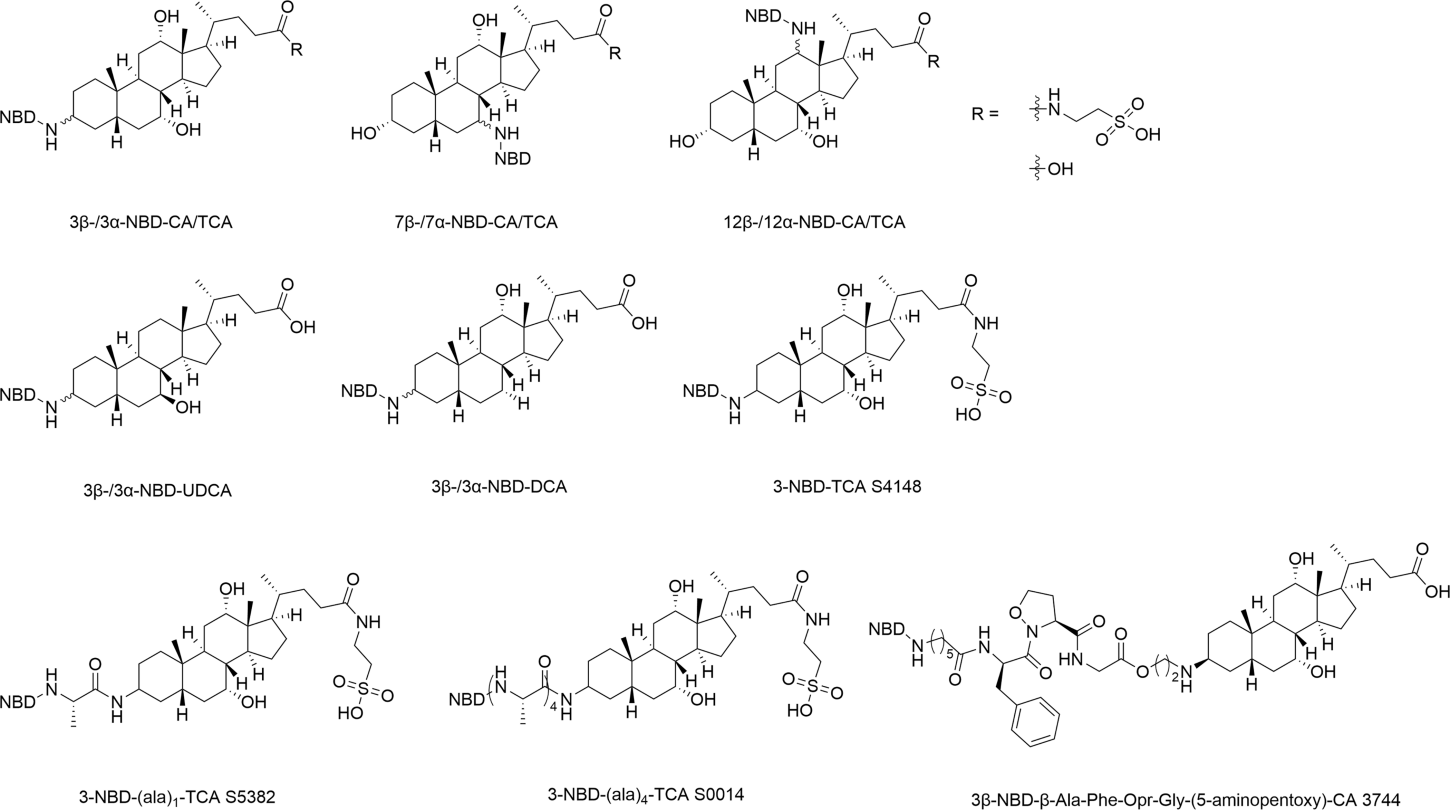
Fluorescent BA derivatives with the NBD-fluorophore coupled to the steroid core.

Subsequent studies used freshly isolated rat hepatocytes and measured the transport of 3β-, 3α-, 7β-, and 7α-NBD-CA by fluorescent flow cytometry. In these experiments, 3α-NBD-CA was the most efficient derivative regarding its hepatocellular transport. This transport was significantly inhibited by troglitazone and cyclosporine A, both being well-known inhibitors of NTCP/mNtcp, suggesting that mNtcp is involved in the hepatic transport process.^60^ Even if we also analyzed 3α-NBD-CA and 3β-NBD-CA in the present study, both compounds showed strong carrier-independent accumulation in the HEK293 cells so that transport studies could not be performed. To confirm the role of mNtcp for 3-NBD-CA transport, a cell model with lower unspecific NBD-BA accumulation must be used for recombinant mNtcp expression. Transport studies with 3β- and 3α-NBD-DCA and -UDCA (Figure 11) in Caco2 cells also confirmed more efficient transport of the respective 3α-NBD derivatives compared to the 3β-NBD derivatives. However, carrier-specific transport experiments were not performed in this study.^61^ In comparison to the 3β-NBD-TCA that showed significant transport via NTCP and mNtcp in the present study, 7β-NBD-TCA has not been found to be a substrate of NTCP when expressed in LLC-PK1 cells,^31^ further supporting the 3-position as the favorable position for NBD-labeling of BA.

Intending to develop drug-BA conjugates for liver-specific drug targeting, several additional 3-NBD-BA derivatives have been developed, where the NBD-label was either directly attached to the steroid core or separated by linker peptides of different length and sequence. Studies with these BA derivatives were performed *in vivo* in the rat ileum perfusion model with bile duct cannulation and by *in situ* rat liver perfusion with bile duct cannulation.^28,63^ Petzinger et al. analyzed 3-NBD-TCA (S4148), and the peptide linker derivatives with either one (L-(Ala)_1_, S5382) or four (L-(Ala)_4_, S0014) alanine residues (Figure 11).^28^ Unfortunately, this publication does not state if the 3-NBD label at the TCA molecule is attached in α-configuration, as in compound 3α-NBD-TCA (**14d**), or in β-configuration, as in compound 3β-NBD-TCA (**14a**). By means of *in situ* rat liver perfusion all these 3-NBD-BA derivatives were rapidly detected in the bile within few minutes and showed cumulative hepatobiliary elimination in the rank order S5382 > S4148 > S0014.^28,63^ In addition, S4148 was also rapidly detected in the bile after *in situ* ileal perfusion and revealed a very similar behavior compared to the natural BA with 76% of the compound being secreted into bile within 120 min.^28,63^ This data clearly confirms active BA transport for 3-NBD-TCA (S4148) in the intestine and the liver. As 3α-NBD-TCA (**14d**) and 3β-NBD-TCA (**14a)** have been identified as substrates of mAsbt and mNtcp, both carriers seem to be involved in this process. In another study, an even larger peptide linker was used, consisting of the β-Ala-Phe-Opr-Gly tetrapeptide and an additional 5-aminopentoxy spacer attached to the 3β-position of CA (compound 3744) (Figure 11). After instillation into a closed loop ileal segment of a rat this large NBD-BA derivative could readily be detected in the bile with a secretion profile like a natural bile acid.^28,63^ This data also suggests involvement of active BA transporters in the intestine and the liver. However, no specific transport studies were performed at isolated BA carriers and so the involvement of mNtcp and mAsbt in this process remains speculative. But these studies clearly underline that 3-NBD-coupling is favorable for the transport behavior of the BA conjugate.

Other studies coupled the fluorophore to the BA side chain using different linker moieties. As an example, NBD was side chain-coupled to CA and CDCA with ethylene diamine as the linker to obtain CenNBD and CDCenNBD (Figure 12).^56^ While CDCenNBD was well transported by OATP1B1, OATP1B3, and OATP2B1, and CenNBD showed at least some low-capacity transport via OATP1B1 and OATP2B1, both compounds were not transported by NTCP and ASBT.^56^ This is in clear contrast to the 3-NBD-BA analyzed in the present study that were transported by NTCP and in part also by ASBT, but not by OATP1B1 and OATP1B3. Based on this it can be concluded that not the NBD-fluorophore itself, but its site of coupling determines the substrate recognition for the BA carriers of the SLC10 and OATP families. Other studies used lysine as a linker to obtain cholyl-(Nε-NBD)-lysine (CA-NBD-L) and its analogues UDCA-NBD-L and CDCA-NBD-L. These fluorescent BA derivatives were intensively investigated in the perfused rat liver model and in isolated rat hepatocytes.^28,57,58,64^ Later on, CDCA-NBD-L has been identified as common substrate of OATP1B1, OATP1B3, ASBT, and NTCP in carrier-transfected HepG2, CHO, or HEK293 cells, respectively.^65^ Other studies analyzed the transport of the BA derivative tauro-nor-THCA-DBD utilizing the NBD-analogue 4-*N*,*N*-dimethylaminosulfobenzo-2-oxa-1,3-diazole (DBD) as fluorophore. In carrier-transfected CHO cells or *Xenopus laevis* oocytes a significant transport for OATP1B1, OATP1B3, NTCP, and ASBT was found for tauro-nor-THCA-DBD.^55^ Based on this, CDCA-NBD-L or tauro-nor-THCA-DBD might be the most appropriate probe substrate for comparative transport on NTCP, ASBT, OATP1B1, and OATP1B3 among the group of side chain-NBD/DBD-coupled BA. Apart from NBD or DBD, other studies used fluorescein as the fluorophore for side chain-coupling of BA. Cholyl-glycyl-amido-fluorescein (CGamF)^28,30,31,57,58,64,66,67^ and cholyl-l-lysyl-fluorescein (CLF)^29,32,64,68^ are the most frequently examined compounds from this group (Figure 12). Both compounds showed intact hepatobiliary elimination in animal models^28,29,66,68^ but showed differences in their transport behavior. While CGamF is a substrate of NTCP, OATP1B1, and OATP1B3,^30,31^ CLF was not transported by NTCP, but only via OATP1B1 and OATP1B3.^29^

**Figure 12:**
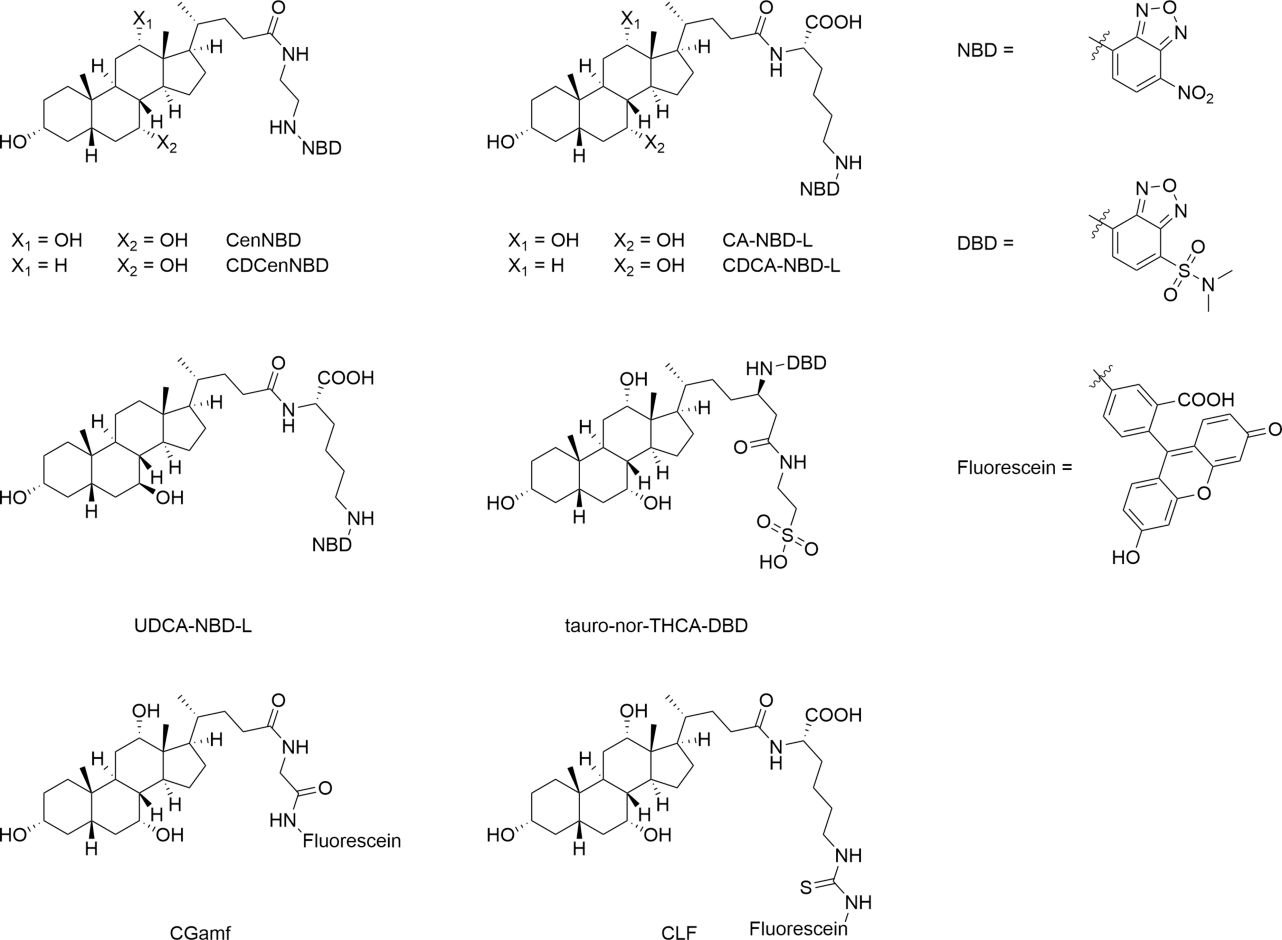
Fluorescent BA derivatives with the NBD/DBD- or fluorescein-fluorophore coupled to the BA side chain.

## Conclusions

Over the past three decades, numerous fluorophore-coupled BA have been synthesized and analyzed by coupling fluorophores like NBD, DBD, or fluorescein to either the BA steroid core or the side chain. While side chain-coupling prevents taurine and glycine conjugation, coupling at the 3 position has been favorable in some studies.^28,63^ All these studies have their limitations by either focusing solely on the *in vivo* disposition of the fluorescent BA derivatives – *e.g*. by *in situ* liver perfusion in animal models or by transport studies in carrier-expressing cell lines – with a clear restriction to the human BA carriers. The present study contributes to this field by providing detailed synthesis protocols for 3-NBD coupling of different BA and comprehensive transport studies on the human carriers NTCP, ASBT, SOAT, OATP1B1, and OATP1B3 as well as the mouse carriers mNtcp and mAsbt. Surprisingly, 3β-NBD-coupling of BA, despite not aligning with the natural 3α-orientation of the hydroxy group in all natural BA molecules, resulted in suitable substrates – 3β-NBD-TCA (**14a**) and 3β-NBD-GCA (**16a**) – for the carriers NTCP, mNtcp, and mAsbt. Even more intriguing is the finding that 3-NBD-coupled BA are generally not well transported by human ASBT, except for 3α-NBD-GCA (**16d**). Based on the data from the present study, we suggest the use of 3β-NBD-TCA (**14a**) as a probe substrate for comparative transport studies involving human NTCP, mNtcp, and mAsbt, as well as the use of 3α-NBD-GCA (**16d**) as the most promising probe substrate for fluorescence-based inhibitor screening studies to identify novel NTCP and ASBT inhibitors of pharmacological interest.

## Experimental Section

### Stably Transfected HEK293 Cell Lines for Human NTCP, ASBT, SOAT, OATP1B1, and OATP1B3 as well as Mouse mNtcp and mAsbt

HEK293 cell lines stably transfected with the full open reading frames of human NTCP, ASBT, SOAT, OATP1B1, and OATP1B3 as well as mouse mNtcp and mAsbt were generated, used, and cultured as described before.^11,41,69–71^ Nontransfected Flp-In HEK293 cells (Thermo Fisher Scientific, Waltham, MA, USA) served as control. All cell lines were maintained at 37 °C, 5% CO_2_ and 95% humidity in DMEM/F-12 medium (Thermo Fisher Scientific) supplemented with 10% fetal calf serum (Sigma-Aldrich, St. Louis, MO, USA), 4 mM l-glutamine (PAA, Cölbe, Germany) and penicillin/streptomycin (PAA).

### Transport and Inhibition Assays with Fluorescent Bile Acids

All stable cell lines were functionally characterized with respective prototypic tritium-labeled substrates, namely [^3^H]­taurocholic acid ([^3^H]­TCA), [^3^H]­dehydroepiandrosterone sulfate ([^3^H]­DHEAS), [^3^H]­estrone-3-sulfate ([^3^H]­E1S), or [^3^H]­bromosulfophthalein ([^3^H]­BSP), all of which were obtained from American Radiolabeled Chemicals (St. Louis, United States) via BIOTREND Chemikalien GmbH (Cologne, Germany). Fluorescence microscopy was performed for transport screening of the fluorescent BA by a Leica DMI6000 B inverted fluorescence microscope at 10× magnification, and analysis of the fluorescence images was performed with the LAS X software (Leica, Wetzlar, Germany). For quantitative transport experiments, cells were seeded onto polylysine-coated 96-well plates and grown to confluence over 72 h at 37 °C. Then, cells were washed three times with phosphate-buffered saline (PBS), containing 137 mM NaCl, 2.7 mM KCl, 1.5 mM, KH_2_PO_4_, and 7.3 mM Na_2_HPO_4_ (pH 7.4) followed by preincubation in sodium transport buffer (STB) containing 142.9 mM NaCl, 4.7 mM KCl, 1.2 mM MgSO_4_, 1.2 mM KH_2_PO_4_, 1.8 mM CaCl_2_, and 20 mM HEPES (pH 7.4). For transport assays under sodium-free conditions (-Na^+^), sodium chloride of the STB was substituted with equimolar concentrations of choline chloride. Transport assays were started by adding one of the NBD-BA or [^3^H]­TCA to the transport buffer, followed by incubation at 37 °C. For inhibition experiments, cells were preincubated for 5 min at 37 °C with 80 μL containing cyclosporine A or troglitazone as inhibitors at increasing concentrations. Then, transport experiments were started by adding 20 μL STB containing 125 μM of 3α-NBD-GCA (final substrate concentration: 25 μM). All transport experiments were stopped after 10 min, if not otherwise indicated, by aspirating the transport buffer and washing the cells twice with ice-cold PBS. Plates were kept cool until adding the lysis buffer, containing 1% sodium dodecyl sulfate and 1 N NaOH. Cell-associated radioactivity was quantified by liquid scintillation counting in a Packard Microplate Scintillation Counter TopCount NXT (Packard Instrument Company, Meriden, USA) and cell-based fluorescence was directly analyzed by Glomax fluorescence reader (Promega, Sunnyvale, CA, USA) at 488 nm.

### Membrane Permeability and Excitation–Emission Spectra of the 3-NBD-BA

All NBD-BA were diluted to 1 pM in STB, and extinction and emission spectra were measured from 300 to 650 nm (Spark Multimode Microplate, Tecan, Männedorf, Switzerland). LogP values were determined for all synthesized NBD-BA as well as for [^3^H]­TCA and [^3^H]­CA. The [^3^H]­CA was generously provided by Prof. Dr. Alan Hofmann, University of California (San Diego, United States). Briefly, 1 μL of the respective 3-NBD-BA (25 μM in STB), [^3^H]­TCA (1 μM in STB), or [^3^H]­CA (1 μM in STB) was added to a 1:1 mixture of 500 μL water and 500 μL 1-Octanol. After rigorous shaking and phase separation by centrifugation, 100 μL of each phase were measured directly by liquid scintillation counting or fluorescence detection at 488 nm (see above).

### Substrate Binding Site Volume Calculation for NTCP, ASBT, mNtcp, and mAsbt

The following protein sequences were used: human ASBT, UniProt Q12908; human NTCP, UniProt Q14973; mouse mAsbt, UniProt P70172; mouse mNtcp, UniProt O08705. For all proteins, AlphaFold structures were obtained from the AlphaFold database at alphafold.com.^72^ For ease of comparison, the NTCP structure was also downloaded from the AlphaFold database, even if cryo-EM structures of human NTCP are available. All structures were prepared using the protein preparation workflow from MAESTRO.^73^ Volumes of the potential substrate binding site cavities were calculated using SiteMap.^74,75^

### Data Analysis and Statistics

All data is shown as means ± SD. All transport and inhibition graphs were generated with GraphPad Prism 10 (GraphPad). Determination of *K*_*m*_ and IC_50_ values was done by nonlinear regression analysis. Statistical analysis was performed as indicated in the figure legends.

### Chemistry and Purity Statement

Solvents were purified by distillation prior to use, in the case of anhydrous solvents bottles from ACROS Organics were utilized. Commercially available chemicals were used as supplied if not stated otherwise. Syntheses in anhydrous solvents were carried out under Schlenk conditions. For purification by flash column chromatography silica gel 60 (Macherey-Nagel, Düren, Germany) was used. If not stated otherwise, ^1^H and ^13^C NMR spectra were recorded with a Bruker Avance II 400 MHz or Avance III 400 MHz (^1^H at 400 MHz and ^13^C at 101 MHz) (Bruker, Billerica, Massachusetts, USA). High resolution ESI mass spectra were recorded in methanol using a Bruker Daltonics ESImicroTOF spectrometer. HPLC analysis was performed with a Dionex Ultimate 3000 (Dionex, Sunnyvale, California, USA) and an Knauer Eurospher II C18H column (Knauer, Berlin, Germany) using the following parameters: 1 mL/min, 90% MeOH, 10% H_2_O, 0.1% AA. Detection was conducted either by UV–vis or by ELSD. Analytical data and ^1^H and ^13^C NMR spectra for all compounds synthesized within this work are included in the Supporting Information. All target compounds are confirmed to be >95% pure by HPLC analysis. The respective HPLC traces are given in the Supporting Information.

### Synthetic Procedures

#### General Procedure A for the Synthesis of BA Methyl Ester

Under nitrogen atmosphere, the respective BA (1 equiv) was dissolved in anhydrous methanol. Thionyl chloride (1.1 equiv) was added dropwise at 0 °C and the mixture was stirred for 18 h at room temperature. The solvent was removed under reduced pressure. The crude product was dissolved in ethyl acetate and washed with saturated sodium bicarbonate and brine. The organic layer was dried over MgSO_4,_ and the solvent was removed under reduced pressure.

##### Methyl-3α,7α,12α-trihydroxy-5β-cholan-24-oate (**1a**)

This compound was prepared according to general procedure A using CA (3.020 g, 7.392 mmol) to obtain compound **1a** as a white solid (2.962 g, 7.010 mmol, 95%).

HRMS (ESI): *m*/*z* = 445.2918 [M + Na]^+^ (calculated for 445.2924)

^1^H NMR (CDCl_3_, 400.1 MHz): δ [ppm] = 3.98–3.94 (m, 1H), 3.89–3.79 (m, 1H), 3.65 (s, 3H), 3.53–3.37 (m, 1H), 2.75 (s, 3H), 2.43–2.30 (m, 1H), 2.30–2.14 (m, 3H), 1.99–1.02 (m, 20H), 0.97 (d, *J* = 6.1 Hz, 3H), 0.88 (s, 3H), 0.67 (s, 3H).

^13^C NMR (CDCl_3_, 100.6 MHz): δ [ppm] = 174.94, 73.20, 72.14, 68.60, 51.64, 47.18, 46.59, 41.86, 41.60, 39.62, 35.39, 34.88, 34.76, 31.23, 31.04, 30.52, 28.35, 27.62, 26.58, 23.24, 22.61, 17.45, 12.62.

##### Methyl-3α,7α-dihydroxy-5β-cholan-24-oate (**1b**)

This compound was prepared according to general procedure A using CDCA (5.000 g, 12.736 mmol) to obtain compound **1b** as a white solid (5.141 g, 12.644 mmol, 99%).

HRMS (ESI): *m*/*z* = 429.2972 [M + Na]^+^ (calculated for 429.2975)

^1^H NMR (CDCl_3_, 400.1 MHz): δ [ppm] = 3.87–3.83 (m, 1H), 3.66 (s, 3H), 3.55–3.42 (m, 1H), 2.41–2.27 (m, 1H), 2.27–2.14 (m, 2H), 2.05–1.58 (m, 12H), 1.58–1.04 (m, 13H), 0.95–0.87 (m, 6H), 0.65 (s, 3H).

^13^C NMR (CDCl_3_, 100.6 MHz): δ [ppm] = 174.89, 72.23, 68.71, 55.92, 51.63, 50.58, 42.83, 41.60, 39.90, 39.76, 39.54, 35.51, 35.44, 35.18, 34.70, 32.98, 31.15, 31.12, 30.70, 28.28, 23.84, 22.91, 20.72, 18.40, 11.91.

##### Methyl-3α,12α-dihydroxy-5β-cholan-24-oate (**1c**)

This compound was prepared according to general procedure A using DCA (5.000 g, 12.736 mmol) to obtain compound **1c** as a white solid (4.851 g, 11.930 mmol, 94%).

HRMS (ESI): *m*/*z* = 429.2973 [M + Na]^+^ (calculated for 429.2975)

^1^H NMR (CDCl_3_, 400.1 MHz): δ [ppm] = 4.02–3.95 (m, 1H), 3.66 (s, 3H), 3.65–3.51 (m, 1H), 2.41–2.30 (m, 1H), 2.26–2.19 (m, 1H), 1.90–1.48 (m, 16H), 1.46–1.19 (m, 8H), 1.19–0.99 (m, 2H), 0.96 (d, *J* = 6.3 Hz, 3H), 0.90 (s, 3H), 0.67 (s, 3H).

^13^C NMR (CDCl_3_, 100.6 MHz): δ [ppm] = 174.84, 73.30, 71.99, 51.63, 48.41, 47.47, 46.64, 42.22, 36.54, 36.18, 35.35, 35.25, 34.26, 33.81, 31.22, 31.04, 30.60, 28.81, 27.58, 27.26, 26.26, 23.78, 23.29, 17.45, 12.88.

#### General Procedure B for the Synthesis of 3α-Mesyl-BA Methyl Ester

Under nitrogen atmosphere, triethylamine (2 equiv) was added to a solution of the respective BA methyl ester (1 equiv) in anhydrous DCM. Methanesulfonyl chloride (1 equiv) was dissolved in anhydrous DCM and was added dropwise at 0 °C. The mixture was stirred for 2 h at 0 °C and was then quenched by the addition of water. The phases were separated, and the aqueous layer was extracted three times with DCM. The combined organic layers were washed with saturated sodium bicarbonate, distilled water, and brine, and were then dried over MgSO_4_. The solvent was removed under reduced pressure and the crude product was purified by flash column chromatography.

##### Methyl-7α,12α-dihydroxy-3α-[(methylsulfonyl)­oxy]-5β-cholan-24-oate (**2a**)

This compound was prepared according to general procedure B using **1a** (1.405 g, 3.325 mmol) to obtain compound **2a** as a white foam (1.466 g, 2.928 mmol, 88%).

HRMS (ESI): *m*/*z* = 523.2695 [M + Na]^+^ (calculated for 523.2700)

^1^H NMR (CDCl_3_, 400.1 MHz): δ [ppm] = 4.57–4.46 (m, 1H), 4.02–3.96 (m, 1H), 3.90–3.81 (m, 1H), 3.66 (s, 3H), 2.98 (s, 3H), 2.60 (q, *J* = 12.9 Hz, 1H), 2.44–2.31 (m, 1H), 2.28–2.09 (m, 2H), 2.02–1.23 (m, 21H), 1.22–1.10 (m, 1H), 0.97 (d, *J* = 6.2 Hz, 3H), 0.90 (s, 3H), 0.69 (s, 3H).

^13^C NMR (CDCl_3_, 100.6 MHz): δ [ppm] = 174.85, 82.80, 72.94, 68.23, 51.67, 47.35, 46.67, 42.08, 41.56, 39.65, 39.06, 36.20, 35.27, 34.94, 34.61, 34.27, 31.17, 30.99, 28.45, 28.05, 27.55, 26.80, 23.25, 22.48, 17.48, 12.69.

##### Methyl-7α-hydroxy-3α-[(methylsulfonyl)­oxy]-5β-cholan-24-oate (**2b**)

This compound was prepared according to general procedure B using **1b** (2.500 g, 6.148 mmol) to obtain compound **2b** as a white foam (2.292 g, 4.729 mmol, 77%).

HRMS (ESI): *m*/*z* = 507.2749 [M + Na]^+^ (calculated for 507.2751)

^1^H NMR (CDCl_3_, 400.1 MHz): δ [ppm] = 4.57–4.47 (m, 1H), 3.89–3.79 (m, 1H), 3.66 (s, 3H), 2.98 (s, 3H), 2.71–2.44 (m, 1H), 2.44–2.30 (m, 1H), 2.30–2.17 (m, 1H), 2.00–1.76 (m, 8H), 1.71–1.56 (m, 2H), 1.54–1.23 (m, 11H), 1.22–1.06 (m, 3H), 0.97–0.87 (m, 6H), 0.65 (s, 3H).

^13^C NMR (CDCl_3_, 100.6 MHz): δ [ppm] = 174.86, 82.89, 68.41, 55.91, 51.64, 50.50, 42.82, 41.60, 39.65, 39.49, 39.04, 36.29, 35.49, 35.07, 35.00, 34.33, 32.91, 31.14, 31.10, 28.26, 28.14, 23.81, 22.69, 20.70, 18.39, 11.90.

##### Methyl-12α-hydroxy-3α-[(methylsulfonyl)­oxy]-5β-cholan-24-oate (**2c**)

This compound was prepared according to general procedure B using **1c** (2.500 g, 6.148 mmol) to obtain compound **2c** as a white foam (2.469 g, 5.094 mmol, 83%).

HRMS (ESI): *m*/*z* = 507.2749 [M + Na]^+^ (calculated for 507.2751)

^1^H NMR (CDCl_3_, 400.1 MHz): δ [ppm] = 4.72–4.59 (m, 1H), 4.01–3.95 (m, 1H), 3.66 (s, 3H), 2.99 (s, 3H), 2.43–2.33 (m, 1H), 2.27–2.18 (m, 1H), 2.13–2.02 (m, 1H), 1.93–1.52 (m, 12H), 1.52–1.21 (m, 10H), 1.14–1.02 (m, 2H), 0.97 (d, *J* = 6.3 Hz, 3H), 0.92 (s, 3H), 0.68 (s, 3H).

^13^C NMR (CDCl_3_, 100.6 MHz): δ [ppm] = 174.79, 82.80, 73.16, 51.65, 48.34, 47.55, 46.65, 42.26, 39.06, 36.07, 35.19, 35.00, 34.07, 33.77, 33.45, 31.20, 31.02, 28.79, 27.87, 27.55, 26.95, 26.07, 23.71, 23.07, 17.50, 12.89.

##### Synthesis of Methyl-7α,12α-dihydroxy-3β-[(methylsulfonyl)­oxy]-5β-cholan-24-oate (**7**)

Under nitrogen atmosphere, methyl-3α,7α,12α-trihydroxy-5β-cholan-24-oate **1a** (2.945 g, 6.967 mmol, 1 equiv) and triphenylphosphine (5.483 g, 20.906 mmol, 3 equiv) were dissolved in 40 mL anhydrous THF. Methanesulfonic acid (0.90 mL, 13.86 mmol, 2 equiv) was added and the mixture was heated to 50 °C. Then 1.9 M DIAD (11.00 mL, 20.91 mmol, 3 equiv) was added over a period of 15 min, and the mixture was stirred for 18 h at 50 °C. The solvent was removed under reduced pressure and the crude product was purified by flash column chromatography (ethyl acetate/cyclohexane 3:1) to obtain the product as a white foam.

HRMS (ESI): *m*/*z* = 523.2705 [M + Na]^+^ (calculated for 523.2700)

^1^H NMR (CDCl_3_, 400.1 MHz): δ [ppm] = 5.01–4.91 (m, 1H), 3.98 (t, *J* = 3.0 Hz, 1H), 3.89–3.84 (m, 1H), 3.66 (s, 3H), 2.98 (s, 3H), 2.42–2.32 (m, 1H), 2.26–2.18 (m, 1H), 2.16–2.06 (m, 1H), 1.97–1.66 (m, 12H), 1.66–1.49 (m, 5H), 1.47–1.25 (m, 5H), 1.18–1.07 (m, 1H), 1.01–0.90 (m, 6H), 0.69 (s, 3H).

##### Synthesis of Methyl-7α,12α-dihydroxy-3β-[(trifluoroacetyl)­oxy]-5β-cholan-24-oate (**8**)

Under nitrogen atmosphere, methyl-3α,7α,12α-trihydroxy-5β-cholan-24-oate **1a** (5.459 g, 12.917 mmol, 1 equiv) and triphenylphosphine (10.174 g, 38.790 mmol, 3 equiv) were dissolved in 60 mL anhydrous THF and TFA (2.00 mL, 26.14 mmol, 2 equiv) was added. At 0 °C and over a period of 10 min DIAD (7.61 mL, 38.75 mmol, 3 equiv) was added. Then, the mixture was stirred for 18 h at 50 °C. The solvent was removed under reduced pressure and the crude product was purified by flash column chromatography (ethyl acetate/cyclohexane 1:2). The product was obtained containing diisopropyl hydrazodicarboxylate and was used in the next step without further purification.

HRMS (ESI): *m*/*z* = 541.2749 [M + Na]^+^ (calculated for 541.2747)

^1^H NMR (CDCl_3_, 400.1 MHz): δ [ppm] = 5.26–5.20 (m, 1H), 4.03–3.97 (m, 1H), 3.91–3.84 (m, 1H), 3.66 (s, 3H), 2.72–2.57 (m, 1H), 2.42–2.31 (m, 1H), 2.28–2.10 (m, 2H), 2.02–1.78 (m, 4H), 1.74–1.65 (m, 9H), 1.62–1.53 (m, 4H), 1.47–1.27 (m, 5H), 0.97 (d, *J* = 9.5 Hz, 6H), 0.70 (s, 3H).

#### General Procedure C for the Synthesis of 3-Azido-BA Methyl Ester

Under nitrogen atmosphere, the respective 3-mesyl-BA methyl ester (1 equiv) or 3β-trifluoroacetate-CA methyl ester (1 equiv) and sodium azide (5 equiv) were dissolved in anhydrous DMF. The mixture was stirred for 48–72 h at 80 °C. Then, water and ethyl acetate were added to the mixture and the phases were separated. The aqueous layer was extracted three times with ethyl acetate. The combined organic layers were washed three times with brine and dried over MgSO_4_. The solvent was removed under reduced pressure and the crude product was purified by flash column chromatography.

##### Methyl-3β-azido-7α,12α-dihydroxy-5β-cholan-24-oate (**3a**)

This compound was prepared according to general procedure C using **2a** (0.577 g, 1.152 mmol) to obtain compound **3a** as a white solid (0.399 g, 0.891 mmol, 77%).

HRMS (ESI): *m*/*z* = 470.2991 [M + Na]^+^ (calculated for 470.2989)

^1^H NMR (CDCl_3_, 400.1 MHz): δ [ppm] = 4.00–3.95 (m, 1H), 3.92–3.88 (m, 1H), 3.88–3.83 (m, 1H), 3.66 (s, 3H), 2.58–2.48 (m, 1H), 2.40–2.31 (m, 1H), 2.27–2.08 (m, 2H), 2.02–1.26 (m, 21H), 1.19–1.10 (m, 1H), 0.97 (d, *J* = 6.3 Hz, 3H), 0.92 (s, 3H), 0.69 (s, 3H).

^13^C NMR (CDCl_3_, 100.6 MHz): δ [ppm] = 174.83, 73.09, 68.54, 58.85, 51.67, 47.41, 46.71, 42.13, 39.61, 36.90, 35.29, 35.21, 34.20, 33.17, 31.20, 30.99, 30.63, 28.64, 27.58, 26.38, 24.70, 23.31, 23.03, 17.48, 12.68.

##### Methyl-3β-azido-7α-hydroxy-5β-cholan-24-oate (**3b**)

This compound was prepared according to general procedure C using **2b** (2.269 g, 4.681 mmol) to obtain compound **3b** as a white foam (1.413 g, 3.274 mmol, 70%).

HRMS (ESI): *m*/*z* = 454.3044 [M + Na]^+^ (calculated for 454.3040)

^1^H NMR (CDCl_3_, 400.1 MHz): δ [ppm] = 3.93–3.87 (m, 1H), 3.87–3.82 (m, 1H), 3.66 (s, 3H), 2.56–2.45 (m, 1H), 2.42–2.29 (m, 1H), 2.29–2.17 (m, 1H), 2.06–1.70 (m, 5H), 1.70–1.24 (m, 17H), 1.23–1.06 (m, 3H), 0.97–0.87 (m, 6H), 0.66 (s, 3H).

^13^C-NMR (CDCl_3_, 100.6 MHz): δ [ppm] = 174.88, 68.75, 58.92, 55.96, 51.64, 50.63, 42.88, 39.77, 39.49, 36.96, 35.61, 35.52, 34.23, 33.29, 32.61, 31.17, 31.13, 30.76, 28.29, 24.81, 23.87, 23.30, 20.94, 18.41, 11.92.

##### Methyl-3β-azido-12α-hydroxy-5β-cholan-24-oate (**3c**)

This compound was prepared according to general procedure C using **2c** (1.891 g, 3.943 mmol) to obtain compound **3c** as a white foam (1.297 g, 3.005 mmol, 76%).

HRMS (ESI): *m*/*z* = 454.3040 [M + Na]^+^ (calculated for 454.3040)

^1^H NMR (CDCl_3_, 400.1 MHz): δ [ppm] = 4.02–3.97 (m, 1H), 3.97–3.91 (m, 1H), 3.66 (s, 3H), 2.43–2.31 (m, 1H), 2.28–2.17 (m, 1H), 2.02 (ddd, *J* = 14.4, 13.2, 3.5 Hz, 1H), 1.91–1.75 (m, 3H), 1.72–1.19 (m, 20H), 1.16–1.01 (m, 2H), 0.96 (d, *J* = 6.3 Hz, 2H), 0.94 (s, 3H), 0.68 (s, 3H).

^13^C NMR (CDCl_3_, 100.6 MHz): δ [ppm] = 174.80, 73.33, 58.87, 51.66, 48.50, 47.59, 46.67, 37.44, 35.98, 35.19, 34.63, 33.43, 31.20, 31.03, 30.67, 30.27, 28.93, 27.57, 26.59, 26.11, 24.75, 23.71, 23.67, 17.51, 12.89.

##### Methyl-3α-azido-7α,12α-dihydroxy-5β-cholan-24-oate (**3d**)

This compound was prepared according to general procedure C using **7** to obtain compound **3d** as a white foam (0.405 g, 0.906 mmol, 13% over 2 steps).

This compound was prepared according to general procedure C using **8** to obtain compound **3d** as a white foam (0.764 g, 1.707 mmol, 13% over 2 steps).

HRMS (ESI): *m*/*z* = 470.2984 [M + Na]^+^ (calculated for 470.2989)

^1^H NMR (CDCl_3_, 400.1 MHz): δ [ppm] = 4.01–3.97 (m, 1H), 3.89–3.81 (m, 1H), 3.78–3.71 (m, 1H), 3.66 (s, 3H), 3.19–3.10 (m, 1H), 2.40–2.12 (m, 4H), 2.00–1.79 (m, 8H), 1.76–1.49 (m, 8H), 1.45–1.31 (m, 4H), 1.21–1.09 (m, 1H), 0.97 (d, *J* = 6.2 Hz, 3H), 0.90 (s, 3H), 0.68 (s, 3H).

^13^C NMR (CDCl_3_, 100.6 MHz): δ [ppm] = 174.93, 73.10, 68.34, 61.47, 51.67, 47.36, 46.69, 42.08, 41.96, 39.60, 35.52, 35.37, 34.89, 34.64, 31.18, 30.97, 28.41, 27.61, 26.95, 26.76, 25.74, 23.31, 22.75, 17.46, 12.65

#### General Procedure D for the Synthesis of 3-Amino-BA Methyl Ester

To a solution of the respective 3-azido-BA methyl ester (1 equiv) in THF and distilled water (0.2 mL/mmol), triphenylphosphine (1.5 equiv) was added and the mixture was stirred for 18 h at 50 °C. The organic solvent was removed under reduced pressure and the crude product was purified by flash column chromatography.

##### Methyl-3β-amino-7α,12α-dihydroxy-5β-cholan-24-oate (**4a**)

This compound was prepared according to general procedure D using **3a** (0.379 g, 0.847 mmol) to obtain compound **4d** as a white foam (0.327 g, 0.776 mmol, 92%).

HRMS (ESI): *m*/*z* = 422.3267 [M + H]^+^ (calculated for 422.3265)

^1^H NMR (CDCl_3_, 400.1 MHz): δ [ppm] = 4.03–3.92 (m, 1H), 3.88–3.78 (m, 1H), 3.65 (s, 3H), 3.30–3.21 (m, 1H), 2.59–2.46 (m, 1H), 2.44–2.31 (m, 1H), 2.29–2.09 (m, 3H), 1.99–1.62 (m, 10H), 1.58–1.25 (m, 12H), 1.21–1.05 (m, 1H), 0.97 (d, *J* = 6.1 Hz, 3H), 0.93 (s, 3H), 0.68 (s, 3H).

^13^C NMR (CDCl_3_, 100.6 MHz): δ [ppm] = 174.88, 77.36, 73.11, 68.53, 53.13, 51.64, 47.34, 46.65, 42.04, 39.58, 35.97, 35.54, 35.36, 34.62, 31.27, 31.03, 29.97, 28.66, 27.62, 26.21, 23.39, 23.06, 17.48, 12.67, 8.28.

##### Methyl-3β-amino-7α-hydroxy-5β-cholan-24-oate (**4b**)

This compound was prepared according to general procedure D using **3b** (1.397 g, 3.237 mmol) to obtain compound **4b** as a white foam (1.207 g, 2.976 mmol, 92%).

HRMS (ESI): *m*/*z* = 406.3315 [M + H]^+^ (calculated for 406.3316)

^1^H NMR (CDCl_3_, 400.1 MHz): δ [ppm] = 3.87–3.66 (m, 1H), 3.59 (s, 3H), 3.29–3.15 (m, 1H), 2.52–2.38 (m, 1H), 2.32–2.21 (m, 1H), 2.20–2.08 (m, 1H), 2.03–1.50 (m, 9H), 1.46–1.17 (m, 14H), 1.17–0.96 (m, 3H), 0.89 (s, 3H), 0.85 (d, *J* = 6.3 Hz, 3H), 0.59 (s, 3H).

^13^C NMR (CDCl_3_, 100.6 MHz): δ [ppm] = 174.89, 68.76, 55.92, 51.63, 50.62, 46.79, 42.85, 39.77, 39.44, 36.04, 35.92, 35.90, 35.52, 34.57, 32.41, 31.16, 31.14, 29.89, 28.29, 27.23, 23.88, 23.29, 20.94, 18.40, 11.91.

##### Methyl-3β-amino-12α-hydroxy-5β-cholan-24-oate (**4c**)

This compound was prepared according to general procedure D using **3c** (1.589 g, 3.681 mmol) to obtain compound **4c** as a white foam (1.471 g, 3.626 mmol, 98%).

HRMS (ESI): *m*/*z* = 406.3318 [M + H]^+^ (calculated for 406.3316)

^1^H NMR (CDCl_3_, 400.1 MHz): δ [ppm] = 3.97 (t, *J* = 3.0 Hz, 1H), 3.65 (s, 3H), 3.31–3.11 (m, 1H), 2.46–2.27 (m, 1H), 2.26–2.17 (m, 1H), 2.12–2.00 (m, 1H), 1.96–1.76 (m, 6H), 1.72–1.44 (m, 8H), 1.44–1.20 (m, 8H), 1.17–1.03 (m, 4H), 0.99–0.88 (m, 6H), 0.67 (s, 3H).

^13^C NMR (CDCl_3_, 100.6 MHz): δ [ppm] = 174.83, 73.32, 51.63, 48.54, 47.49, 46.65, 46.47, 36.50, 35.96, 35.20, 34.97, 33.46, 33.07, 31.19, 31.04, 29.85, 29.00, 27.76, 27.57, 26.89, 26.12, 23.86, 23.73, 17.47, 12.88.

##### Methyl-3α-amino-7α,12α-dihydroxy-5β-cholan-24-oate (**4d**)

This compound was prepared according to general procedure D using **3d** (0.348 g, 0.858 mmol) to obtain compound **4d** as a white foam (0.349 g, 0.828 mmol, 96%).

HRMS (ESI): *m*/*z* = 422.3268 [M + H]^+^ (calculated for 422.3265)

^1^H NMR (CDCl_3_, 400.1 MHz): δ [ppm] = 3.94 (s, 1H), 3.81 (s, 1H), 3.65 (s, 3H), 2.63 (s, 1H), 2.42–2.29 (m, 1H), 2.29–2.13 (m, 3H), 1.99–1.03 (m, 23H), 1.14–1.05 (m, 1H), 0.97 (d, *J* = 6.1 Hz, 3H), 0.89 (s, 3H), 0.66 (s, 3H).

^13^C NMR (CDCl_3_, 100.6 MHz): δ [ppm] = 174.87, 72.99, 68.28, 53.20, 51.75, 51.61, 47.14, 46.55, 42.07, 41.97, 39.70, 35.99, 35.38, 34.85, 34.76, 31.22, 31.07, 28.44, 27.62, 26.73, 23.33, 22.73, 17.41, 12.64, 8.29.

#### General Procedure E for the Synthesis of 3-NBD-BA Methyl Ester

To a solution of the respective 3-amino-BA methyl ester (1 equiv) in methanol, 4-chloro-7-nitrobenzo-2-oxa-1,3-diazol (1.6 equiv) and sodium bicarbonate (2.2 equiv) were added, and the mixture was stirred for 18 h at 50 °C. The solvent was removed under reduced pressure and the crude product was purified by flash column chromatography.

##### Methyl-7α,12α-dihydroxy-3β-[(7-nitro-2,1,3-benzoxadiazol-4-yl)­amino]-5β-cholan-24-oate (**5a**)

This compound was prepared according to general procedure E using **4a** (0.748 g, 1.774 mmol) to obtain compound **5a** as an orange solid (0.769 g, 1.315 mmol, 74%).

HRMS (ESI): *m*/*z* = 607.3099 [M + Na]^+^ (calculated for 607.3102)

^1^H NMR (CDCl_3_, 400.1 MHz): δ [ppm] = 8.47 (d, *J* = 8.7 Hz, 1H), 6.40 (d, *J* = 7.1 Hz, 1H), 6.17 (d, *J* = 8.7 Hz, 1H), 4.12–3.99 (m, 1H), 3.96–3.86 (m, 1H), 3.67 (s, 3H), 2.87–2.72 (m, 1H), 2.43–2.33 (m, 1H), 2.30–2.19 (m, 2H), 2.09–2.00 (m, 1H), 1.99–1.12 (m, 22H), 1.02–0.96 (m, 6H), 0.72 (s, 3H).

^13^C NMR (CDCl_3_, 100.6 MHz): δ [ppm] = 174.78, 144.62, 144.10, 143.11, 136.78, 123.75, 99.11, 72.97, 68.38, 51.69, 49.85, 47.50, 46.76, 42.08, 39.68, 37.39, 35.35, 35.25, 34.03, 32.63, 31.21, 31.06, 31.00, 28.72, 27.58, 26.40, 24.02, 23.30, 23.22, 17.53, 12.72.

##### Methyl-7α-hydroxy-3β-[(7-nitro-2,1,3-benzoxadiazol-4-yl)­amino]-5β-cholan-24-oate (**5b**)

This compound was prepared according to general procedure E using **4b** (1.187 g, 2.962 mmol) to obtain compound **5b** as an orange solid (1.313 g, 2.309 mmol, 79%).

HRMS (ESI): *m*/*z* = 591.3153 [M + Na]^+^ (calculated for 591.3151)

^1^H NMR (CDCl_3_, 700.1 MHz): δ [ppm] = 8.47 (d, *J* = 8.7 Hz, 1H), 6.40 (d, *J* = 7.2 Hz, 1H), 6.17 (d, *J* = 8.9 Hz, 1H), 4.11–3.99 (m, 1H), 3.92–3.83 (m, 1H), 3.66 (s, 3H), 2.85–2.73 (m, 1H), 2.41–2.31 (m, 1H), 2.30–2.15 (m, 1H), 2.09–2.04 (m, 1H), 2.03–1.97 (m, 1H), 1.95–1.72 (m, 7H), 1.66–1.59 (m, 2H), 1.57–1.51 (m, 1H), 1.51–1.30 (m, 8H), 1.28–1.14 (m, 4H), 1.01 (s, 3H), 0.93 (d, *J* = 6.5 Hz, 3H), 0.68 (s, 3H).

^13^C NMR (CDCl_3_, 176.6 MHz): δ [ppm] = 174.85, 144.62, 144.11, 143.13, 136.74, 123.74, 99.03, 68.60, 55.97, 51.66, 50.53, 49.86, 42.88, 39.69, 39.49, 37.43, 35.75, 35.49, 34.06, 32.70, 32.49, 31.15, 31.14, 31.10, 28.26, 24.13, 23.86, 23.47, 20.99, 18.41, 11.93.

##### Methyl-12α-hydroxy-3β-[(7-nitro-2,1,3-benzoxadiazol-4-yl)­amino]-5β-cholan-24-oate (**5c**)

This compound was prepared according to general procedure E using **4c** (1.443 g, 3.558 mmol) to obtain compound **5c** as an orange solid containing NBDOH. The product was used in the next step without further purification.

HRMS (ESI): *m*/*z* = 591.3147 [M + Na]^+^ (calculated for 591.3153)

^1^H NMR (CDCl_3_, 400.1 MHz): δ [ppm] = 8.47 (d, *J* = 8.7 Hz, 1H), 6.44 (d, *J* = 7.3 Hz, 1H), 6.16 (d, *J* = 8.7 Hz, 1H), 4.15–4.07 (m, 1H), 4.04–3.98 (m, 1H), 3.66 (s, 3H), 2.44–2.18 (m, 3H), 2.02–1.07 (m, 24H), 1.01 (s, 3H), 0.97 (d, *J* = 6.3 Hz, 3H), 0.70 (s, 3H).

##### Methyl-7α,12α-dihydroxy-3α-[(7-nitro-2,1,3-benzoxadiazol-4-yl)­amino]-5β-cholan-24-oate (**5d**)

This compound was prepared according to general procedure E using **4d** (0.697 g, 1.653 mmol) to obtain compound **5d** as an orange solid (0.791 g, 1.353 mmol, 82%).

HRMS (ESI): *m*/*z* = 607.3098 [M + Na]^+^ (calculated for 607.3102)

^1^H NMR (CDCl_3_, 400.1 MHz): δ [ppm] = 8.43 (d, *J* = 8.7 Hz, 1H), 6.42 (d, *J* = 8.0 Hz, 1H), 6.13 (d, J = 8.8 Hz, 1H), 4.13–3.97 (m, 1H), 3.94–3.88 (m, 1H), 3.66 (s, 3H), 3.50 (s, 1H), 2.50 (q, *J* = 12.9 Hz, 1H), 2.41–2.32 (m, 1H), 2.30–2.18 (m, 2H), 2.08–1.97 (m, 1H), 1.95–1.78 (m, 8H), 1.74–1.50 (m, 8H), 1.47–1.27 (m, 3H), 1.22–1.08 (m, 2H), 1.01–0.94 (m, 6H), 0.72 (s, 3H).

^13^C NMR (CDCl_3_, 100.6 MHz): δ [ppm] = 174.88, 144.49, 144.16, 143.32, 136.80, 123.32, 98.61, 73.03, 68.37, 54.39, 51.71, 47.46, 46.74, 42.12, 41.96, 39.70, 35.86, 35.56, 35.28, 34.95, 34.35, 31.18, 30.95, 28.44, 27.59, 27.12, 26.97, 23.28, 22.81, 17.51, 12.73.

#### General Procedure F for the Synthesis of 3-NBD-BA

To a solution of the respective 3-NBD-BA methyl ester (1 equiv) in methanol, a 2 N solution of lithium hydroxide (10 equiv) was added and the mixture was stirred for 3 h at 40 °C. The organic solvent was removed under reduced pressure and the residue was dissolved in ethyl acetate and 2 N HCl. The phases were separated, and the aqueous layer was extracted three times with ethyl acetate. The combined organic layers were dried over MgSO_4,_ and the solvent was removed under reduced pressure. The crude product was purified by flash column chromatography.

##### 7α,12α-Dihydroxy-3β-[(7-nitro-2,1,3-benzoxadiazol-4-yl)­amino]-5β-cholan-24-oate (**6a**)

This compound was prepared according to general procedure F using **5a** (0.127 g, 0.217 mmol) to obtain compound **6a** as an orange solid (0.096 g, 0.168 mmol, 78%).

HRMS (ESI): *m*/*z* = 593.2943 [M + Na]^+^ (calculated for 593.2945)

^1^H NMR (DMSO-*d*_6_, 400.1 MHz): δ [ppm] = 11.93 (s, 1H), 9.33–8.85 (m, 1H), 8.62–8.35 (m, 1H), 6.94–6.28 (m, 1H), 4.15 (t, *J* = 2.8 Hz, 1H), 4.13–3.99 (m, 1H), 3.79 (q, *J* = 3.1 Hz, 1H), 3.63 (t, *J* = 3.2 Hz, 1H), 3.17 (d, *J* = 4.9 Hz, 1H), 2.82–2.65 (m, 1H), 2.27–2.08 (m, 3H), 2.04–1.95 (m, 1H), 1.90–1.54 (m, 9H), 1.51–1.13 (m, 10H), 0.95–0.85 (m, 6H), 0.59 (s, 3H).

^13^C NMR (DMSO-*d*_6_, 100.6 MHz): δ [ppm] = 174.99, 144.49, 144.23, 137.70, 120.72, 100.21, 88.06, 71.02, 66.26, 49.91, 46.11, 45.82, 41.38, 40.15, 39.41, 36.39, 35.05, 34.56, 34.15, 31.93, 30.83, 30.80, 28.68, 27.27, 26.16, 23.05, 22.78, 22.62, 16.96, 12.34.

##### 7α-Hydroxy-3β-[(7-nitro-2,1,3-benzoxadiazol-4-yl)­amino]-5β-cholan-24-oate (**6b**)

This compound was prepared according to general procedure F using **5b** (1.291 g, 2.270 mmol) to obtain compound **6b** as an orange solid (0.999 g, 1.801 mmol, 79%).

HRMS (ESI): *m*/*z* = 577.2994 [M + Na]^+^ (calculated for 577.2996)

^1^H NMR (CDCl_3_, 400.1 MHz): δ [ppm] = 8.48 (d, *J* = 8.7 Hz, 1H), 6.40 (d, *J* = 7.3 Hz, 1H), 6.17 (d, *J* = 8.8 Hz, 1H), 4.07–4.03 (m, 1H), 3.96–3.81 (m, 1H), 2.92–2.70 (m, 1H), 2.46–2.35 (m, 1H), 2.34–2.21 (m, 1H), 2.14–1.69 (m, 9H), 1.70–1.10 (m, 16H), 1.02 (s, 3H), 0.95 (d, *J* = 6.5 Hz, 3H), 0.69 (s, 3H).

^13^C NMR (CDCl_3_, 100.6 MHz): δ [ppm] = 179.45, 144.63, 144.12, 143.11, 136.73, 123.79, 99.04, 68.66, 55.96, 50.54, 49.85, 42.91, 39.70, 39.48, 37.43, 35.76, 35.47, 34.07, 32.72, 32.51, 31.15, 30.97, 30.88, 28.27, 24.14, 23.87, 23.48, 21.00, 18.39, 11.95.

##### 12α-Hydroxy-3β-[(7-nitro-2,1,3-benzoxadiazol-4-yl)­amino]-5β-cholan-24-oate (**6c**)

This compound was prepared according to general procedure F using **5c** to obtain compound **6c** as an orange solid (0.599 g, 1.080 mmol, 30% over 2 steps).

HRMS (ESI): *m*/*z* = 577.2992 [M + Na]^+^ (calculated for 577.2996)

^1^H NMR (CDCl_3_/MeOD, 400.1 MHz): δ [ppm] = 8.28 (d, *J* = 8.8 Hz, 1H), 6.05 (d, *J* = 8.8 Hz, 1H), 3.98–3.82 (m, 1H), 3.82–3.65 (m, 1H), 2.20–1.91 (m, 3H), 1.82 (d, *J* = 4.5 Hz, 1H), 1.74–1.59 (m, 4H), 1.61–1.49 (m, 3H), 1.49–1.33 (m, 6H), 1.34–1.13 (m, 5H), 1.09–0.86 (m, 7H), 0.84–0.68 (m, 6H), 0.57–0.39 (s, 3H).

^13^C NMR (CDCl_3_/MeOD, 100.6 MHz): δ [ppm] = 176.92, 175.33, 136.93, 99.19, 90.36, 72.52, 60.40, 47.86, 46.76, 46.72, 46.20, 37.38, 35.54, 35.07, 34.34, 32.78, 30.81, 30.68, 30.64, 29.35, 28.59, 27.26, 26.29, 25.61, 23.40, 23.18, 20.44, 16.63, 13.59, 12.34.

##### 7α,12α-Dihydroxy-3α-[(7-nitro-2,1,3-benzoxadiazol-4-yl)­amino]-5β-cholan-24-oate (**6d**)

This compound was prepared according to general procedure F using **5d** (0.584 g, 0.999 mmol) to obtain compound **6d** as an orange solid (0.445 g, 0.780 mmol, 78%).

HRMS (ESI): *m*/*z* = 593.2948 [M + Na]^+^ (calculated for 593.2945)

^1^H NMR (DMSO-*d*_6_, 400.1 MHz): δ [ppm] = 11.93 (s, 1H), 9.65 (s, 1H), 8.46 (d, *J* = 9.0 Hz, 1H), 6.43 (d, *J* = 9.1 Hz, 1H), 4.15 (d, *J* = 3.4 Hz, 1H), 4.12–4.02 (m, 1H), 3.81 (d, *J* = 3.4 Hz, 1H), 3.61 (d, *J* = 18.3 Hz, 1H), 3.17 (s, 1H), 2.69–2.53 (m, 1H), 2.37–2.17 (m, 2H), 2.16–1.95 (m, 2H), 1.94–0.98 (m, 19H), 1.00–0.75 (m, 6H), 0.60 (s, 3H).

^13^C NMR (DMSO-*d*_6_, 100.6 MHz): δ [ppm] = 173.82, 144.48, 144.47, 137.95, 119.93, 99.23, 70.85, 66.07, 54.30, 50.88, 50.66, 46.06, 45.78, 41.69, 41.41, 35.46, 35.08, 34.67, 34.49, 30.70, 30.51, 28.64, 27.76, 27.29, 26.33, 22.78, 16.89, 12.33.

##### Synthesis of Tetrabutylammonium-2-[(*tert*-butoxycarbonyl)­amino]­ethanesulfonic Acid (**10**)

Taurine (0.252 g, 2.014 mmol, 1 equiv) and 40% aqueous tetrabutylammonium hydroxide (1.300 g, 2.004 mmol, 1 equiv) were dissolved in 8 mL distilled water. Boc anhydride (0.439 g, 2.011 mmol, 1 equiv) in 10 mL acetone was added dropwise. The mixture was stirred for 18 h at room temperature. Then, the organic solvent was removed under reduced pressure. The aqueous residue was extracted three times with 20 mL DCM and the combined organic layers were dried over MgSO_4_. The solvent was removed under reduced pressure to obtain the product as a pale-yellow gel (0.897 g, 1.922 mmol, 96%).

HRMS (ESI): *m*/*z* = 224.0603 [M]^−^ (calculated for 224.0598)

HRMS (ESI): *m*/*z* = 242.2846 [M]^+^ (calculated for 242.2842)

^1^H NMR (CDCl_3_, 400.1 MHz): δ [ppm] = 3.95–3.44 (m, 2H), 3.40–3.08 (m, 8H), 3.18–2.53 (m, 2H), 1.84–1.56 (m, 8H), 1.50–1.40 (m, 8H), 1.40 (s, 9H), 1.00 (t, *J* = 7.3 Hz, 12H).

^13^C NMR (CDCl_3_, 100.6 MHz): δ [ppm] = 156.18, 78.62, 58.99, 50.94, 37.16, 28.59, 24.17, 19.87, 13.80.

##### Synthesis of 2,2,2-Trifluorethyl-2-[[1,1-dimethylethoxy)­carbonyl]­amino]­ethane-sulfonate (**11**)

Under nitrogen atmosphere, tetrabutylammonium-2-[(tert-butoxycarbonyl)­amino]­ethane-sulfonic acid **10** (5.044 g, 10.807 mmol, 1 equiv) was dissolved in 30 mL anhydrous DMF. Oxalyl chloride (1.10 mL, 12.86 mmol, 1.2 equiv) in 10 mL anhydrous DCM was added dropwise at 0 °C and the mixture was stirred for 1 h at 0 °C (mixture A). In a further flask, triethylamine (2.23 mL, 16.07 mmol, 1.5 equiv) was dissolved in 20 mL DCM under nitrogen atmosphere. Trifluoroethanol (0.93 mL, 12.86 mmol, 1.2 equiv) was added dropwise at 0 °C and the mixture was stirred for 1 h at 0 °C (mixture B). Mixture A was added to mixture B dropwise at 0 °C and the resulting mixture was stirred for 18 h at room temperature. Then, 30 mL distilled water were added, and the phases were separated. The aqueous layer was extracted three times with 20 mL diethyl ether. The combined organic layers were washed with 30 mL distilled water and then dried over MgSO_4_. The solvent was removed under reduced pressure and the crude product was purified by flash column chromatography (ethyl acetate/cyclohexane 1:3) to obtain the product as a white solid (1.805 g, 5.874 mmol, 54%).

HRMS (ESI): *m*/*z* = 330.0594 [M + Na]^+^ (calculated for 330.0593)

^1^H NMR (CDCl_3_, 400.1 MHz): δ [ppm] = 5.07 (s, 1H), 4.52 (q, *J* = 7.9 Hz, 2H), 3.73–3.58 (m, 2H), 3.55–3.35 (m, 2H), 1.44 (s, 9H).

^13^C NMR (CDCl_3_, 100.6 MHz): δ [ppm] = 155.69, 122.06 (q, *J* = 277.8 Hz), 80.48, 64.00 (q, *J* = 38.3 Hz), 51.50, 35.47, 28.40.

##### Synthesis of 2,2,2-Trifluorethyl-2-aminoethanesulfonate (**12**)

TFA (1.89 mL, 24.47 mmol, 10 equiv) was added to a solution of 2,2,2-trifluorethyl-2-[[1,1-dimethylethoxy)­carbonyl]­amino]­ethanesulfonate **11** (0.752 g, 2.447 mmol, 1 equiv) in 50 mL DCM. The mixture was stirred for 4 h at room temperature. Then, the solvent was removed under reduced pressure and the residue was dissolved in 20 mL ethyl acetate. The organic layer was washed three times with 20 mL 20 w% sodium hydroxide and two times with brine. The organic layer was dried over MgSO_4,_ and the solvent was removed under reduced pressure to obtain the product as a colorless gel (0.465 g, 2.245 mmol, 92%).

HRMS (ESI): *m*/*z* = 208.0250 [M + H]^+^ (calculated for 208.0250)

^1^H NMR (CDCl_3_, 400.1 MHz): δ [ppm] = 4.55 (q, *J* = 7.9 Hz, 2H), 3.40–3.33 (m, 2H), 3.31–3.16 (m, 2H), 1.43 (s, 2H).

^13^C NMR (CDCl_3_, 100.6 MHz): δ [ppm] = 122.16 (q, *J* = 277.5 Hz), 63.92 (q, *J* = 38.2 Hz), 54.95, 36.92.

#### General Procedure G for the Synthesis of Trifluoroethanol-protected 3-NBD-TBA

Under nitrogen atmosphere, the respective 3-NBD-BA (1 equiv) was dissolved in anhydrous DMF. Triethylamine (3.5 equiv), TBTU (1.5 equiv), and HOBt·H_2_O (1.5 equiv) were added, and the mixture was stirred for 45 min at room temperature. Then 2,2,2-trifluorethyl-2-aminoethanesulfonate (1 equiv) in DMF was added and the mixture was stirred for 18 h at room temperature. Distilled water was added, and the aqueous layer was extracted three times with ethyl acetate. The combined organic layers were washed with saturated sodium bicarbonate, potassium bisulfate, sodium bicarbonate, and brine. The organic layer was dried over MgSO_4,_ and the solvent was removed under reduced pressure. The crude product was purified by flash column chromatography.

##### 7α,12α-Dihydroxy-3β-[(7-nitro-2,1,3-benzoxadiazol-4-yl)­amino]-5β-oxocholane-24-yl]­amino] Ethane Trifluoroethanesulfonic Acid Ester (**13a**)

This compound was prepared according to general procedure G using **6a** (0.200 g, 0.350 mmol) to obtain compound **13a** as an orange solid (0.245 g, 0.322 mmol, 92%).

HRMS (ESI): *m*/*z* = 782.3015 [M + Na]^+^ (calculated for 782.3017)

^1^H NMR (CDCl_3_, 400.1 MHz): δ [ppm] = 8.48 (d, *J* = 8.6 Hz, 1H), 6.40 (d, *J* = 7.1 Hz, 1H), 6.24–6.19 (m, 1H), 6.18 (d, *J* = 8.7 Hz, 1H), 4.54 (q, *J* = 7.9 Hz, 2H), 4.11–4.03 (m, 1H), 4.03–4.00 (m, 1H), 3.95–3.87 (m, 1H), 3.77 (q, *J* = 5.8 Hz, 2H), 3.52–3.44 (m, 2H), 2.86–2.73 (m, 1H), 2.34–2.21 (m, 2H), 2.18–1.10 (m, 23H), 1.03–0.95 (m, 6H), 0.72 (s, 3H).

^13^C NMR (CDCl_3_, 100.6 MHz): δ [ppm] = 174.23, 144.63, 144.12, 143.13, 136.81, 123.76, 122.06 (q, *J* = 277.7 Hz), 99.13, 77.37, 73.00, 68.37, 64.15 (q, *J* = 38.2 Hz), 51.05, 49.84, 47.21, 46.76, 42.12, 39.66, 37.40, 35.36, 35.33, 34.19, 34.02, 33.17, 32.62, 31.38, 31.07, 28.74, 27.63, 26.40, 24.03, 23.30, 23.23, 17.58.

##### 7α-Hydroxy-3β-[(7-nitro-2,1,3-benzoxadiazol-4-yl)­amino]-5β-oxocholane-24-yl]­amino] Ethane Trifluoroethanesulfonic Acid Ester (**13b**)

This compound was prepared according to general procedure G using **6** (0.165 g, 0.297 mmol) to obtain compound **13b** as an orange solid (0.137 g, 0.184 mmol, 62%).

HRMS (ESI): *m*/*z* = 766.3074 [M + Na]^+^ (calculated for 766.3068)

^1^H NMR (CDCl_3_, 700.1 MHz): δ [ppm] = 8.49 (d, *J* = 8.6 Hz, 1H), 6.39 (d, *J* = 7.3 Hz, 1H), 6.17 (d, *J* = 8.6 Hz, 1H), 6.05–5.98 (m, 1H), 4.54 (q, *J* = 7.9 Hz, 2H), 4.09–4.01 (m, 1H), 3.93–3.87 (m, 1H), 3.81–3.72 (m, 2H), 3.54–3.42 (m, 2H), 2.78 (td, *J* = 14.5, 4.0 Hz, 1H), 2.31–2.23 (m, 1H), 2.13–2.04 (m, 2H), 2.03–1.98 (m, 1H), 1.97–1.85 (m, 3H), 1.84–1.72 (m, 4H), 1.66–1.59 (m, 3H), 1.57–1.51 (m, 2H), 1.51–1.45 (m, 2H), 1.44–1.39 (m, 2H), 1.38–1.29 (m, 3H), 1.27–1.11 (m, 3H), 1.02 (s, 3H), 0.94 (d, *J* = 6.5 Hz, 3H), 0.68 (s, 3H).

^13^C NMR (CDCl_3_, 176.6 MHz): δ [ppm] = 174.09, 144.65, 144.14, 136.74, 124.43, 123.83, 99.03, 68.62, 64.11 (q, *J* = 38.2 Hz), 56.00, 51.17, 50.55, 49.85, 42.92, 39.72, 39.51, 37.45, 35.78, 35.61, 35.59, 34.15, 34.08, 33.40, 32.73, 32.51, 31.54, 31.16, 28.32, 24.15, 23.88, 23.49, 21.01, 18.49, 11.95.

##### 7α,12α-Dihydroxy-3α-[(7-nitro-2,1,3-benzoxadiazol-4-yl)­amino]-5β-oxocholane-24-yl]­amino] Ethane Trifluoroethanesulfonic Acid Ester (**13d**)

This compound was prepared according to general procedure G using **6d** (0.151 g, 0.265 mmol) to obtain compound **13d** as an orange solid (0.114 g, 0.150 mmol, 59%).

HRMS (ESI): *m*/*z* = 782.3015 [M + Na]^+^ (calculated for 782.3017)

^1^H NMR (CDCl_3_, 400.1 MHz): δ [ppm] = 8.44 (d, *J* = 8.6 Hz, 1H), 6.63 (s, 1H), 6.45 (s, 1H), 6.15 (d, *J* = 8.7 Hz, 1H), 4.61–4.47 (m, 2H), 4.02 (s, 1H), 3.89 (d, *J* = 3.7 Hz, 1H), 3.82–3.71 (m, 2H), 3.60–3.42 (m, 2H), 2.87 (s, 1H), 2.53–2.37 (m, 3H), 2.30–2.22 (m, 2H), 2.20–2.11 (m, 1H), 2.04–1.99 (m, 1H), 1.96–1.81 (m, 5H), 1.80–1.70 (m, 2H), 1.66–1.53 (m, 6H), 1.49–1.37 (m, 2H), 1.37–1.24 (m, 2H), 1.18–1.07 (m, 2H), 0.98 (m, 6H), 0.71 (s, 3H).

^13^C NMR (CDCl_3_, 100.6 MHz): δ [ppm] = 174.64, 144.54, 144.24, 136.96, 123.46, 123.12, 120.70, 73.12, 68.35, 64.42, 64.04, 50.94, 49.77, 46.86, 46.69, 42.20, 41.95, 39.65, 35.75, 35.59, 35.29, 34.95, 34.36, 34.25, 32.83, 31.34, 28.49, 27.85, 27.62, 26.98, 23.28, 22.77, 17.53, 12.71.

#### General Procedure H for the Synthesis of 3-NBD-TBA

The respective trifluoroethanol-protected 3-NBD-BA (1 equiv) was dissolved in DCM. Then, 2N sodium hydroxide in methanol (2 equiv) was added and the mixture was stirred for 3 h at room temperature. Distilled water was added, and the layers were separated. The organic layer was extracted three times with distilled water. The combined aqueous layers were lyophilized, and the crude product was purified by flash column chromatography.

##### 7α,12α-Dihydroxy-3β-[(7-nitro-2,1,3-benzoxadiazol-4-yl)­amino]-5β-oxocholan-24yl]­amino]­ethanesulfonic Acid (**14a**)

This compound was prepared according to general procedure H using **13a** (0.180 g, 0.237 mmol) to obtain compound **14a** as an orange solid (0.058 g, 0.083 mmol, 35%).

HRMS (ESI): *m*/*z* = 722.2806 [M + Na]^+^ (calculated for 722.2806)

HRMS (ESI): *m*/*z* = 676.3026 [M-Na]^−^ (calculated for 676.3022)

^1^H NMR (DMSO-*d*_6_, 400.1 MHz): δ [ppm] = 9.37–8.71 (m, 1H), 8.54–8.39 (m, 1H), 7.69 (t, *J* = 5.5 Hz, 1H), 6.79–6.27 (m, 1H), 4.24–4.12 (m, 1H), 4.13–4.00 (m, 1H), 3.82–3.74 (m, 1H), 3.70–3.60 (m, 1H), 3.32–3.24 (m, 2H), 3.16 (d, *J* = 4.9 Hz, 1H), 2.73 (s, 1H), 2.60–2.52 (m, 2H), 2.30–2.14 (m, 1H), 2.14–1.91 (m, 3H), 1.87–1.49 (m, 10H), 1.49–1.34 (m, 5H), 1.30–1.12 (m, 4H), 0.97–0.86 (m, 6H), 0.59 (s, 3H).

^13^C NMR (DMSO-*d*_6_, 100.6 MHz): δ [ppm] = 172.19, 144.60, 137.64, 127.71, 125.93, 107.12, 100.35, 71.02, 67.18, 66.26, 50.61, 48.59, 46.14, 45.81, 41.36, 39.43, 36.40, 35.46, 35.13, 34.57, 34.16, 32.72, 31.61, 29.08, 28.69, 27.27, 24.96, 22.78, 22.62, 19.98, 17.13, 12.36.

##### 7α-Hydroxy-3β-[(7-nitro-2,1,3-benzoxadiazol-4-yl)­amino]-5β-oxocholan-24-yl]­amino]­ethanesulfonic Acid (**14b**)

This compound was prepared according to general procedure H using **13b** (0.127 g, 0.171 mmol) to obtain compound **14b** as an orange solid (0.021 g, 0.032 mmol, 19%).

HRMS (ESI): *m*/*z* = 660.3066 [M-Na]^−^ (calculated for 660.3073)

^1^H NMR (DMSO-*d*_6_, 400.1 MHz): δ [ppm] = 8.94 (s, 1H), 8.53–8.39 (m, 1H), 7.78–7.60 (m, 1H), 6.38 (s, 1H), 4.22 (d, *J* = 3.5 Hz, 1H), 4.16–3.92 (m, 1H), 3.67–3.53 (m, 1H), 3.31–3.21 (m, 2H), 2.84–2.60 (m, 1H), 2.56–2.51 (m, 2H), 2.16–2.00 (m, 1H), 1.98–1.84 (m, 2H), 1.80–1.60 (m, 8H), 1.58–1.45 (m, 1H), 1.44–1.28 (m, 6H), 1.23–1.03 (m, 7H), 0.92 (s, 3H), 0.88 (d, *J* = 6.4 Hz, 3H), 0.61 (s, 3H).

^13^C NMR (DMSO-*d*_6_, 100.6 MHz): δ [ppm] = 172.10, 155.74, 149.14, 144.54, 105.59, 88.01, 79.99, 69.57, 66.19, 63.95, 55.54, 50.61, 49.96, 41.96, 41.35, 36.28, 35.46, 35.01, 34.94, 34.13, 32.57, 32.33, 31.50, 30.87, 29.08, 27.77, 24.96, 23.15, 22.72, 20.50, 18.34, 11.68.

##### 7α,12α-Dihydroxy-3α-[(7-nitro-2,1,3-benzoxadiazol-4-yl)­amino]-5β-oxocholan-24-yl]­amino]­ethanesulfonic Acid (**14d**)

This compound was prepared according to general procedure H using **13d** (0.103 g, 0.136 mmol) to obtain compound **14d** as an orange solid (0.075 g, 0.107 mmol, 79%).

HRMS (ESI): *m*/*z* = 722.2806 [M + Na]^+^ (calculated for 722.2806)

HRMS (ESI): *m*/*z* = 676.3022 [M-Na]^−^ (calculated for 676.3028)

^1^H NMR (DMSO-*d*_6_, 400.1 MHz): δ [ppm] = 9.59 (s, 1H), 8.45 (d, *J* = 8.9 Hz, 1H), 7.70 (t, *J* = 5.5 Hz, 1H), 6.43 (d, *J* = 9.4 Hz, 1H), 4.15 (d, *J* = 3.6 Hz, 1H), 4.12 (s, 1H), 3.81 (d, *J* = 3.5 Hz, 1H), 3.66–3.52 (m, 2H), 3.31–3.25 (m, 2H), 3.16 (d, *J* = 4.1 Hz, 1H), 2.78–2.53 (m, 2H), 2.28–2.21 (m, 1H), 2.10–1.89 (m, 3H), 1.85–1.72 (m, 4H), 1.70–1.56 (m, 4H), 1.52–1.35 (m, 5H), 1.30–1.02 (m, 6H), 0.93 (d, *J* = 6.4 Hz, 3H), 0.90 (s, 3H), 0.60 (s, 3H).

^13^C NMR (DMSO-*d*_6_, 100.6 MHz): δ [ppm] = 172.19, 144.54, 137.89, 127.71, 125.93, 119.80, 99.30, 70.93, 66.13, 62.79, 54.36, 50.60, 48.58, 46.13, 45.79, 41.71, 41.40, 35.45, 35.23, 34.69, 34.52, 32.78, 31.59, 28.67, 27.77, 27.34, 26.33, 26.01, 22.81, 22.71, 17.11, 12.38.

#### General Procedure I for the Synthesis of 3-NBD-GBA Methyl Ester

Under nitrogen atmosphere, the respective 3-NBD-BA (1 equiv) was dissolved in anhydrous DMF. Triethylamine (3.5 equiv), TBTU (1.5 equiv), and HOBt·H_2_O (1.5 equiv) were added, and the mixture was stirred for 45 min at room temperature. Then, glycine methyl ester hydrochloride (1.1 equiv) in DMF was added, and the mixture was stirred for 18 h at room temperature. Distilled water was added, and the aqueous layer was extracted three times with ethyl acetate. The combined organic layers were washed with sodium bicarbonate, potassium bisulfate, sodium bicarbonate, distilled water, and brine. The organic layer was dried over MgSO_4,_ and the solvent was removed under reduced pressure. The crude product was purified by flash column chromatography.

##### 7α,12α-Dihydroxy-3β-[(7-nitro-2,1,3-benzoxadiazol-4-yl)­amino]-5β-oxocholan-24-yl]­glycine Methyl Ester (**15a**)

This compound was prepared according to general procedure I using **6a** (0.200 g, 0.350 mmol) to obtain compound **15a** as an orange solid (0.108 g, 0.168 mmol, 48%).

HRMS (ESI): *m*/*z* = 664.3320 [M + Na]^+^ (calculated for 664.3317)

^1^H NMR (DMSO-*d*_6_, 400.1 MHz): δ [ppm] = 9.37–8.70 (m, 1H), 8.65–8.32 (m, 1H), 8.22 (t, *J* = 5.9 Hz, 1H), 6.89–6.19 (m, 1H), 4.22–4.09 (m, 2H), 4.06 (s, 1H), 3.80 (d, *J* = 5.8 Hz, 2H), 3.63 (s, 1H), 3.62 (s, 3H), 2.76 (s, 1H), 2.25–2.10 (m, 2H), 2.09–1.95 (m, 2H), 1.87–1.56 (m, 10H), 1.49–1.26 (m, 8H), 1.26–1.10 (m, 2H), 0.94 (d, *J* = 6.4 Hz, 3H), 0.90 (s, 3H), 0.60 (s, 3H).

^13^C NMR (DMSO-*d*_6_, 100.6 MHz): δ [ppm] = 173.22, 170.53, 144.45, 137.69, 128.47, 100.16, 97.83, 71.03, 66.25, 58.24, 51.59, 51.17, 47.42, 46.18, 45.80, 41.37, 40.50, 39.40, 36.38, 35.37, 35.10, 34.90, 34.55, 34.15, 32.12, 31.55, 28.69, 27.25, 26.13, 22.78, 22.60, 17.09, 12.34.

##### 7α,12α-Dihydroxy-3α-[(7-nitro-2,1,3-benzoxadiazol-4-yl)­amino]-5β-oxocholan-24-yl]­glycine Methyl Ester (**15d**)

This compound was prepared according to general procedure I using **6d** (0.209 g, 0.366 mmol) to obtain compound **15d** as an orange solid (0.137 g, 0.213 mmol, 58%).

HRMS (ESI): *m*/*z* = 664.3315 [M + Na]^+^ (calculated for 664.3317)

^1^H NMR (CDCl_3_, 400.1 MHz): δ [ppm] = 9.62 (d, *J* = 7.8 Hz, 1H), 8.47 (d, *J* = 9.0 Hz, 1H), 8.21 (t, *J* = 6.0 Hz, 1H), 6.44 (d, *J* = 9.1 Hz, 1H), 4.14 (d, *J* = 3.5 Hz, 1H), 4.12–4.07 (m, 1H), 3.91–3.82 (m, 1H), 3.82 (d, *J* = 5.9 Hz, 2H), 3.69–3.62 (m, 1H), 3.61 (s, 3H), 2.65–2.53 (m, 1H), 2.33–2.21 (m, 1H), 2.21–2.10 (m, 1H), 2.09–1.93 (m, 2H), 1.92–1.55 (m, 10H), 1.52–1.36 (m, 5H), 1.33–1.24 (m, 1H), 1.23–1.15 (m, 2H), 1.12–0.98 (m, 2H), 0.94 (d, *J* = 6.4 Hz, 3H), 0.90 (s, 3H), 0.61 (s, 3H).

^13^C NMR (CDCl_3_, 100.6 MHz): δ [ppm] = 173.70, 171.01, 144.94, 143.62, 138.42, 120.42, 99.73, 84.37, 71.43, 66.61, 62.36, 54.79, 52.07, 46.69, 46.27, 42.18, 41.90, 40.98, 40.67, 35.94, 35.67, 35.18, 34.99, 32.70, 32.02, 29.16, 27.81, 26.81, 26.47, 23.28, 23.18, 17.55, 12.86.

#### General Procedure J for the Synthesis of 3-NBD-GBA

To a solution of the respective 3-NBD-GBA methyl ester (1 equiv) in methanol, 2N sodium hydroxide in methanol (10 equiv) was added at 0 °C. The mixture was stirred for 3 h at room temperature. Distilled water was added, and the mixture was adjusted to pH 2 by addition of 2 N HCl. The aqueous layer was extracted three times with ethyl acetate and the combined organic layers were dried over MgSO_4_. The solvent was removed under reduced pressure and the crude product was purified by flash column chromatography.

##### 7α,12α-Dihydroxy-3β-[(7-nitro-2,1,3-benzoxadiazol-4-yl)­amino]-5β-oxocholan-24-yl]­glycine (**16a**)

This compound was prepared according to general procedure J using **15a** (0.237 g, 0.364 mmol) to obtain compound **16a** as an orange solid (0.197 g, 0.314 mmol, 85%).

HRMS (ESI): *m*/*z* = 650.3163 [M + Na]^+^ (calculated for 650.3160)

^1^H NMR (DMSO-*d*_6_, 400.1 MHz): δ [ppm] = 8.56–8.30 (m, 1H), 7.84 (d, *J* = 6.0 Hz, 1H), 6.56 (d, *J* = 137.0 Hz, 1H), 4.23–4.09 (m, 1H), 4.09–3.98 (m, 1H), 3.79 (d, *J* = 3.0 Hz, 1H), 3.67–3.59 (m, 3H), 2.81–2.66 (m, 1H), 2.24–2.09 (m, 2H), 2.06–1.94 (m, 2H), 1.88–1.09 (m, 22H), 0.94 (d, *J* = 6.3 Hz, 3H), 0.89 (s, 3H), 0.59 (s, 3H).

^13^C NMR (DMSO-*d*_6_, 100.6 MHz): δ [ppm] = 172.68, 144.58, 137.65, 127.72, 120.58, 112.18, 100.24, 71.04, 66.26, 50.60, 49.94, 48.59, 46.21, 45.81, 41.63, 41.38, 39.42, 36.38, 35.19, 34.57, 34.17, 32.32, 31.64, 30.79, 29.09, 28.70, 27.28, 26.17, 22.80, 22.62, 17.15, 12.37.

##### 7α,12α-Dihydroxy-3α-[(7-nitro-2,1,3-benzoxadiazol-4-yl)­amino]-5β-oxocholan-24-yl]­glycine (**16d**)

This compound was prepared according to general procedure J using **15d** (0.121 g, 0.189 mmol) to obtain compound **16d** as an orange solid (0.075 g, 0.119 mmol, 63%).

HRMS (ESI): *m*/*z* = 650.3164 [M + Na]^+^ (calculated for 650.3160)

^1^H NMR (DMSO-*d*_6_, 400.1 MHz): δ [ppm] = 8.44 (d, *J* = 9.1 Hz, 1H), 7.66 (d, *J* = 5.5 Hz, 1H), 6.42 (d, *J* = 9.3 Hz, 1H), 4.45–4.20 (m, 1H), 4.17–4.08 (m, 1H), 3.86–3.78 (m, 1H), 3.66–3.61 (m, 1H), 3.56 (d, *J* = 5.3 Hz, 2H), 2.66–2.52 (m, 1H), 2.32–2.10 (m, 3H), 2.08–1.95 (m, 2H), 1.90–1.74 (m, 5H), 1.70–1.53 (m, 5H), 1.51–1.32 (m, 6H), 1.30–1.04 (m, 5H), 0.93 (d, *J* = 6.4 Hz, 3H), 0.89 (s, 3H), 0.60 (s, 3H).

^13^C NMR (DMSO-*d*_6_, 100.6 MHz): δ [ppm] = 172.37, 144.58, 144.31, 137.71, 127.73, 125.93, 103.34, 99.34, 70.96, 66.15, 54.46, 48.60, 46.25, 45.79, 42.49, 41.70, 41.41, 35.49, 35.34, 34.93, 34.71, 34.53, 32.50, 31.63, 28.69, 27.37, 26.34, 26.07, 22.84, 22.72, 17.14, 12.40.

## Supplementary Material





## Notes

Additional data on the compounds, including structural formula, additional synthetic procedures, ^1^H and ^13^C NMR spectra, and HPLC chromatograms (PDF)
Molecular formula strings, SMILES­(CSV)

## Abbreviations

ASBT: apical sodium-dependent bile acid transporter
BA: bile acid
BS: bile salt
BSEP: bile salt export pump
CA: cholic acid
CamF: cholyl-amido-fluorescein
CDCA: chenodeoxycholic acid
CGamF: cholyl-glycyl-amido-fluorescein
CLF: cholyl-l-lysyl-fluorescein
DBD: 4-*N*,*N*-dimethylaminosulfobenzo-2-oxa-1,3-diazole
DCA: deoxycholic acid
DHEAS: dehydroepiandrosterone sulfate
EHC: enterohepatic circulation
GCA: glycocholic acid
IC_50_: half maximal inhibitory concentration
NBD: 4-nitrobenzo-2-oxa-1,3-diazole
NTCP: Na^+^/taurocholate cotransporting polypeptide
OATP: organic anion transporting polypeptide
SOAT: sodium-dependent organic anion transporter
TCA: taurocholic acid
TCDCA: taurochenodeoxycholic acid
UDCA: ursodeoxycholic acid
