# Supplementary material for: Fluorescent 4‑Nitrobenzo-2-oxa-1,3-diazole-Coupled Bile Acids as Probe Substrates of Hepatic and Intestinal Bile Acid Transporters of the Solute Carrier Families SLC10 and SLCO
Source: J Med Chem. 2025 May 17;68(11):11724–45. doi: 10.1021/acs.jmedchem.5c00589 (PMC12169615; doi:10.1021/acs.jmedchem.5c00589)
Supplement: Supplementary file 1 [file jm5c00589_si_001.pdf]

Supporting Information

Fluorescent 4-nitrobenzo-2-oxa-1,3-diazole-coupled bile acids as probe substrates of hepatic and intestinal bile acid transporters of the solute carrier families SLC10 and SLCO

Celine Drossel[1], Sebastian Kunz[2], Christopher Neelen[2], Mats Georg[1], Yohannes Hagos[3], Dieter Glebe[4], Richard Göttlich[1], Joachim Geyer[2*]

[1]Institute of Organic Chemistry, Justus Liebig University Giessen, Heinrich-Buff-Ring 17, 35392 Giessen, Germany

[2]Institute of Pharmacology and Toxicology, Justus Liebig University Giessen, Schubertstr. 81, 35392 Giessen, Germany

[3]PortaCellTec Biosciences GmbH, Science Park Va, Marie-Curie-Strasse 8, 37079 Göttingen Germany

[4]Institute of Medical Virology, Justus Liebig University of Giessen, National Reference Centre for Hepatitis B viruses and Hepatitis D viruses, German Center for Infection Research (DZIF), partner site Giessen-Marburg-Langen, Schubertstr. 81, 35392, Giessen, Germany

**Corresponding author, E-mail: Joachim.M.Geyer@vetmed.uni-giessen.de*

Contents of SI

Additional Synthetic Procedures 2

^{1}H and ^{13}C NMR Spectra 5

HPLC Chromatograms 46

Time-dependent Transport (Addition to Figure 6 and Figure 7) 55

95% Confidence Intervals (CI) of the K_m and V_{max} values (Addition to Table 1) 57

Additional Synthetic Procedures

Synthesis of Methyl-7α,12α-dihydroxy-3β-(acetyloxy)-5β-cholan-24-oate

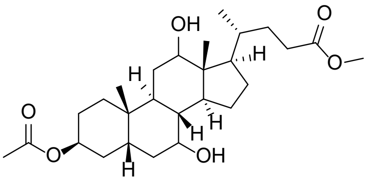

Under nitrogen atmosphere methyl-3α,7α,12α-trihydroxy-5β-cholan-24-oate (1.001 g, 2.369 mmol, 1 equiv.) and triphenylphosphine (0.744 g, 2.839 mmol, 1.2 equiv.) were dissolved in 25 mL anhydrous THF and acetic acid (0.16 mL, 2.84 mmol. 1.2 equiv.) was added. The mixture was cooled to 0 °C and DIAD (0.56 mL, 2.84 mmol, 1.2 equiv.) was added dropwise. The mixture was stirred for 18 h at 50 °C. Then the solvent was removed under reduced pressure and the crude product was purified by flash column chromatography (ethyl acetate/cyclohexane 1:2) to obtain the product as a white solid (0.894 g, 1.924 mmol, 81 %).

HRMS (ESI): m/z = 487.3030 $[M+Na]^+$ (calculated for 487.3030)

^{1}H-NMR ($CDCl_3$, 400.1 MHz): δ [ppm] = 5.05-4.94 (m, 1H), 4.01-3.92 (m, 1H), 3.88-3.79 (m, 1H), 3.65 (s, 3H), 2.48 (ddd, *J* = 14.9, 13.3, 3.0 Hz, 1H), 2.41-2.31 (m, 1H), 2.29-2.11 (m, 2H), 2.03 (s, 3H), 1.98-1.84 (m, 5H), 1.80-1.50 (m, 11H), 1.46-1.23 (m, 5H), 1.19-1.05 (m, 1H), 0.97 (d, *J* = 6.2 Hz, 3H), 0.93 (s, 3H), 0.68 (s, 3H).

^{13}C-NMR ($CDCl_3$, 100.6 MHz): δ [ppm] = 174.88, 170.92, 73.14, 70.85, 68.61, 51.64, 47.37, 46.71, 42.09, 39.60, 36.93, 35.35, 35.14, 34.28, 33.69, 31.21, 30.98, 30.67, 28.72, 27.60, 26.13, 24.95, 23.35, 23.07, 21.66, 17.46, 12.66.

Synthesis of *tert*-Butyl-3α,7α,12α-trihydroxy-5β-cholan-24-oate

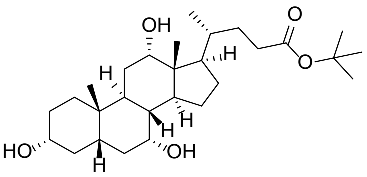

Under nitrogen atmosphere CA (5.000 g, 12.238 mmol, 1 equiv.) was dissolved in 80 mL anhydrous THF. TFA anhydride (12.50 mL, 89.87 mmol, 7.3 equiv.) was added at 0 °C and the mixture was stirred for 1.5 h at room temperature. Then *tert*-butanol (29.50 mL, 314.512 mmol, 25.7 equiv.) was added and the mixture was stirred for 18 h at room temperature. 25 mL of 25 % ammonia solution were added and the mixture was stirred for another 18 h at room temperature. The solvent was removed under reduced pressure, the crude product was dissolved in 20 mL diethyl ether and washed with 15 mL 1N sodium hydroxide and 15 mL distilled water. The organic layer was dried over $MgSO_4$ and 25 mL acetonitrile were added. The solvent was removed under reduced pressure at room temperature and the remaining solid was dried under vacuum. The product was obtained as a white solid (5.269 g, 11.339 mmol, 93 %).

HRMS (ESI): m/z = 487.3396 [M+Na]$^+$ (calculated for 487.3394)

^{1}H-NMR ($CDCl_3$, 400.1 MHz): δ [ppm] = 4.03-3.92 (m, 1H), 3.92-3.79 (m, 1H), 3.58-3.40 (m, 1H), 2.93 (s, 3H), 2.38-2.09 (m, 4H), 1.97-1.84 (m, 3H), 1.80-1.48 (m, 12H), 1.44 (s, 9H), 1.41-1.23 (m, 4H), 1.20-1.06 (m, 1H), 0.97 (d, *J* = 6.3 Hz, 3H), 0.89 (s, 3H), 0.68 (s, 3H).

^{13}C-NMR ($CDCl_3$, 100.6 MHz): δ [ppm] = 173.88, 80.09, 73.24, 72.26, 68.62, 47.34, 46.62, 41.97, 41.57, 39.68, 39.57, 35.36, 35.28, 34.85, 34.68, 32.74, 31.10, 30.44, 28.34, 28.27, 27.62, 26.70, 23.33, 22.63, 17.50, 12.65.

Synthesis of *tert*-Butyl-7α,12α-dihydroxy-3β-(acetyloxy)-5β-cholan-24-oate

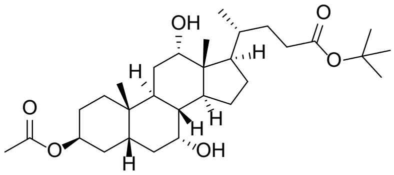

Under nitrogen atmosphere *tert*-butyl-3α,7α,12α-trihydroxy-5β-cholan-24-oate (2.501 g, 5.382 mmol, 1 equiv.) and triphenylphosphine (1.694 g, 6.459 mmol, 1.2 equiv.) were dissolved in 45 mL anhydrous THF and acetic acid (0.37 mL, 6.46 mmol, 1.2 equiv.) was added. The mixture was cooled to 0 °C and DIAD (1.27 mL, 6.46 mmol, 1.2 equiv.) was added dropwise. The mixture was stirred for 18 h at 50 °C. Then the solvent was removed under reduced pressure and the crude product was purified by flash column chromatography (ethyl acetate/cyclohexane 1:2) to obtain the product as a white solid (1.471 g, 1.632 mmol, 54 %).

HRMS (ESI): m/z = 529.3497 [M+Na]$^+$ (calculated for 529.3499)

^{1}H-NMR ($CDCl_3$, 400.1 MHz): δ [ppm] = 5.06-5.00 (m, 1H), 3.98 (t, *J* = 3.0 Hz, 1H), 3.94-3.83 (m, 1H), 2.49 (ddd, *J* = 15.6, 13.1, 3.0 Hz, 1H), 2.32.-2.22 (m, 1H), 2.22-2.08 (m, 2H), 2.03 (s, 3H), 2.01-1.85 (m, 3H), 1.78-1.64 (m, 6H), 1.61-1.54 (m, 5H), 1.52-1.45 (m, 2H), 1.43 (s, 9H), 1.37-1.24 (m, 5H), 1.19-1.10 (m, 1H), 0.97 (d, *J* = 6.5 Hz, 3H), 0.94 (s, 3H), 0.69 (s, 3H).

^{13}C-NMR ($CDCl_3$, 100.6 MHz): δ [ppm] = 173.82, 170.90, 80.11, 73.13, 70.85, 68.58, 47.47, 46.74, 42.17, 39.67, 36.95, 35.22, 34.20, 33.71, 32.69, 31.05, 30.67, 28.73, 28.27, 27.60, 26.23, 24.96, 23.33, 23.12, 22.10, 21.67, 17.51, 12.69.

Synthesis of *tert*-Butyl-3β,7α,12α-trihydroxy-5β-cholan-24-oate

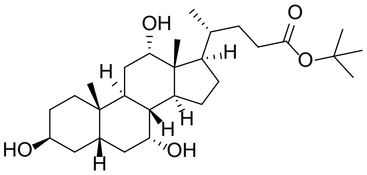

tert-Butyl-7α,12α-dihydroxy-3β-(acetyloxy)-5β-cholan-24-oate (1.442 g, 2.846 mmol, 1 equiv.) was dissolved in 30 mL methanol and 2N sodium hydroxide in methanol (14.20 mL, 28.40 mmol, 1 equiv.) was added. The mixture was stirred for 3.5 h at 40 °C. The solvent was removed under reduced pressure and the crude product was dissolved in 15 mL distilled water. The aqueous layer was three times extracted with 15 mL ethyl acetate and the combined organic layers were washed with 30 mL brine and then dried over $MgSO_4$. The solvent was removed under reduced pressure to obtain the product as a white solid (0.887 g, 1.909 mmol, 67 %).

HRMS (ESI): m/z = 487.3394 $[M+Na]^+$ (calculated for 487.3394)

^{1}H-NMR ($CDCl_3$, 400.1 MHz): δ [ppm] = 4.15-4.02 (m, 1H), 3.99 (d, *J* = 3.2 Hz, 1H), 3.86 (d, *J* = 3.0 Hz, 1H), 2.56-2.41 (m, 1H), 2.36-2.22 (m, 1H), 2.22-2.10 (m, 2H), 2.04-1.83 (m, 3H), 1.81-1.48 (m, 15H), 1.46 (s, 9H), 1.38-1.25 (m, 4H), 1.23-1.09 (m, 1H), 0.98 (d, *J* = 6.5 Hz, 3H), 0.94 (s, 3H), 0.70 (s, 3H).

^{13}C-NMR ($CDCl_3$, 100.6 MHz): δ [ppm] = 173.81, 80.12, 73.09, 68.62, 66.99, 47.49, 42.21, 39.68, 36.68, 36.10, 35.36, 35.20, 34.26, 32.71, 31.08, 29.86, 28.72, 28.28, 27.80, 27.59, 26.12, 23.34, 23.16, 22.10, 17.53, 12.71.

^{1}H and ^{13}C NMR Spectra

BA Methyl Ester

Methyl-3α,7α,12α-trihydroxy-5β-cholan-24-oate (1a)

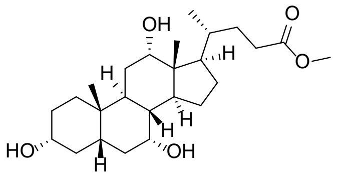

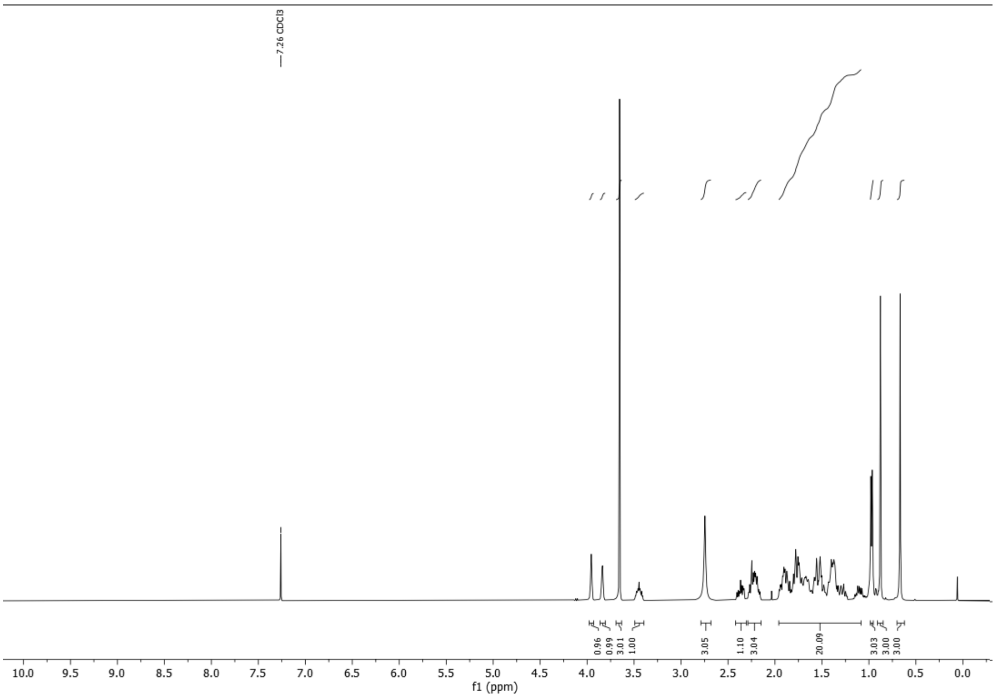

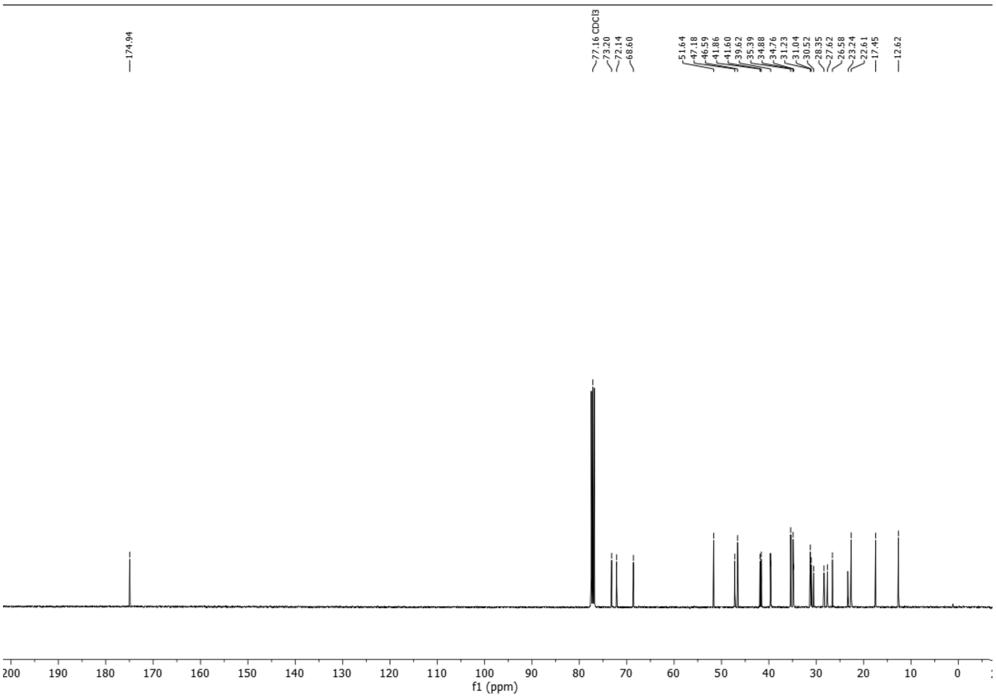

Methyl-3α,7α-dihydroxy-5β-cholan-24-oate (1b)

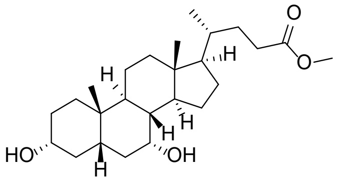

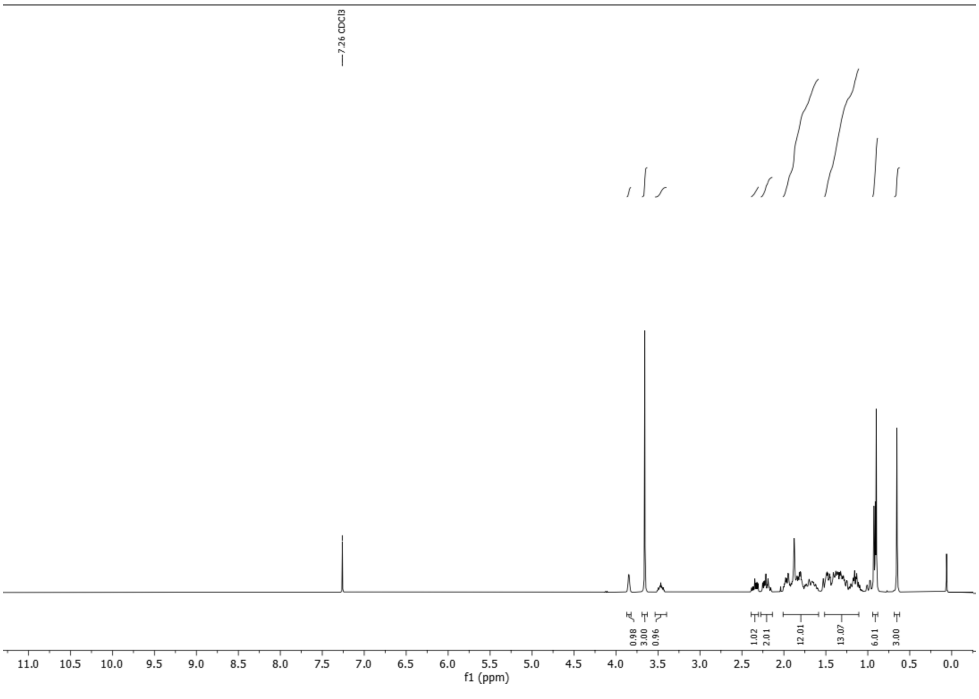

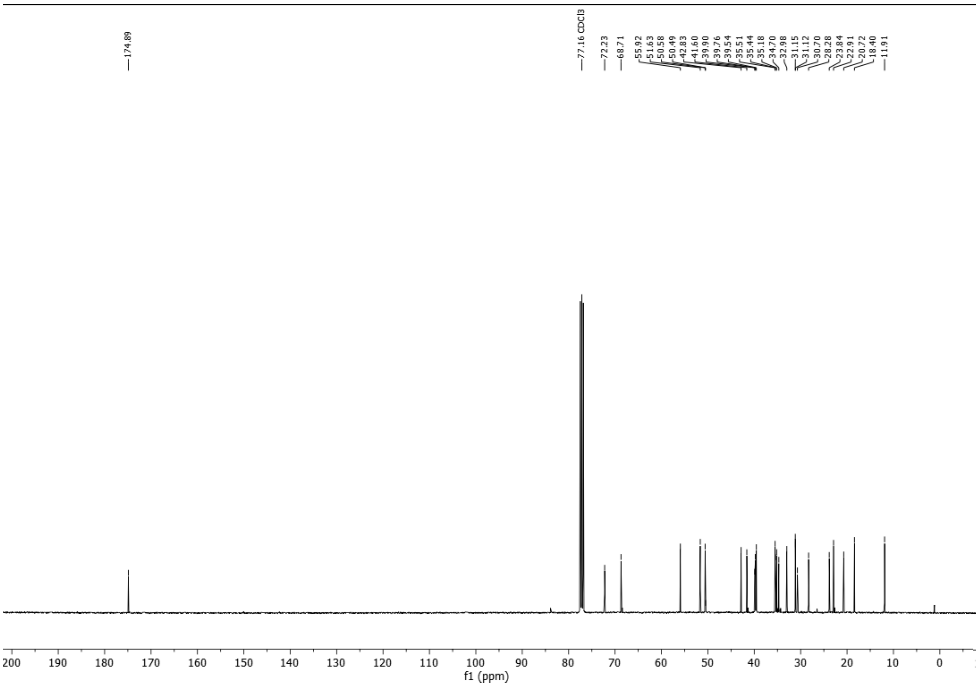

Methyl-3α,12α-dihydroxy-5β-cholan-24-oate (1c)

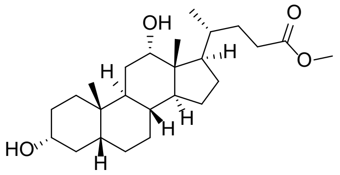

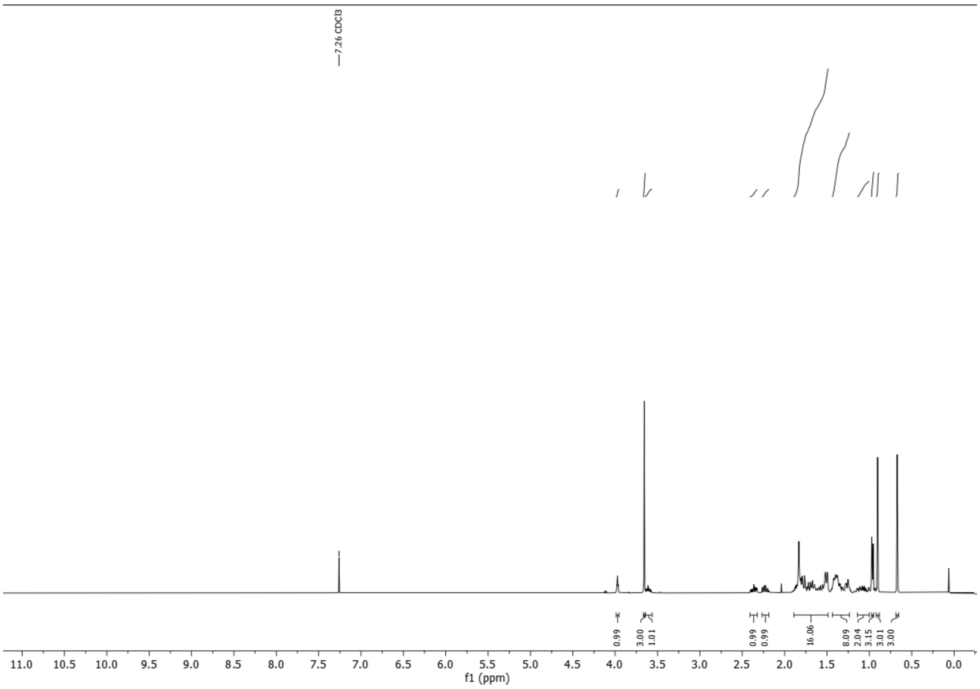

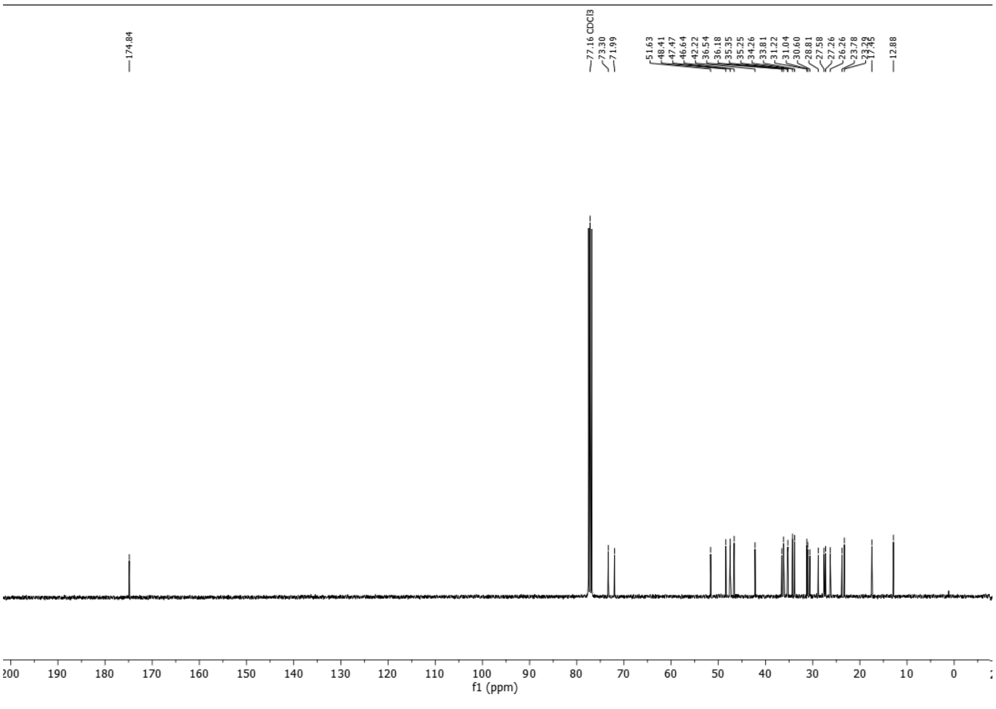

Mesyl BA Methyl Ester

Methyl-7α,12α-dihydroxy-3α-[(methylsulfonyl)oxy]-5β-cholan-24-oate (2a)

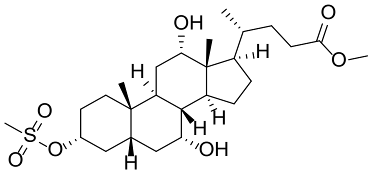

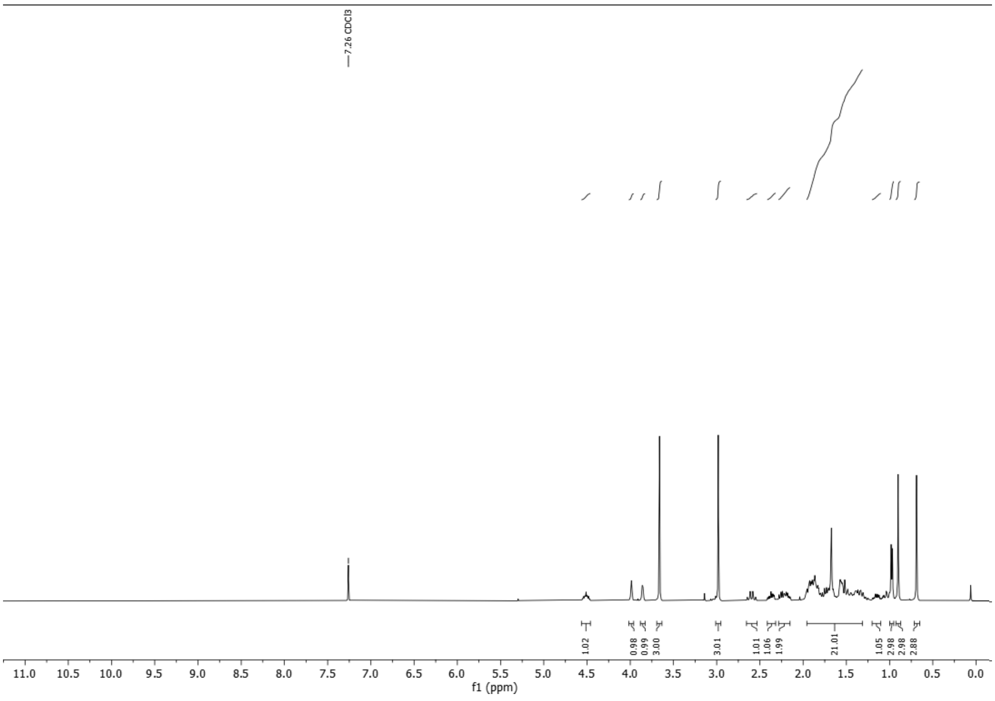

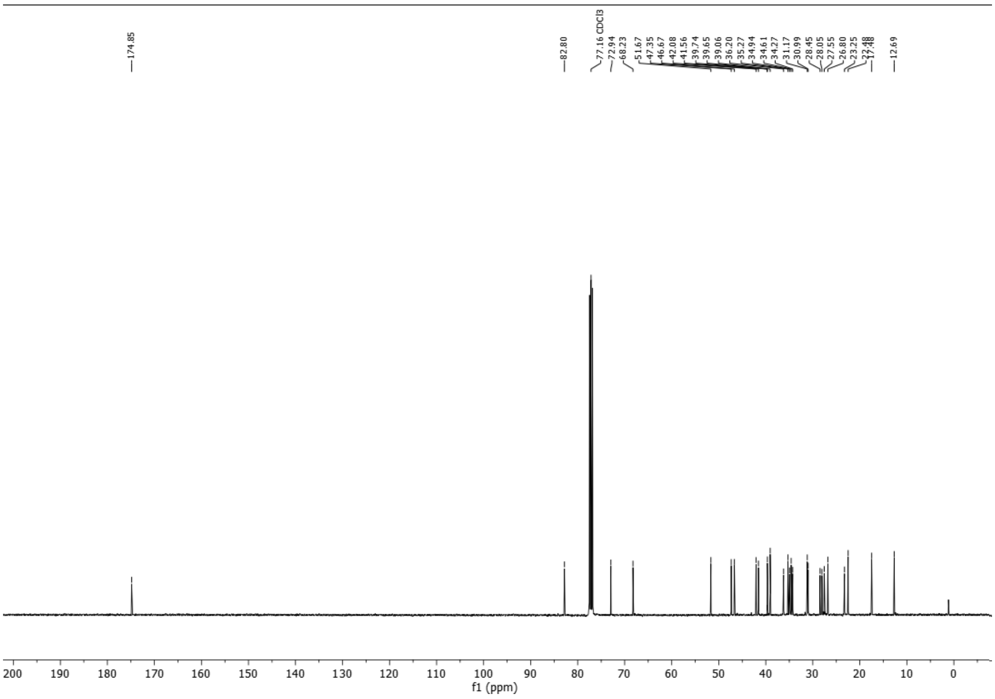

Methyl-7α-hydroxy-3α-[(methylsulfonyl)oxy]-5β-cholan-24-oate (2b)

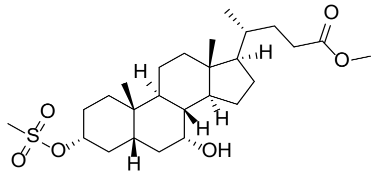

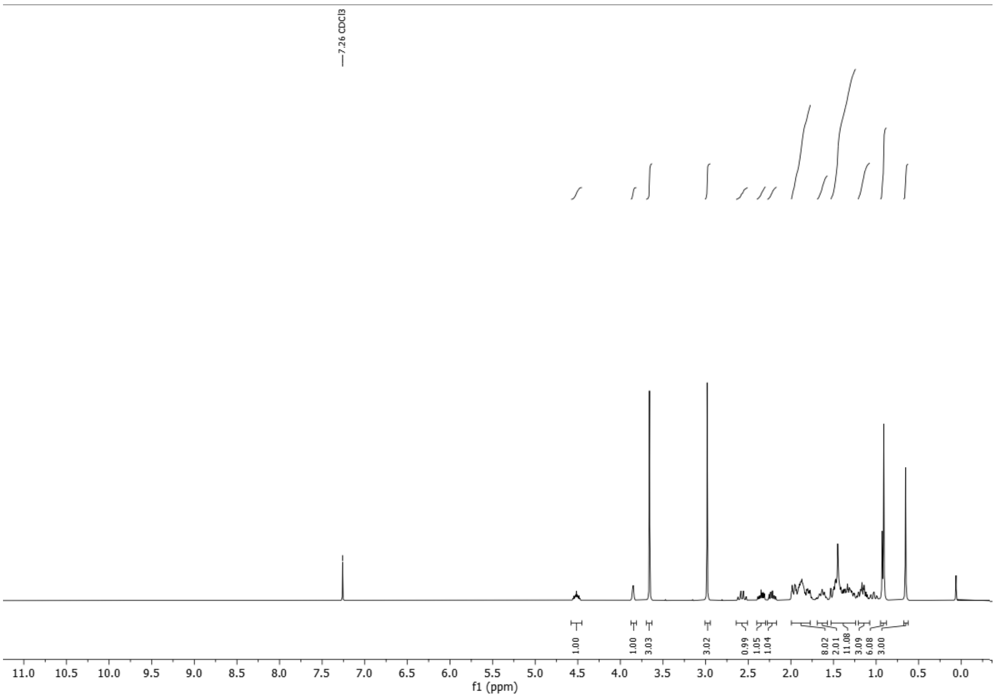

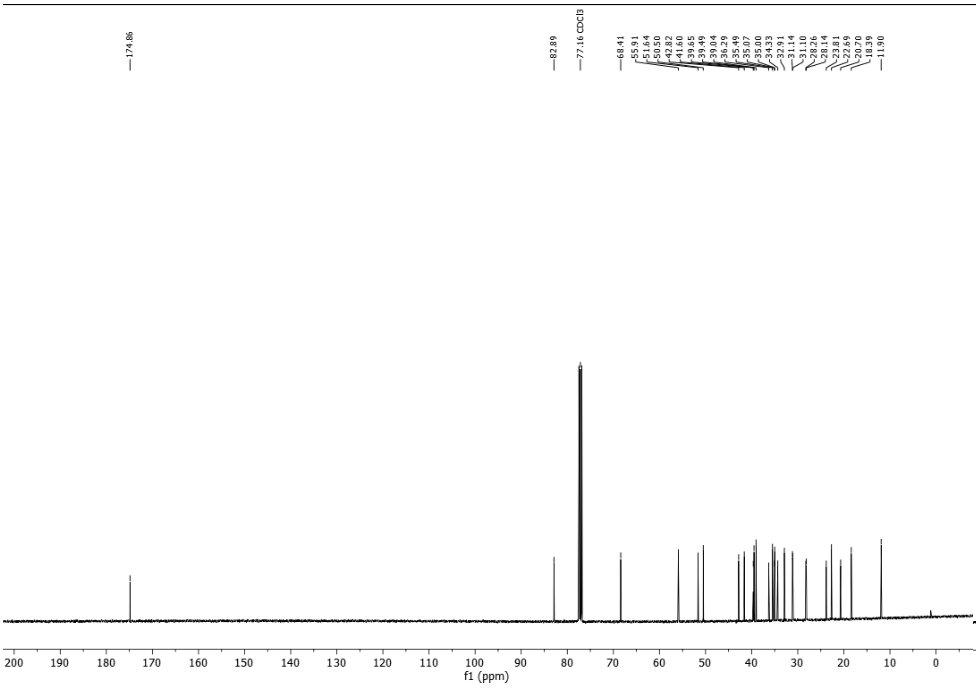

Methyl-12α-hydroxy-3α-[(methylsulfonyl)oxy]-5β-cholan-24-oate (2c)

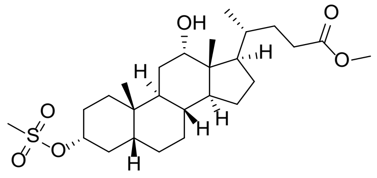

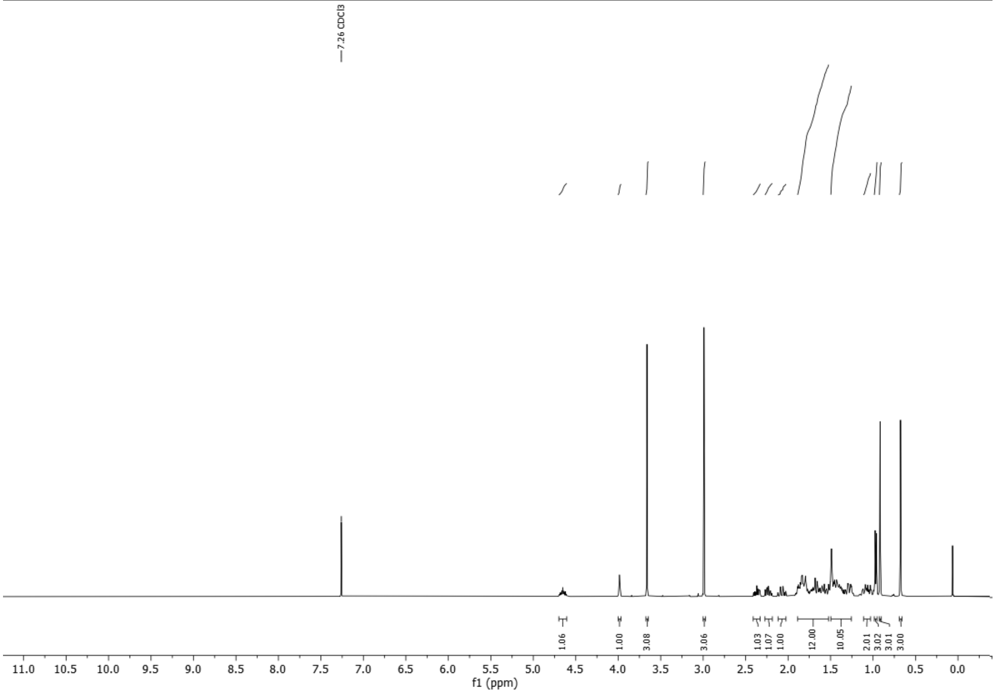

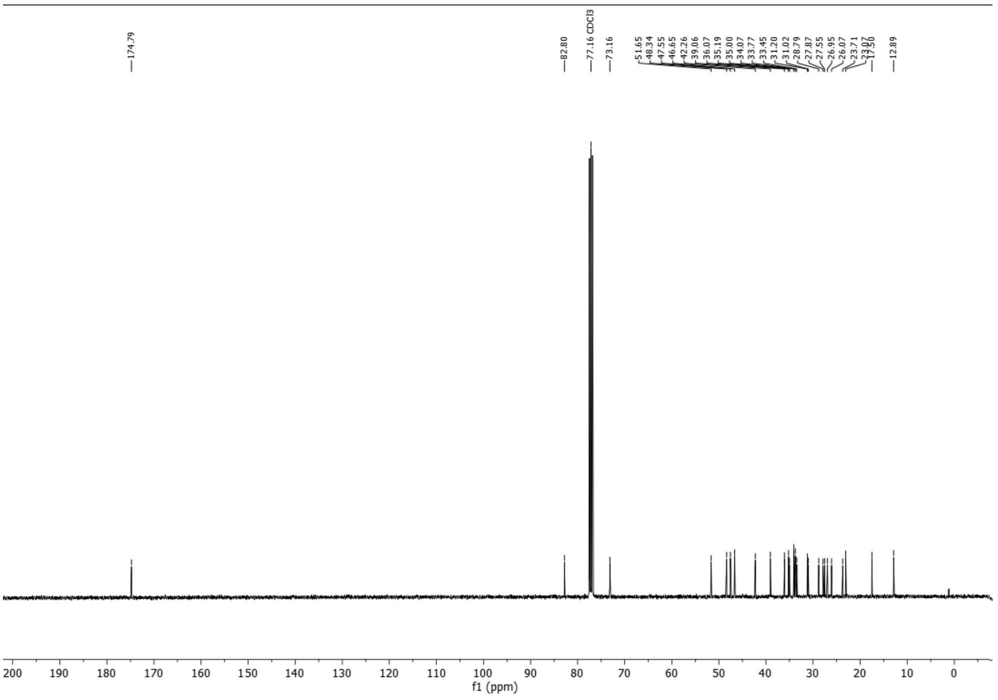

Azido BA Methyl Ester

Methyl-3β-azido-7α,12α-dihydroxy-5β-cholan-24-oate (3a)

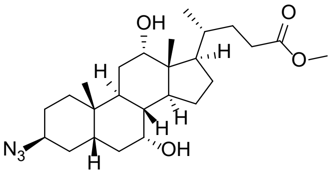

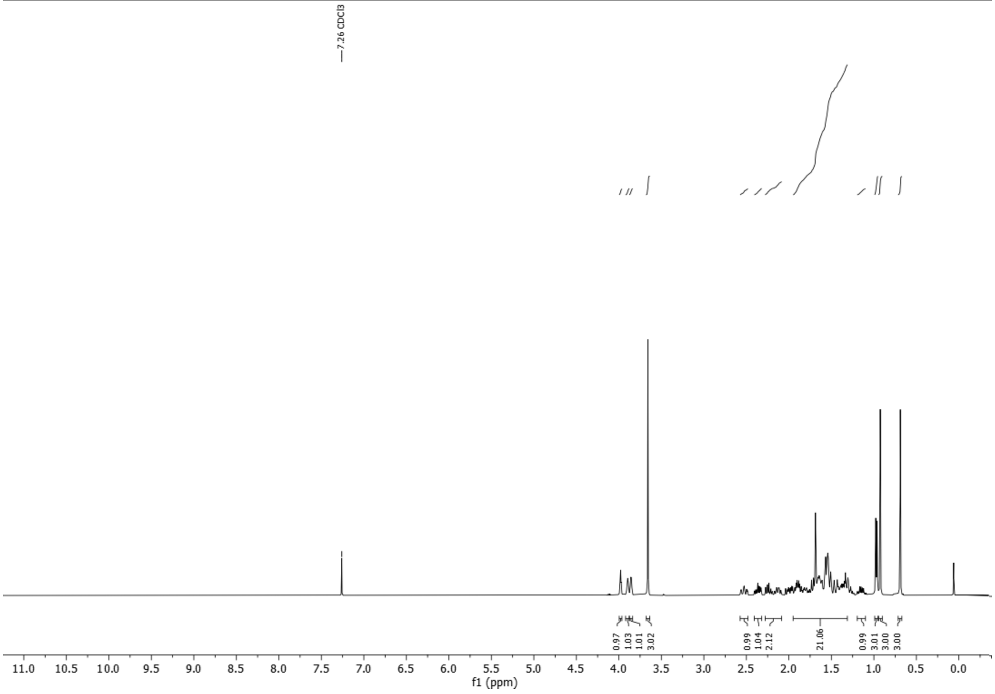

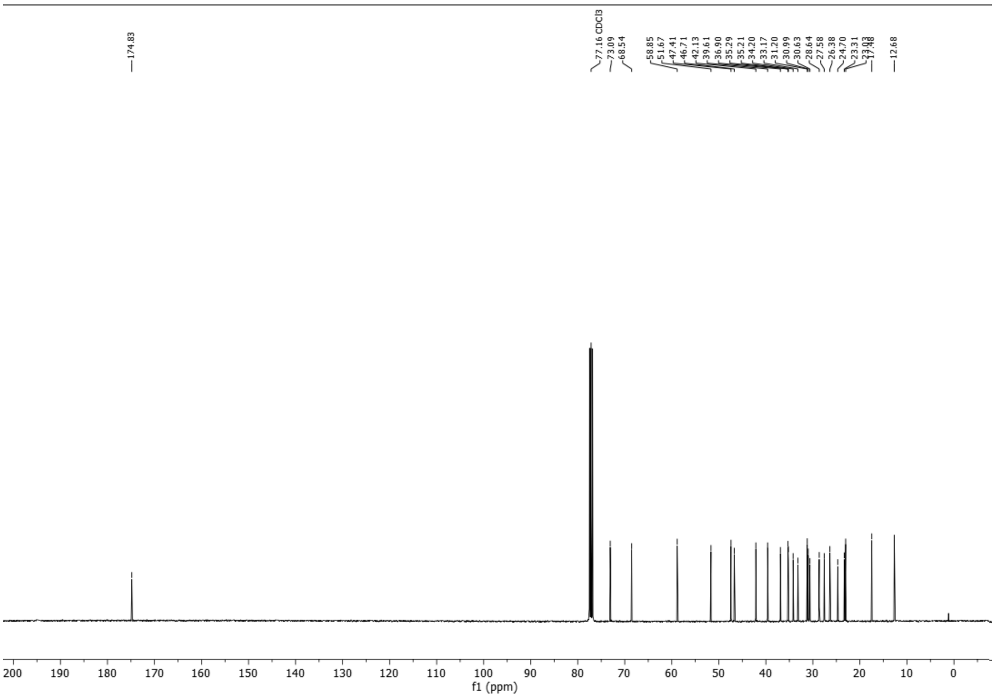

Methyl-3α-azido-7α,12α-dihydroxy-5β-cholan-24-oate (3d)

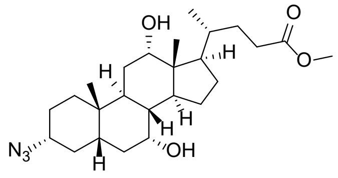

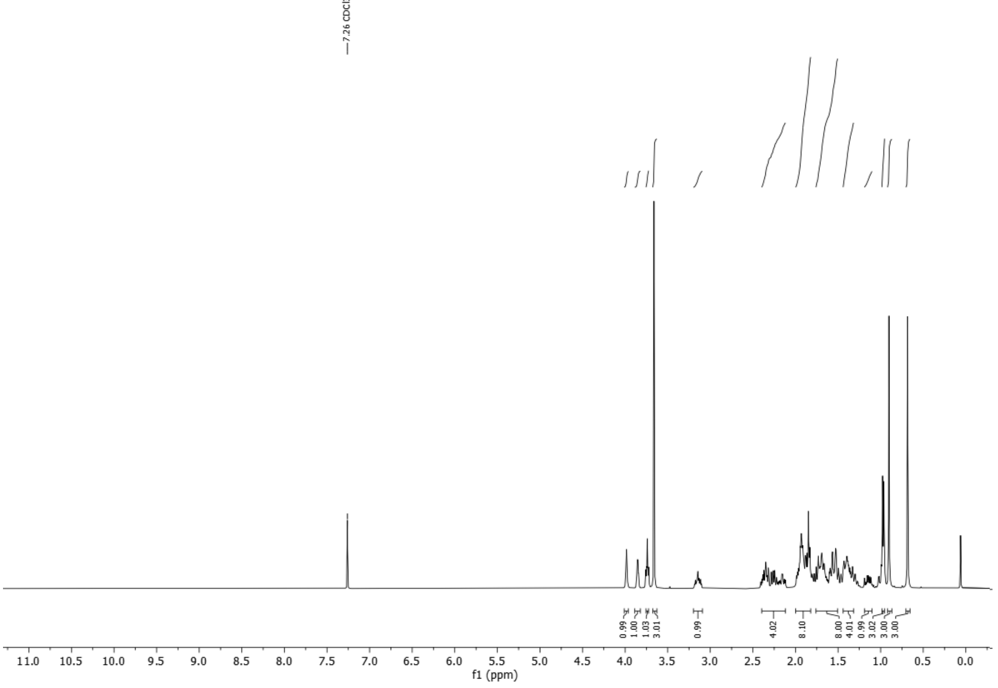

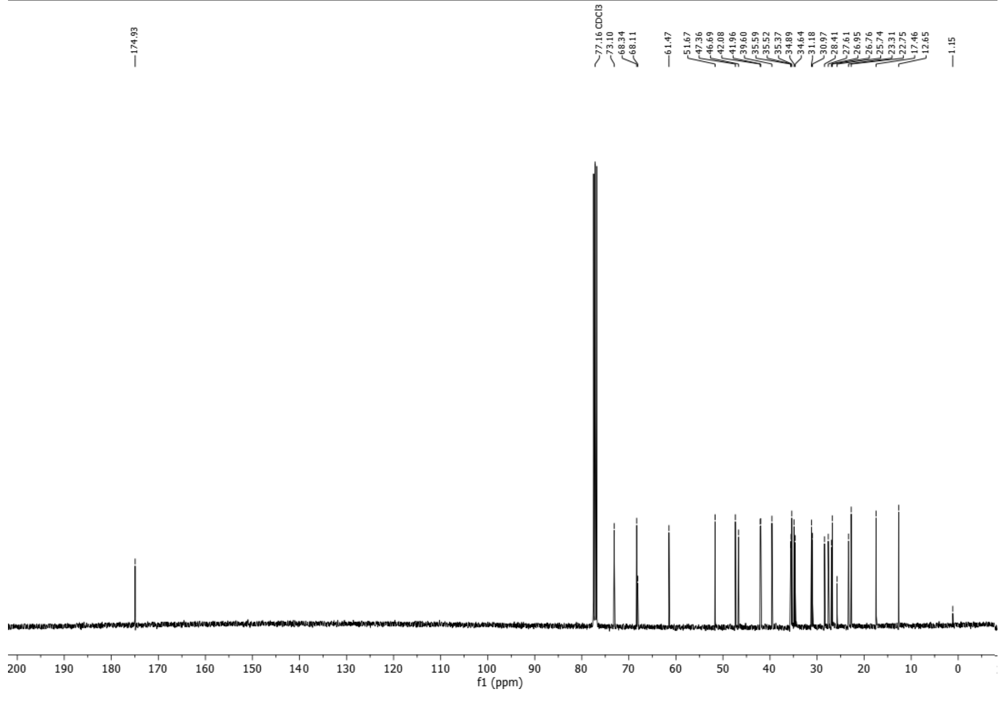

Methyl-3β-azido-7α-hydroxy-5β-cholan-24-oate (3b)

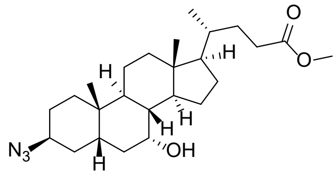

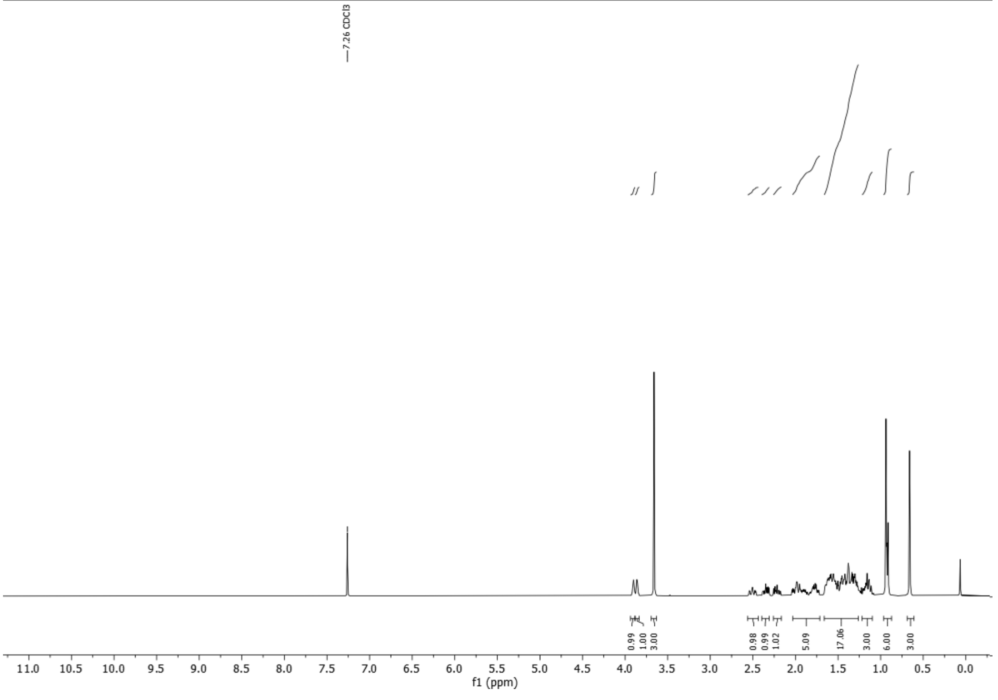

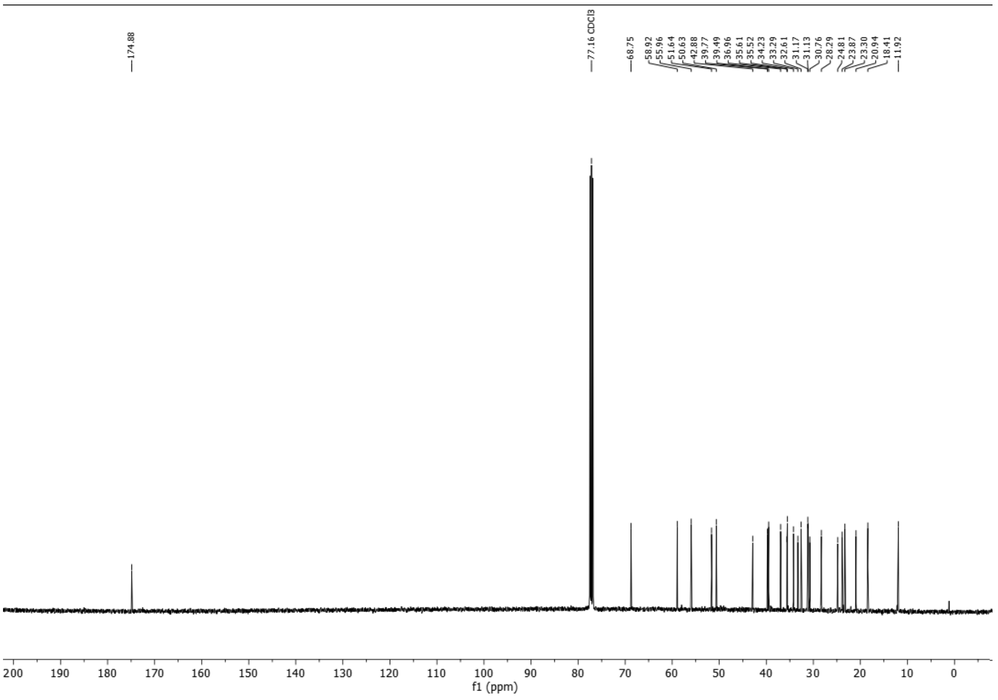

Methyl-3β-azido-12α-hydroxy-5β-cholan-24-oate (3c)

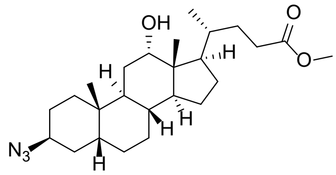

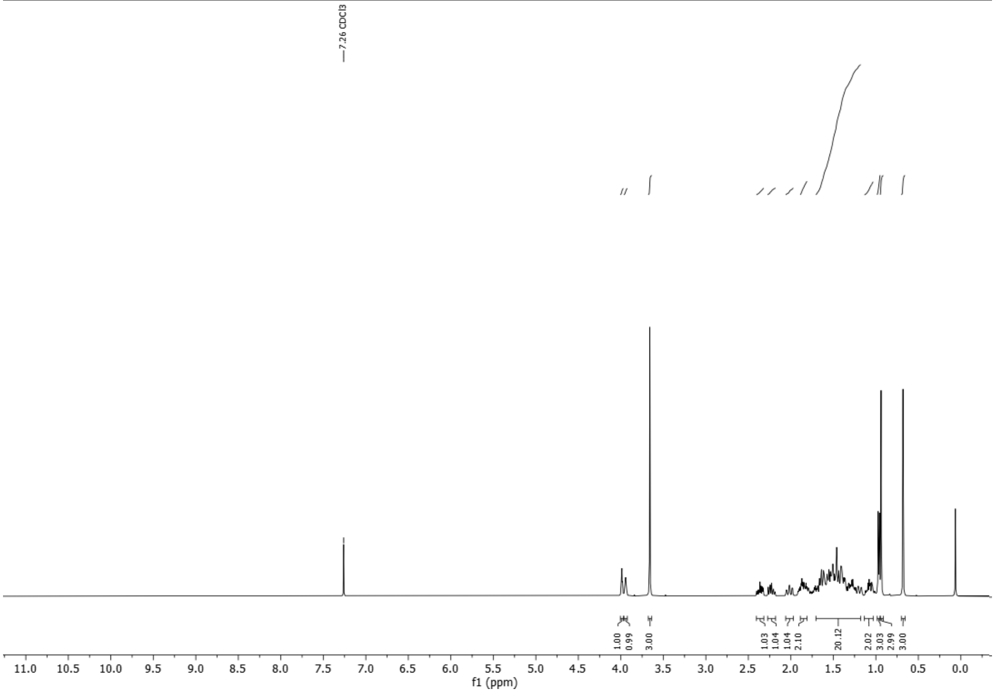

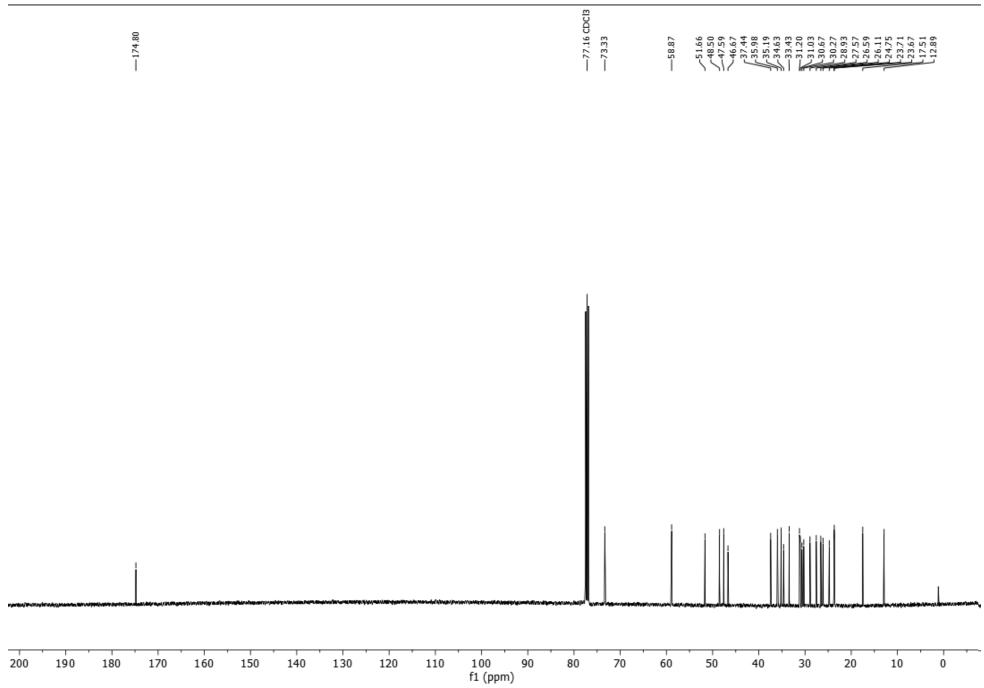

Amino BA Methyl Ester

Methyl-3β-amino-7α,12α-dihydroxy-5β-cholan-24-oate (4a)

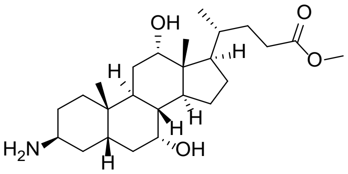

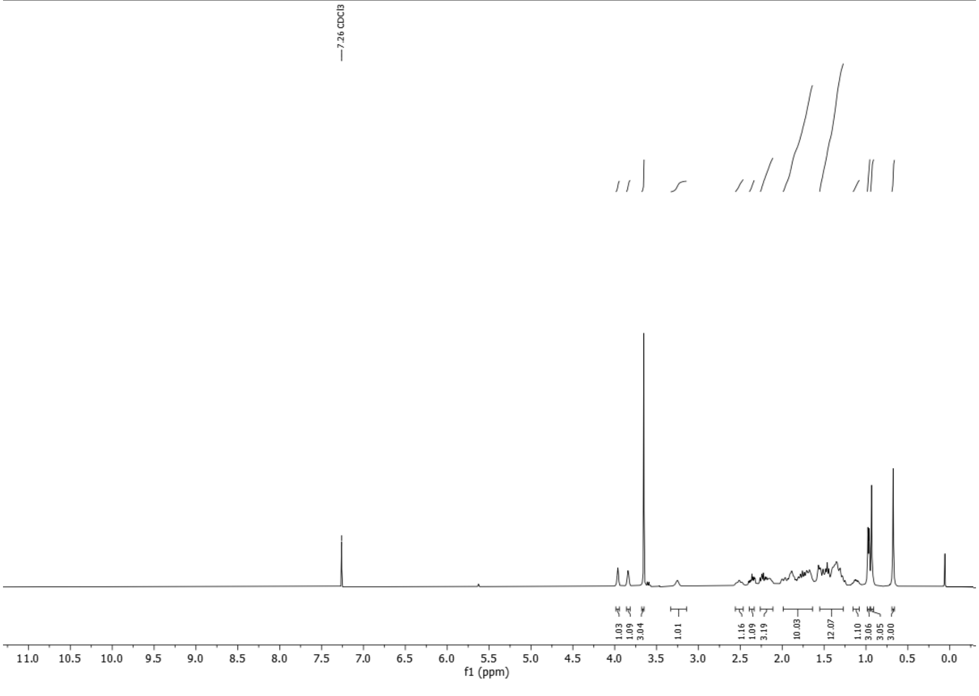

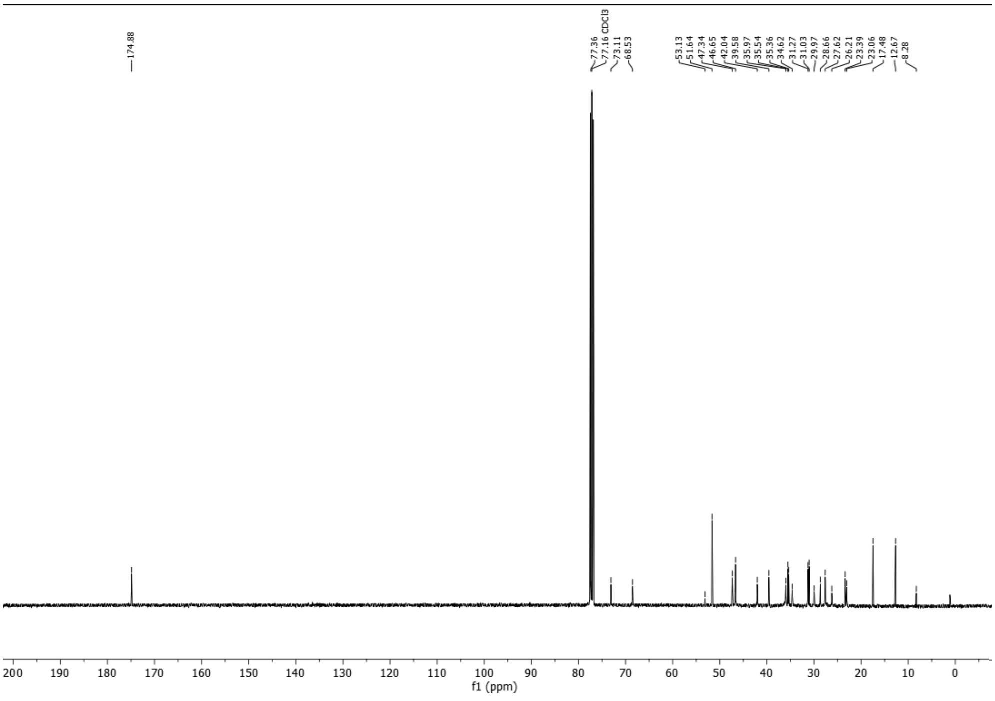

Methyl-3α-amino-7α,12α-dihydroxy-5β-cholan-24-oate (4d)

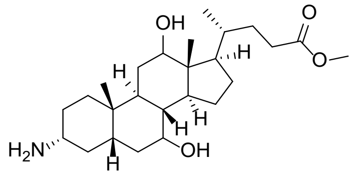

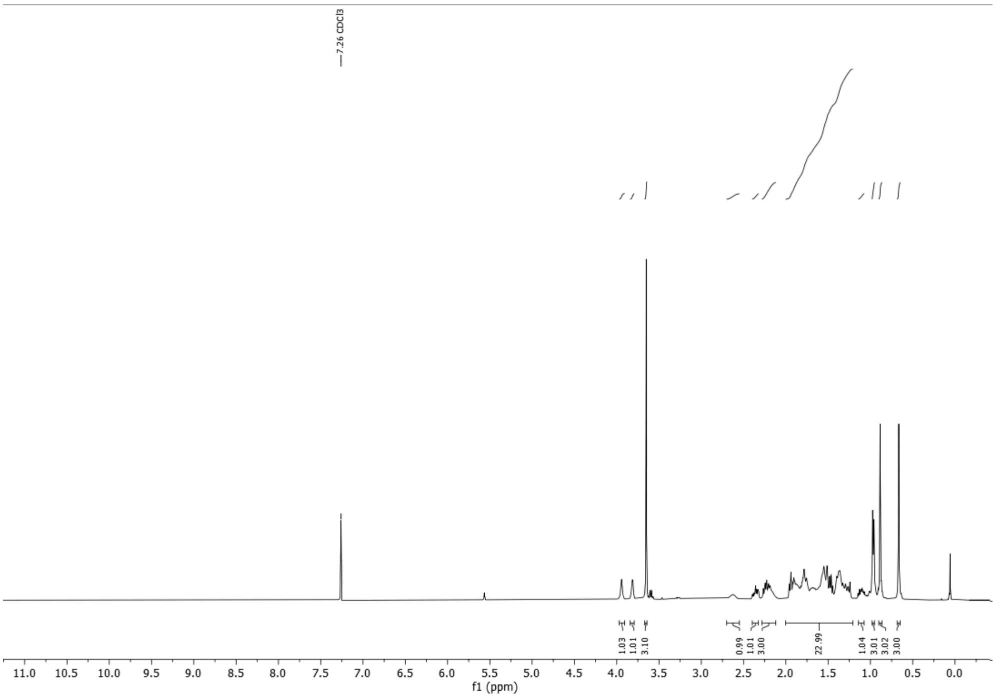

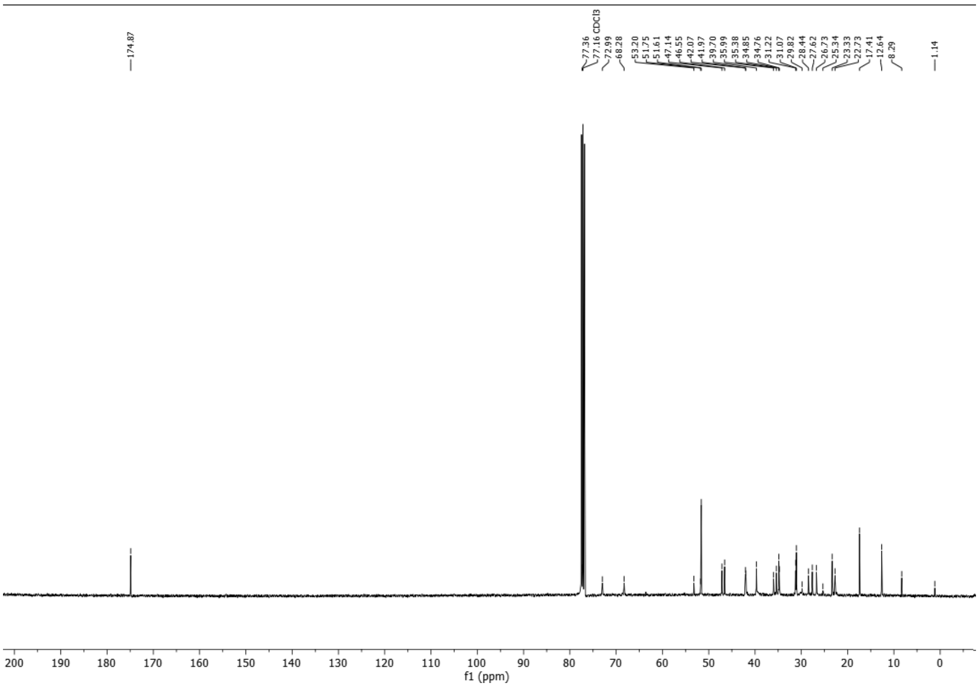

Methyl-3β-amino-7α-hydroxy-5β-cholan-24-oate (4b)

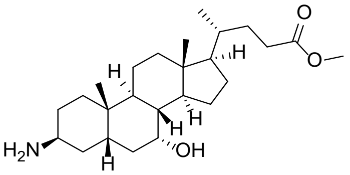

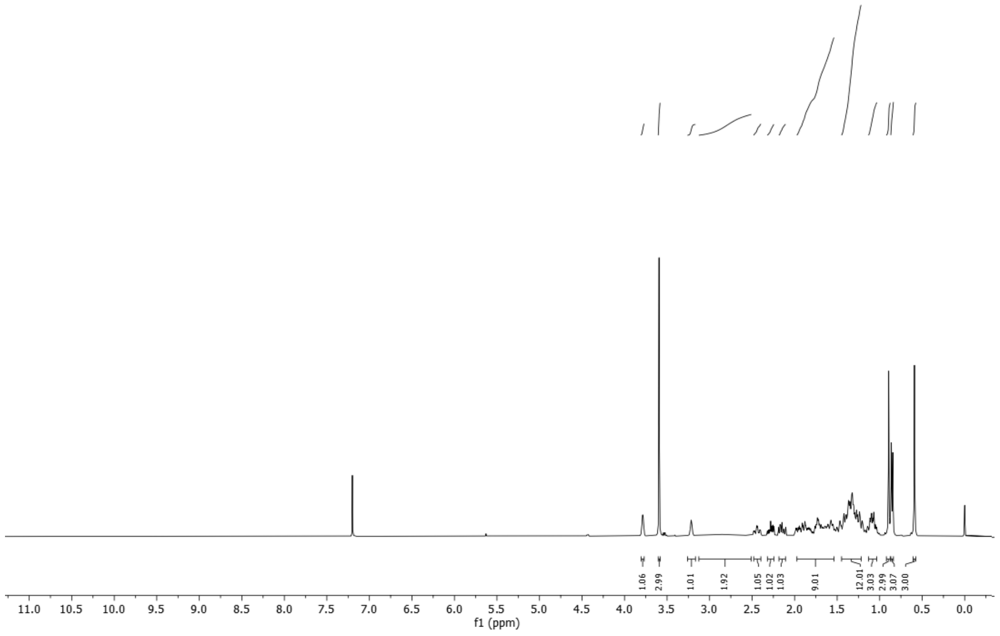

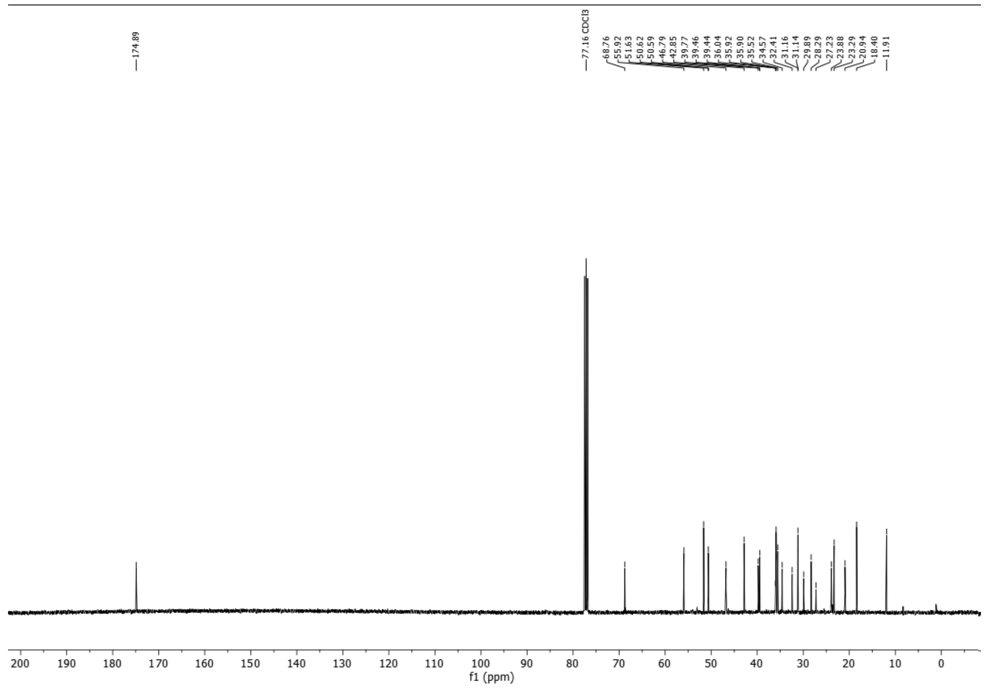

Methyl-3β-amino-12α-hydroxy-5β-cholan-24-oate (4c)

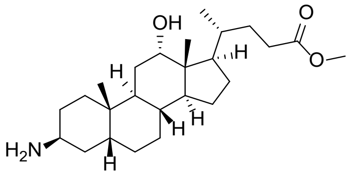

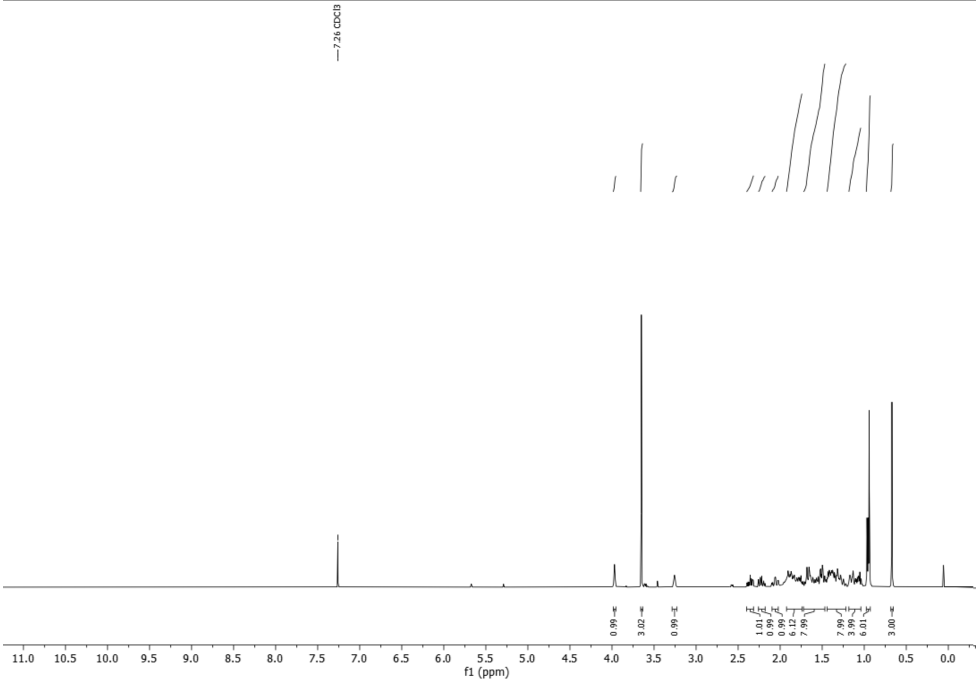

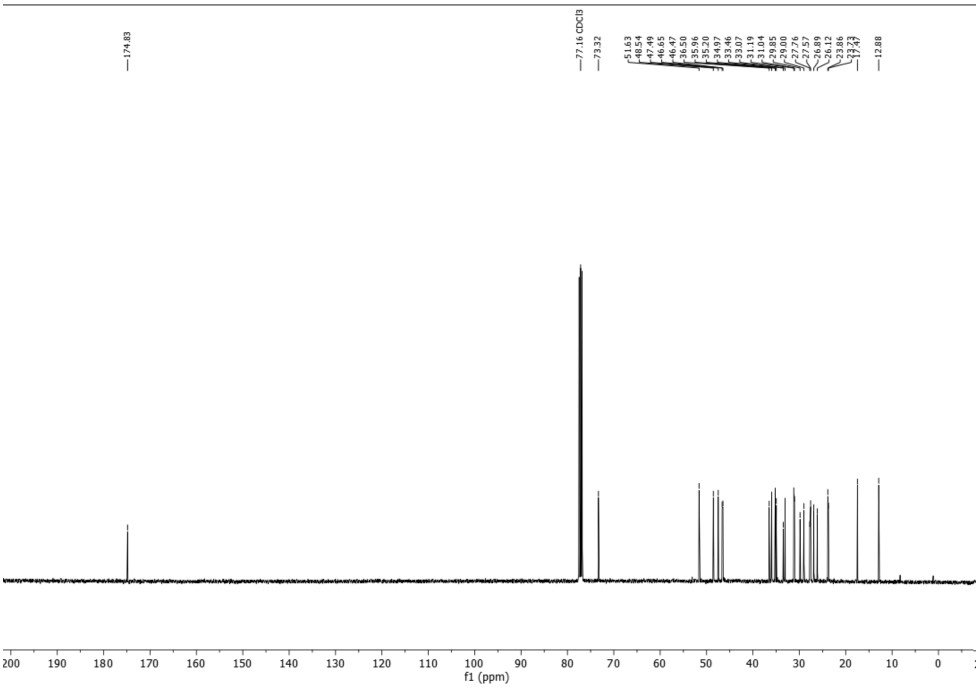

NBD BA Methyl Ester

Methyl-7α,12α-dihydroxy-3β-[(7-nitro-2,1,3-benzoxadiazol-4-yl)amino]-5β-cholan-24-oate (5a)

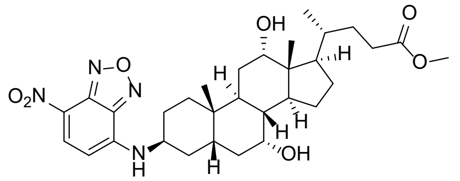

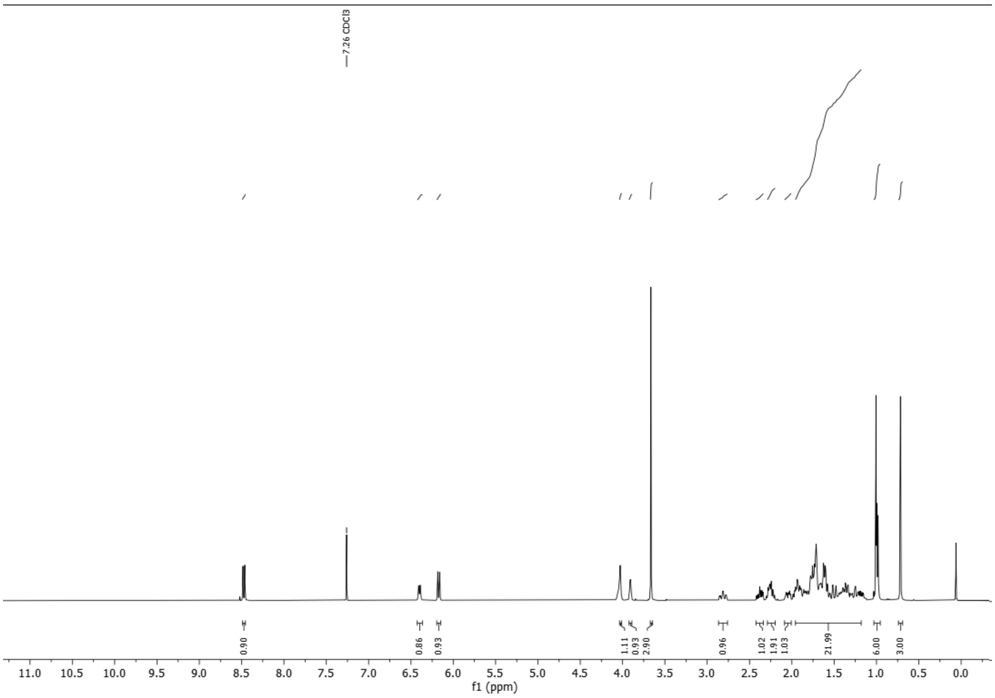

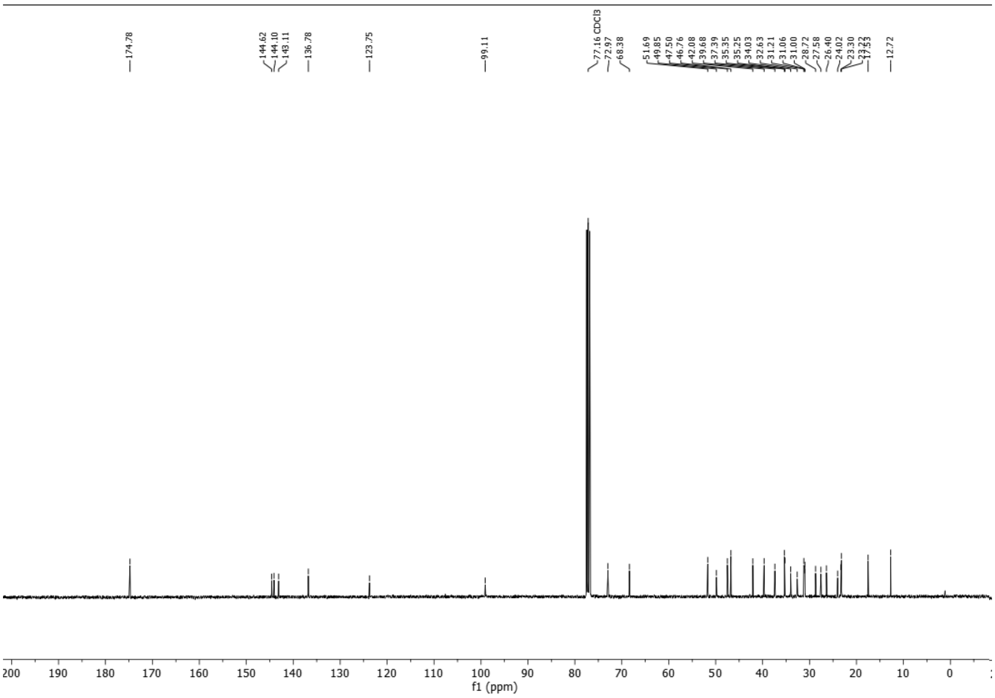

Methyl-7α,12α-dihydroxy-3α-[(7-nitro-2,1,3-benzoxadiazol-4-yl)amino]-5β-cholan-24-oate (5d)

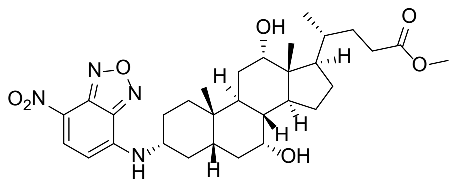

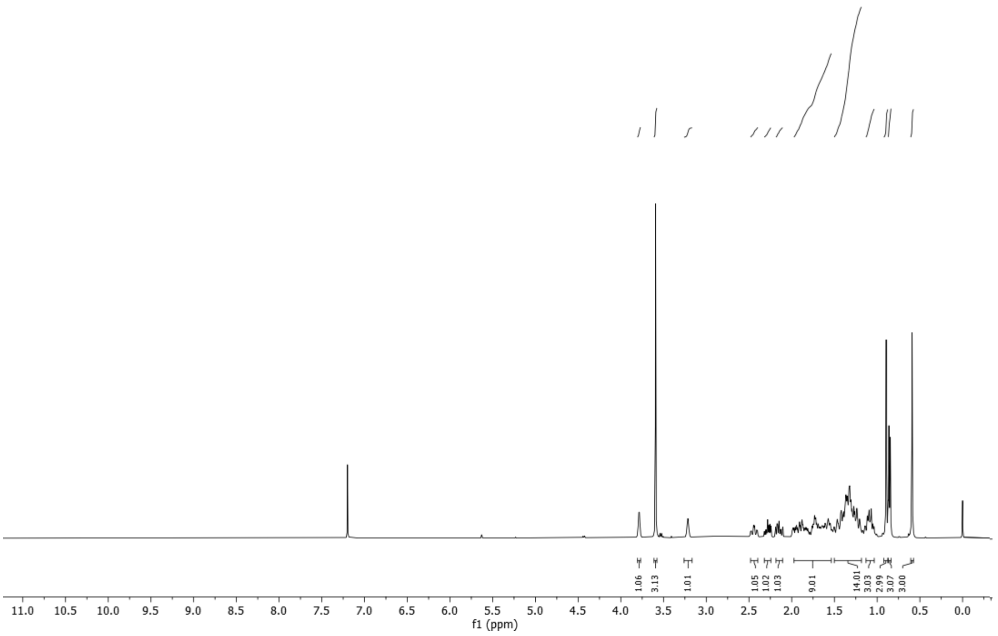

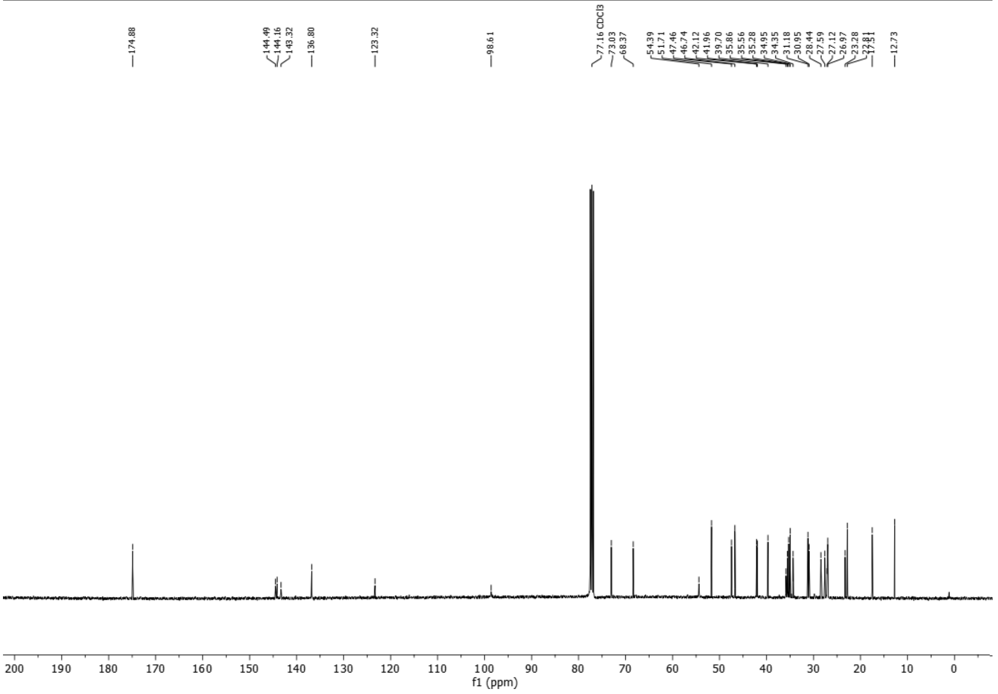

Methyl-7α-hydroxy-3β-[(7-nitro-2,1,3-benzoxadiazol-4-yl)amino]-5β-cholan-24-oate (5b)

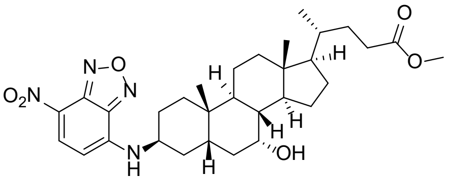

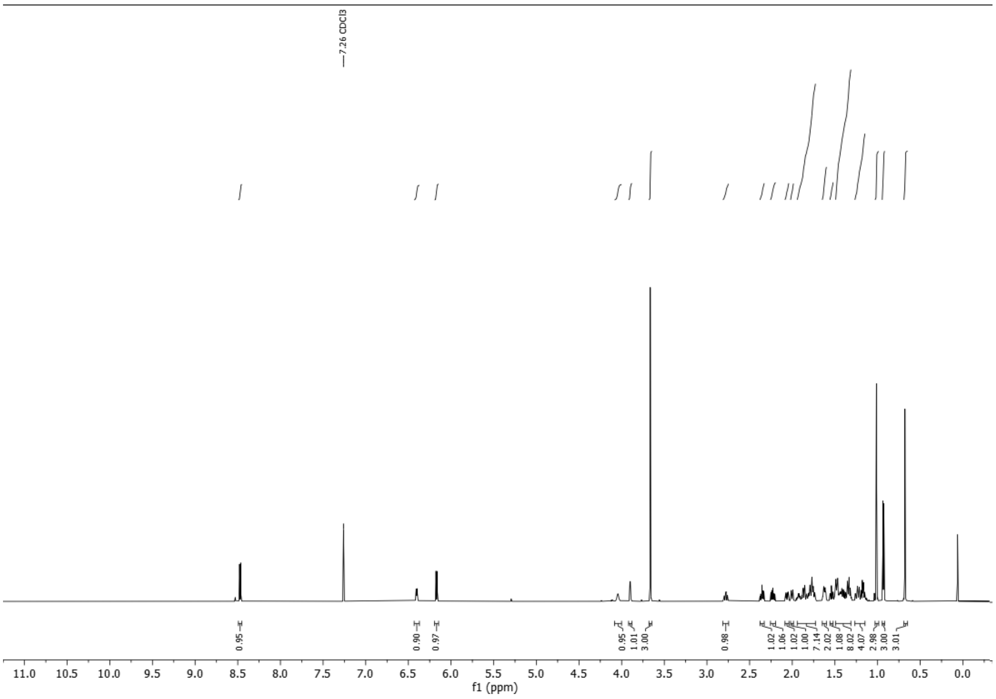

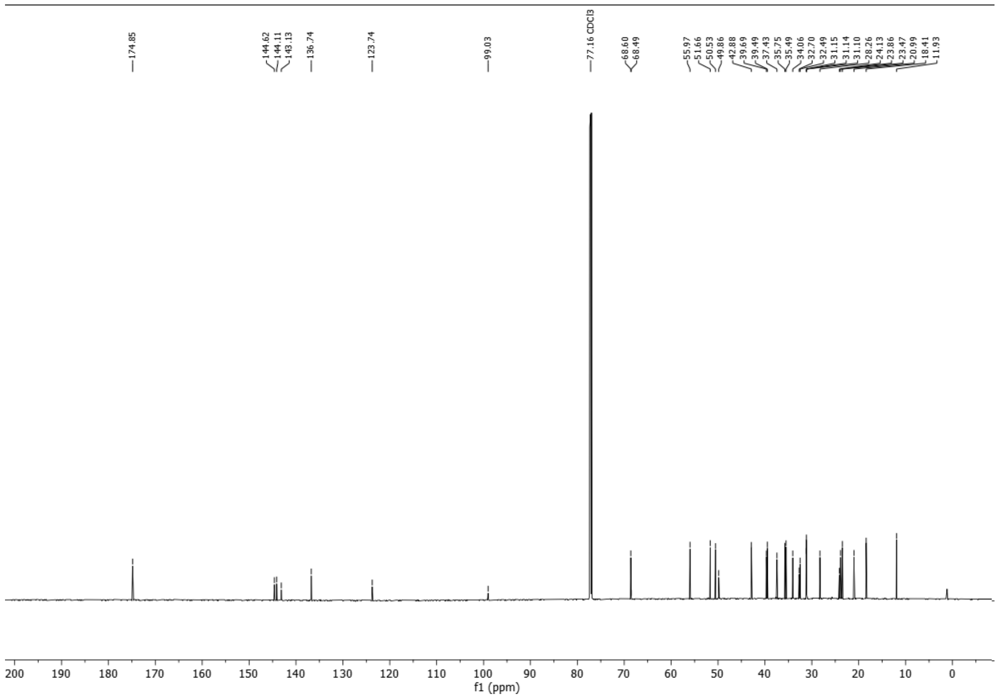

Methyl-12α-hydroxy-3β-[(7-nitro-2,1,3-benzoxadiazol-4-yl)amino]-5β-cholan-24-oate (5c)

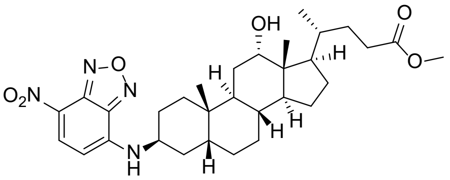

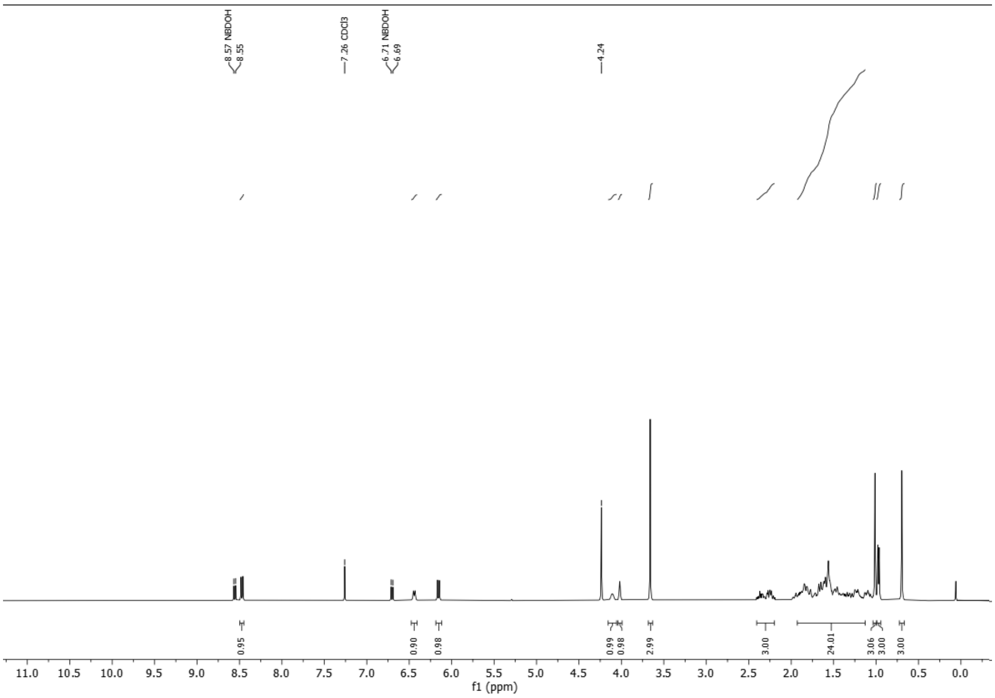

NBD BA

7α,12α-Dihydroxy-3β-[(7-nitro-2,1,3-benzoxadiazol-4-yl)amino]-5β-cholan-24-oate (6a)

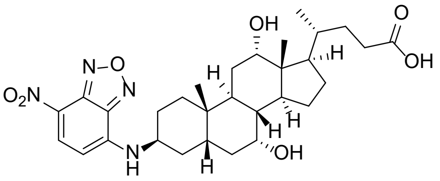

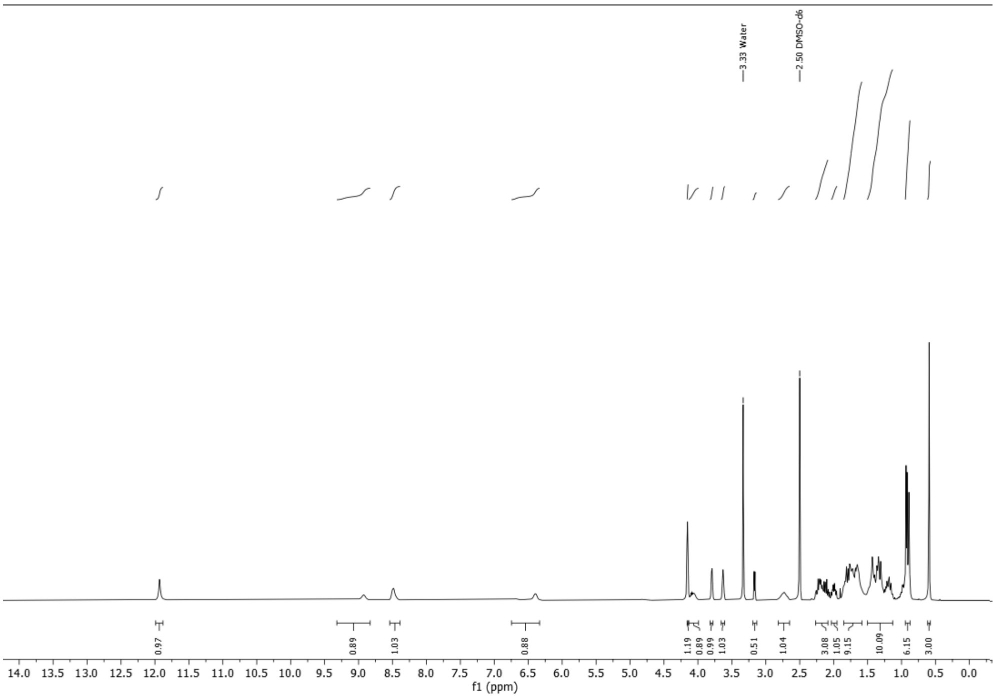

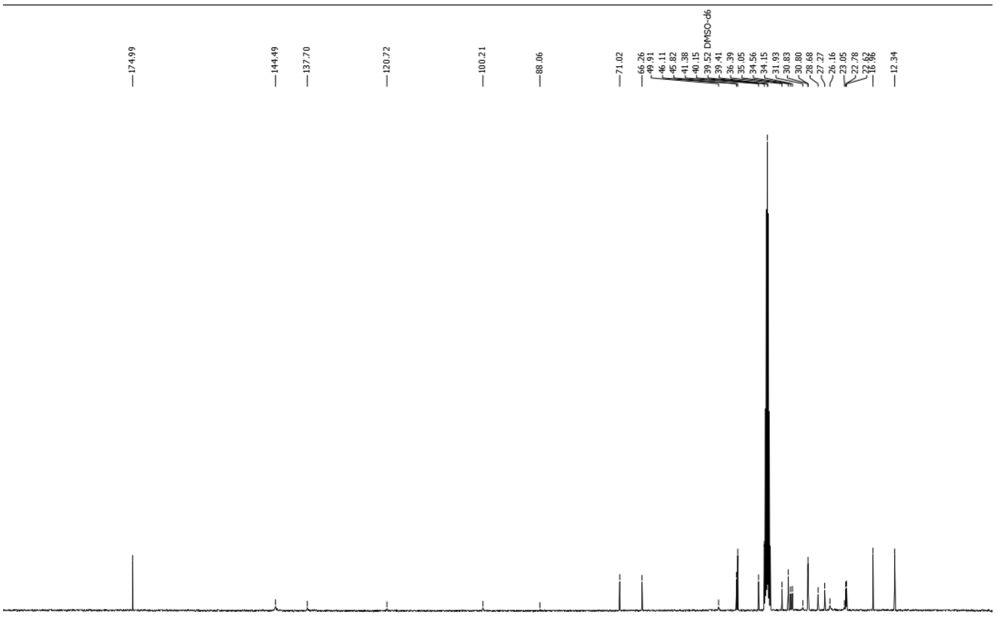

7α,12α-Dihydroxy-3α-[(7-nitro-2,1,3-benzoxadiazol-4-yl)amino]-5β-cholan-24-oate (6d)

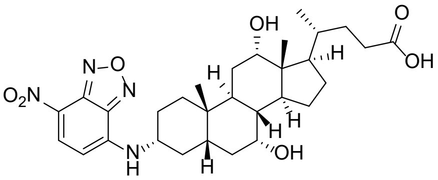

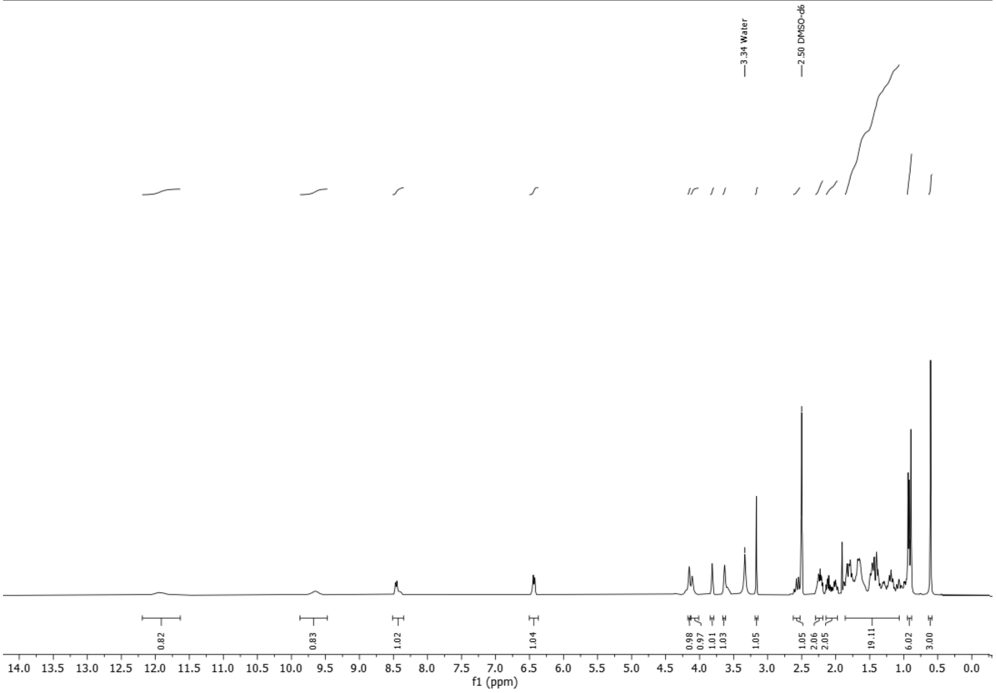

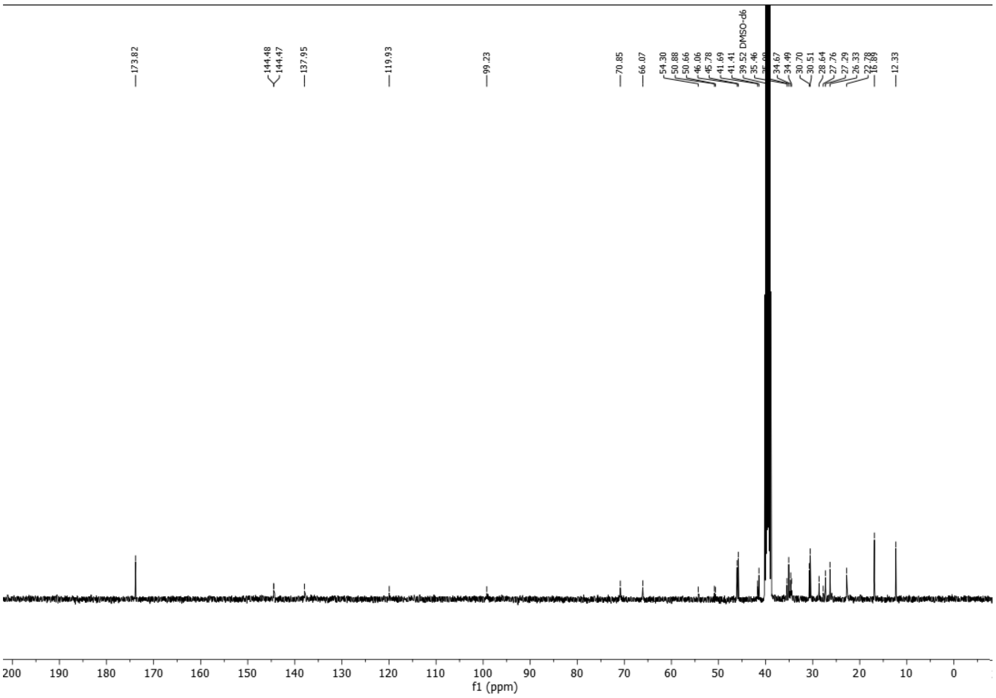

7α-Hydroxy-3β-[(7-nitro-2,1,3-benzoxadiazol-4-yl)amino]-5β-cholan-24-oate (6b)

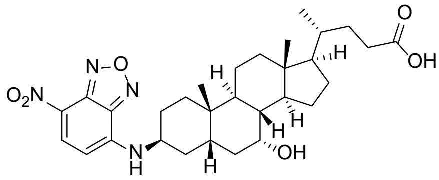

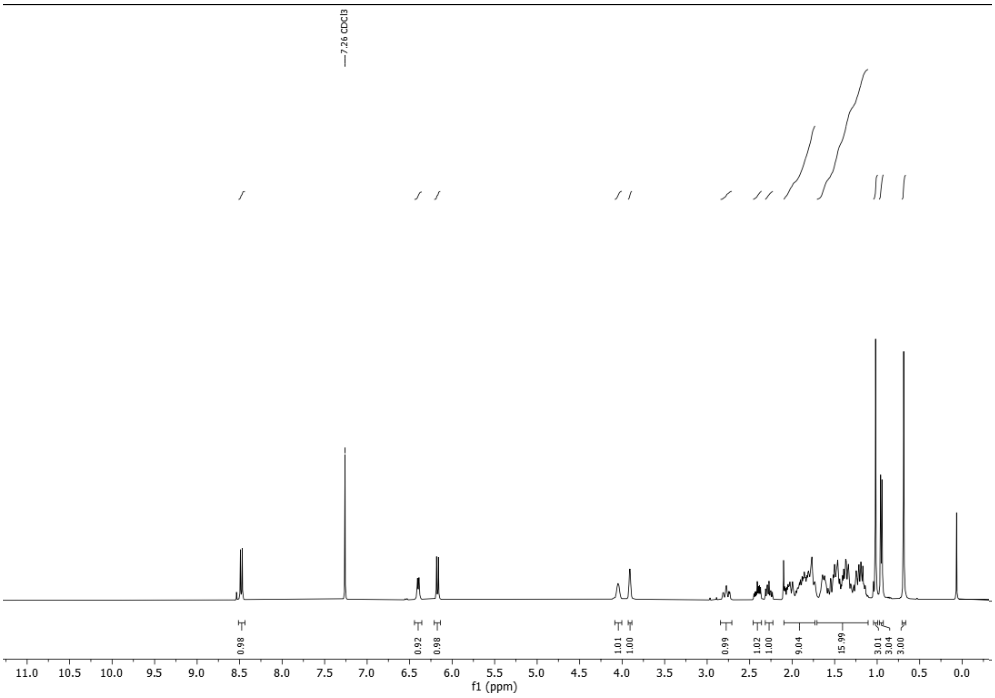

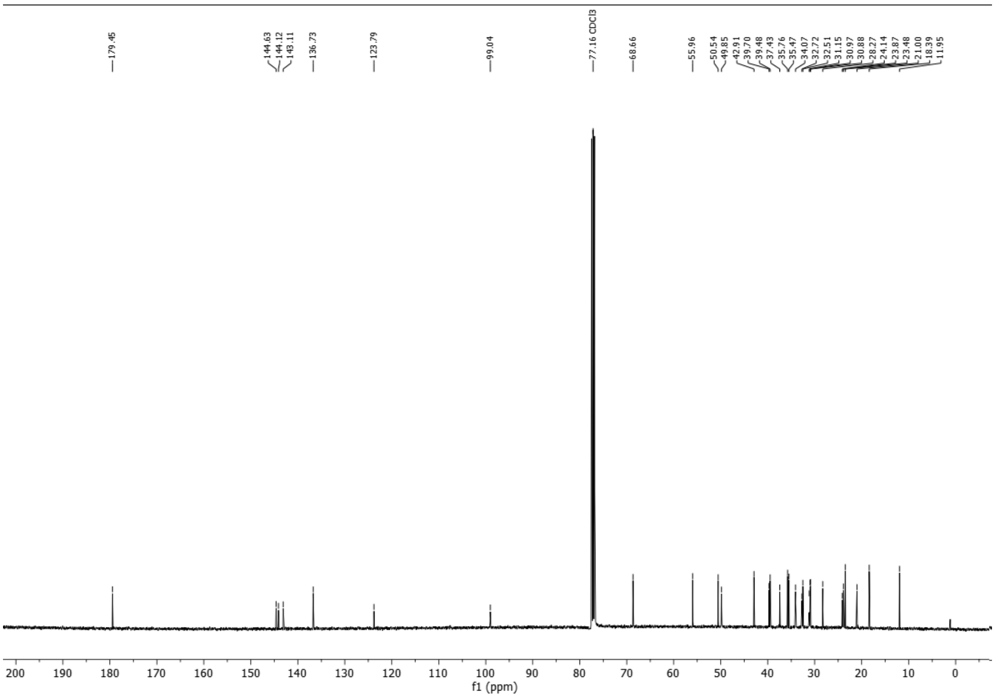

12α-Hydroxy-3β-[(7-nitro-2,1,3-benzoxadiazol-4-yl)amino]-5β-cholan-24-oate (6c)

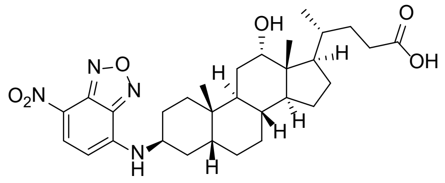

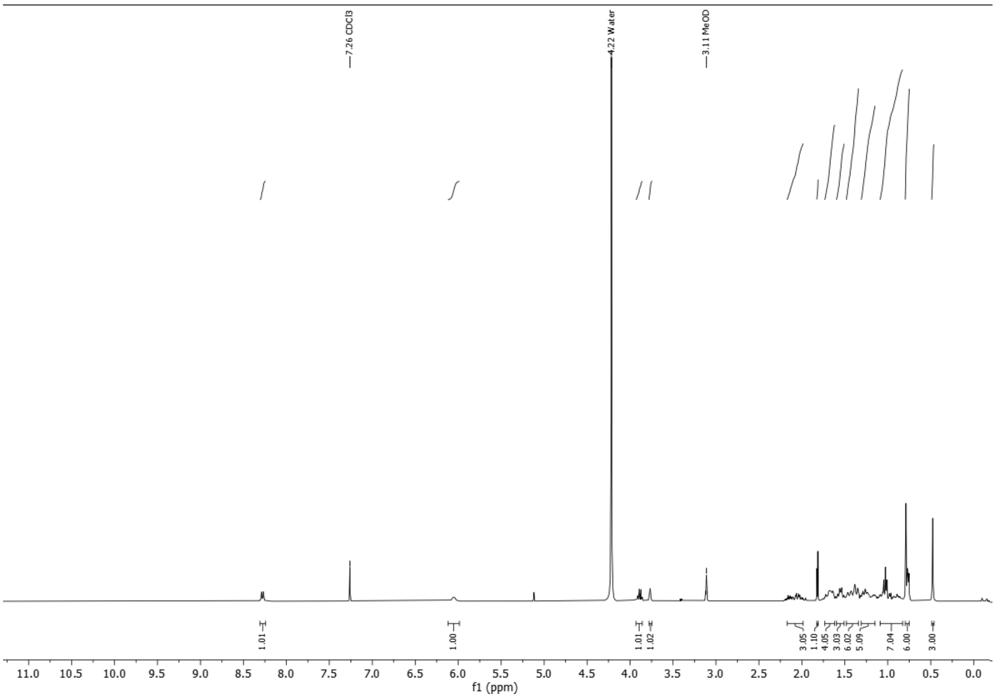

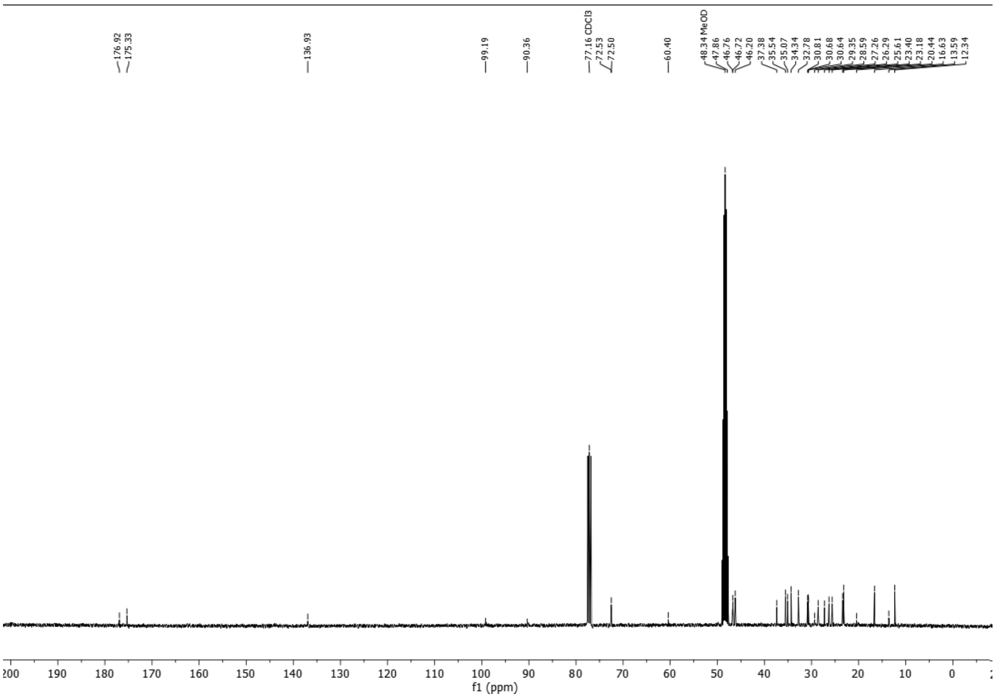

Tetrabutylammonium-2-[(*tert*-butoxycarbonyl)amino]ethane sulfonic acid (10)

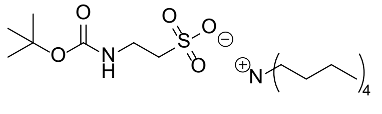

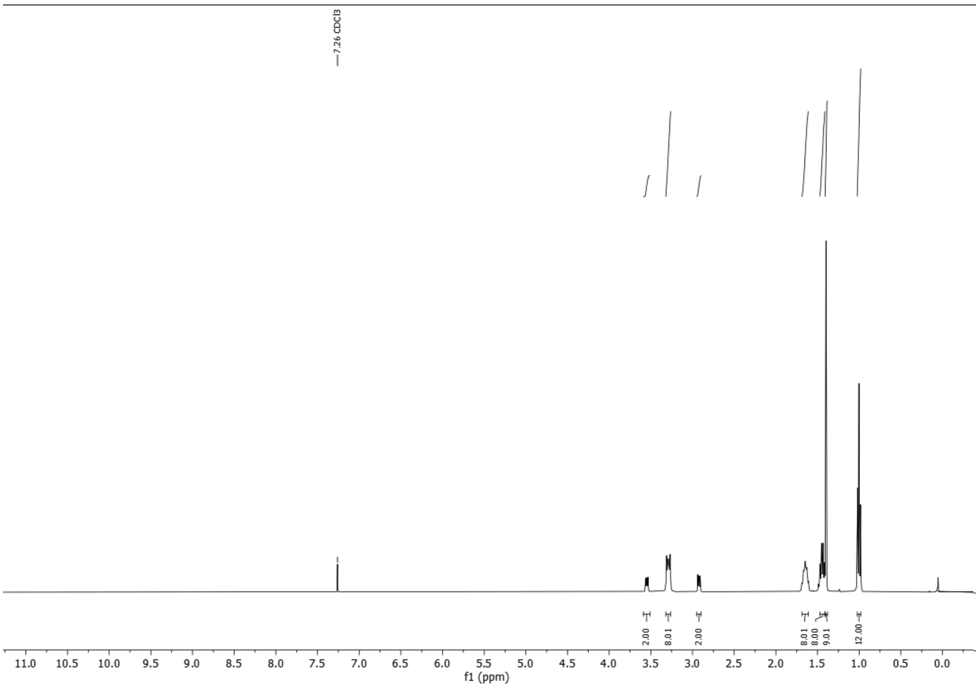

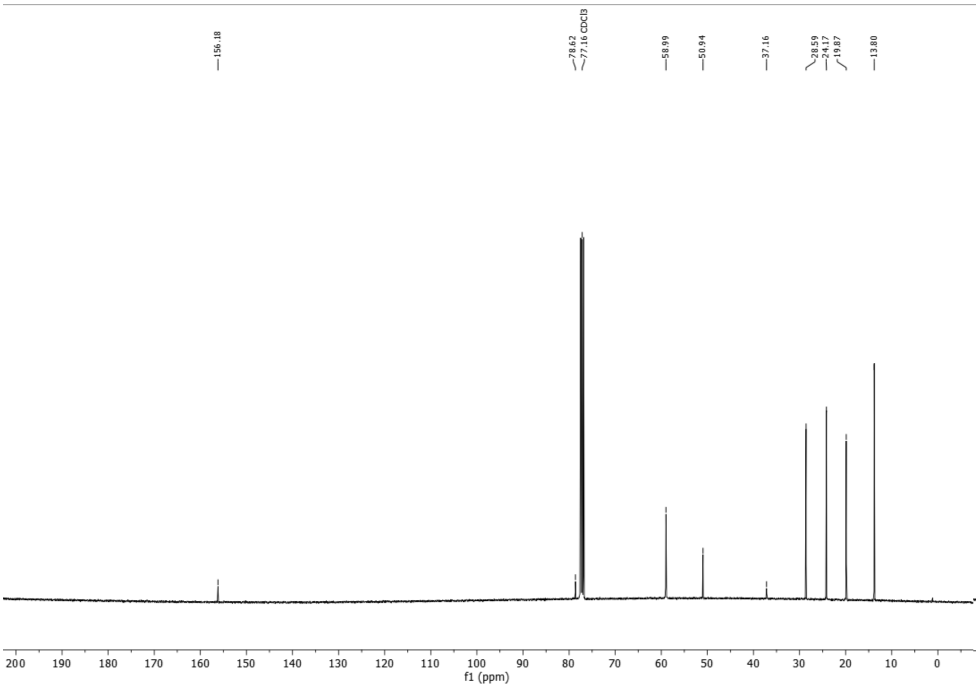

2,2,2-Trifluorethyl-2-[[1,1-dimethylethoxy)carbonyl]amino]ethane-sulfonate (11)

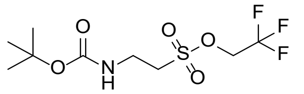

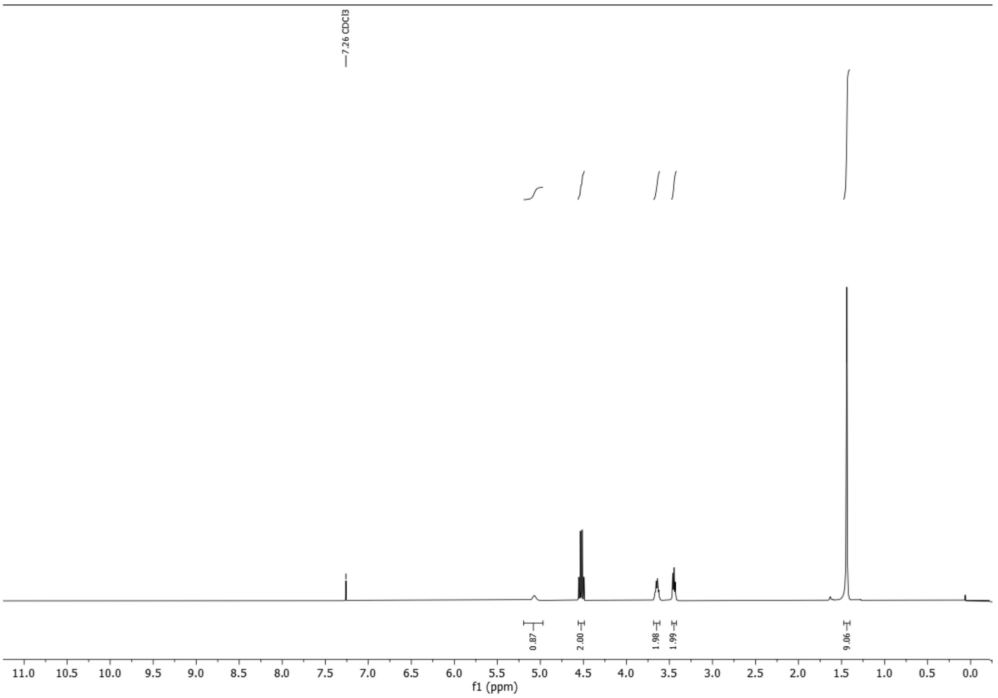

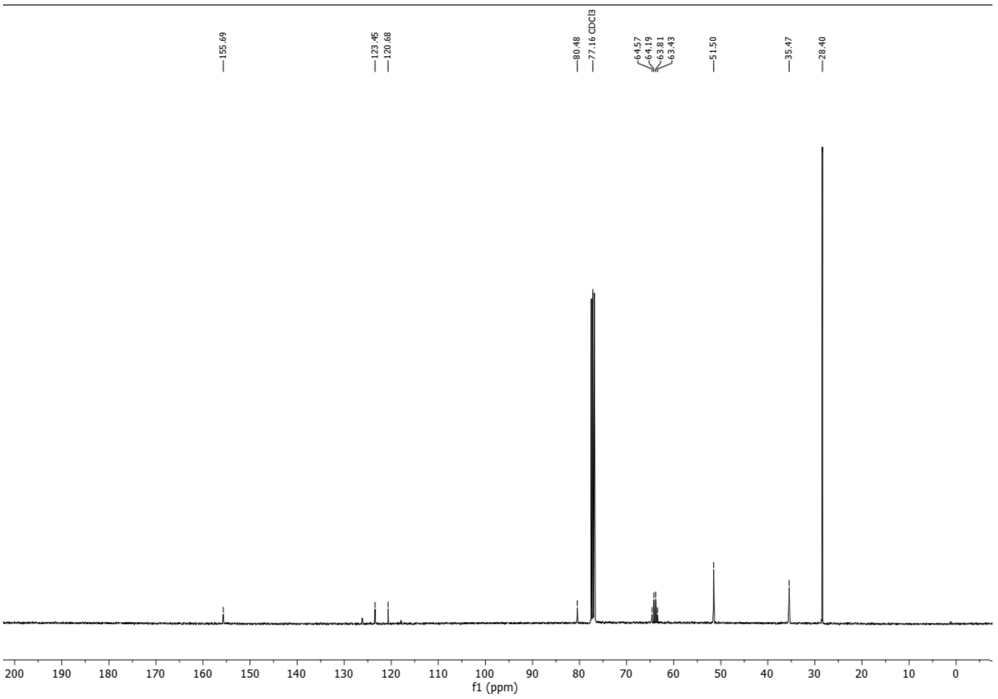

2,2,2-Trifluorethyl-2-aminoethane sulfonate (12)

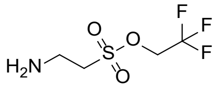

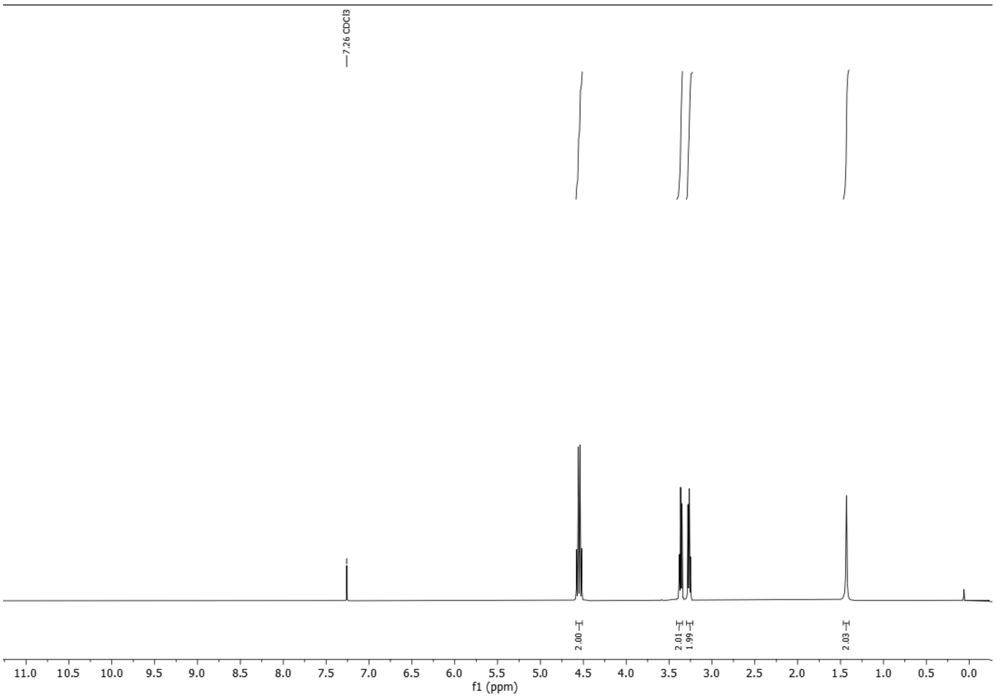

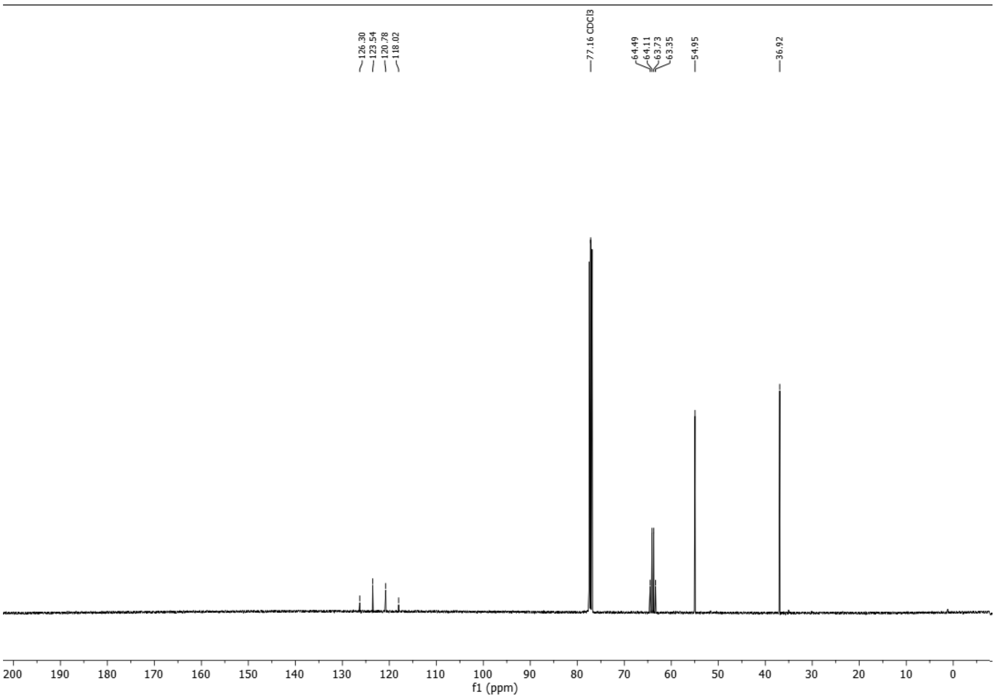

Trifluoroethanol-protected NBD T-BA

7α,12α-Dihydroxy-3β-[(7-nitro-2,1,3-benzoxadiazol-4-yl)amino]-5β-oxocholane-24-yl]amino] ethane trifluoroethane sulfonic acid ester (13a)

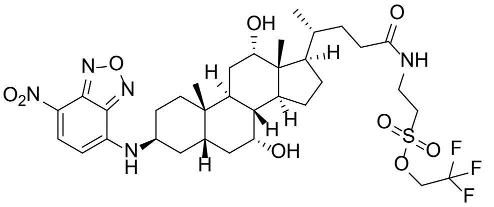

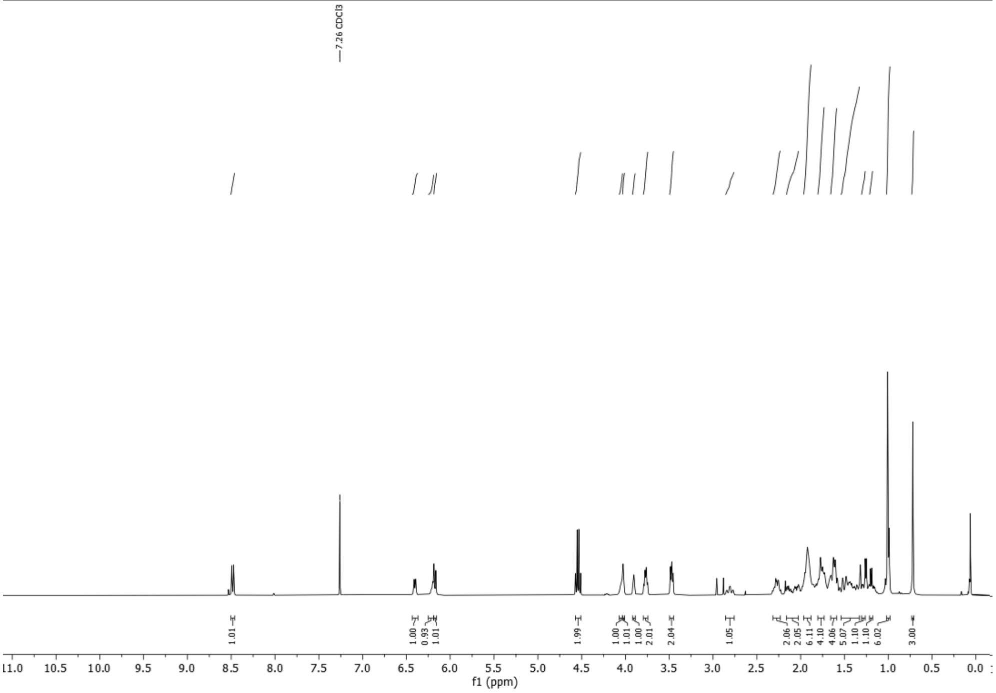

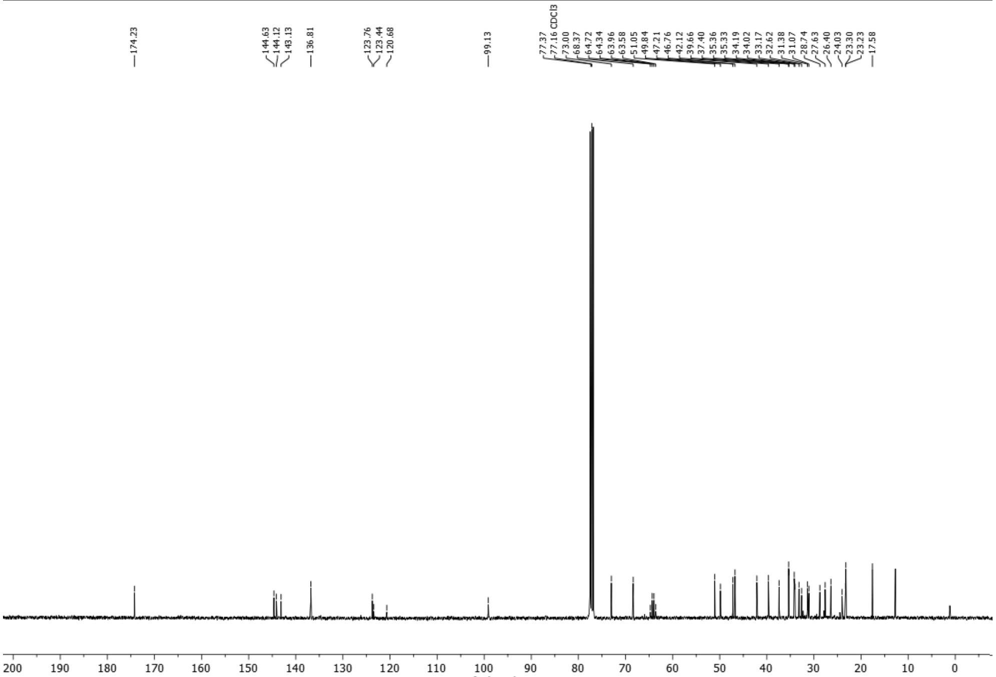

7α,12α-Dihydroxy-3α-[(7-nitro-2,1,3-benzoxadiazol-4-yl)amino]-5β-oxocholane-24-yl]amino] ethane trifluoroethane sulfonic acid ester (13d)

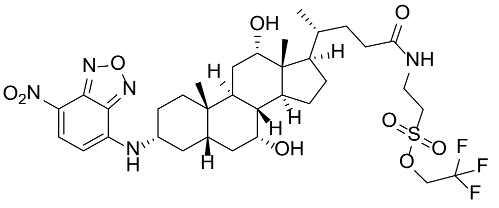

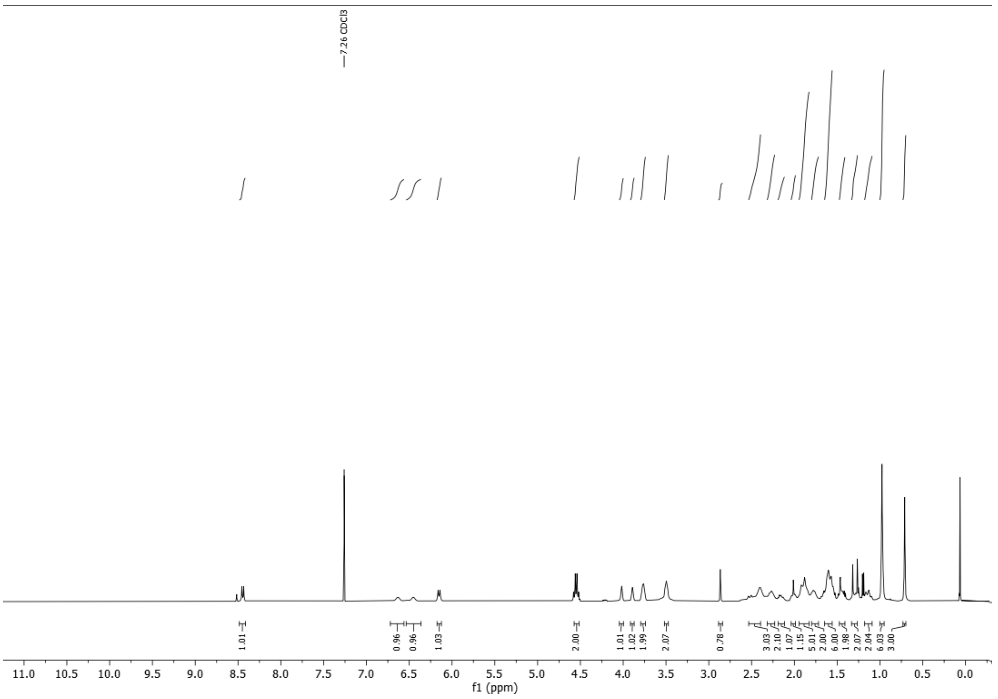

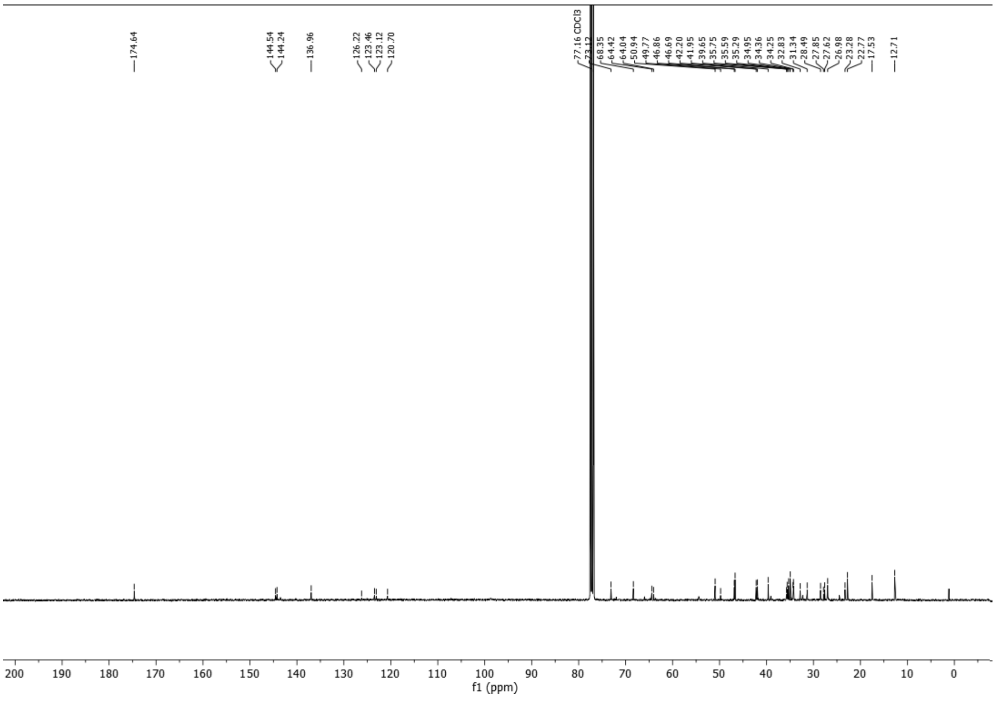

7α-Hydroxy-3β-[(7-nitro-2,1,3-benzoxadiazol-4-yl)amino]-5β-oxocholane-24-yl]amino] ethane trifluoroethane sulfonic acid ester (13b)

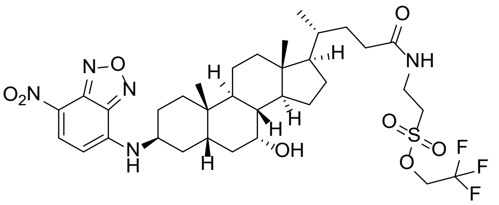

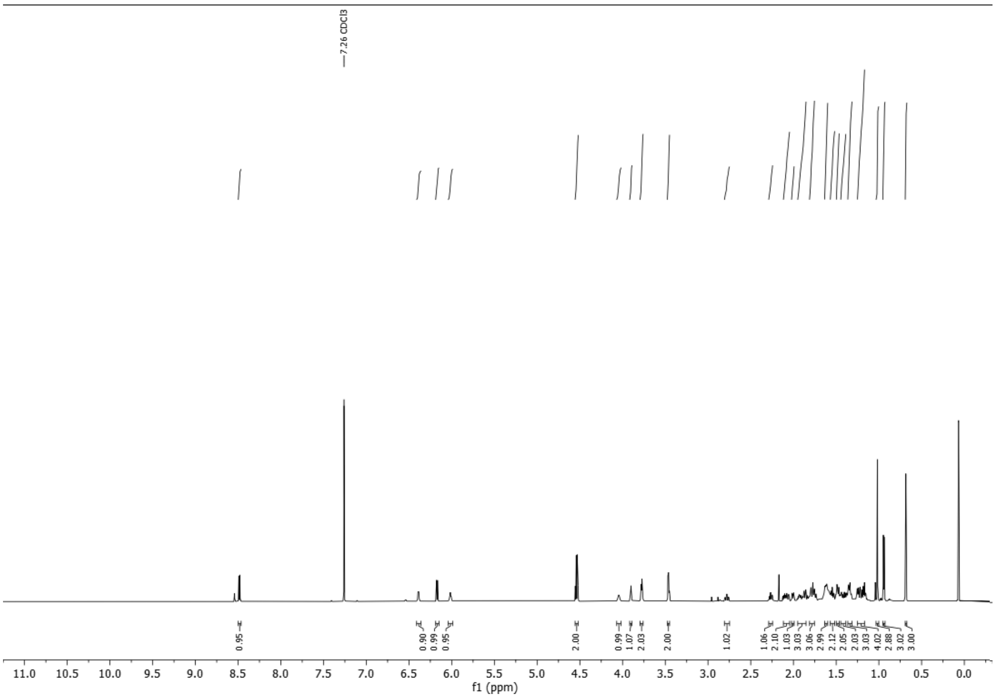

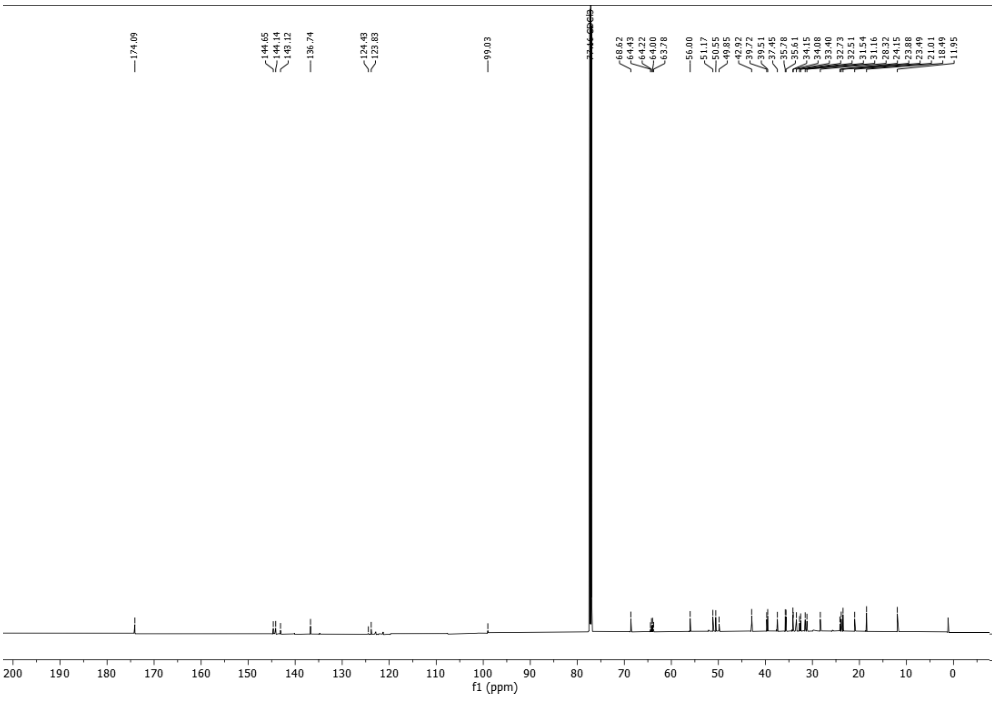

NBD T-BS

7α,12α-Dihydroxy-3β-[(7-nitro-2,1,3-benzoxadiazol-4-yl)amino]-5β-oxocholan-24yl]amino]ethane sulfonic acid (14a)

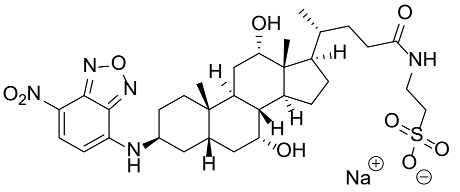

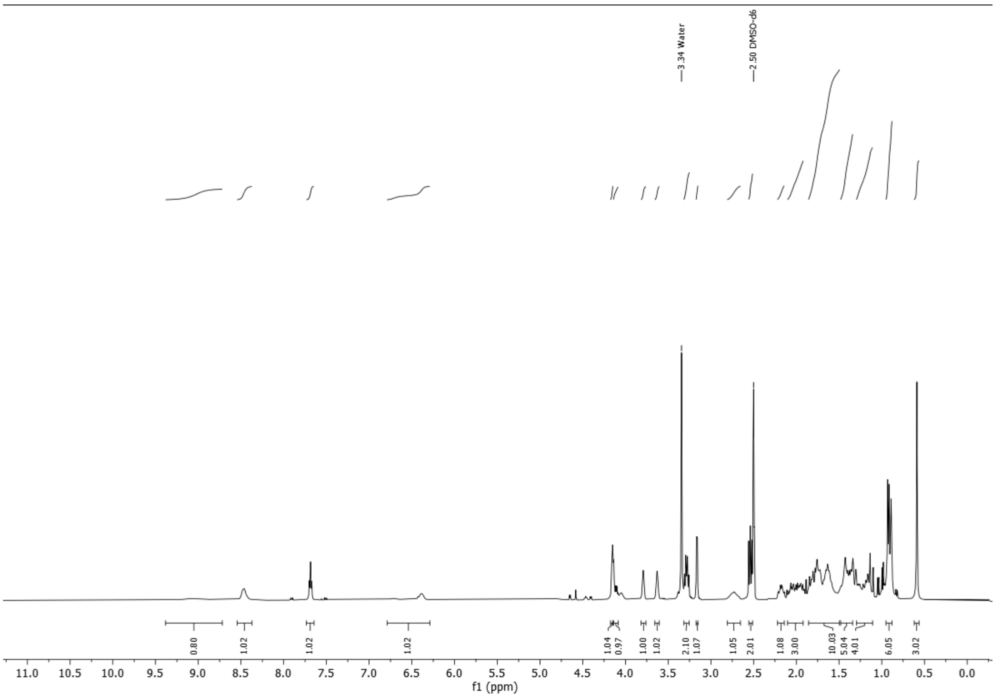

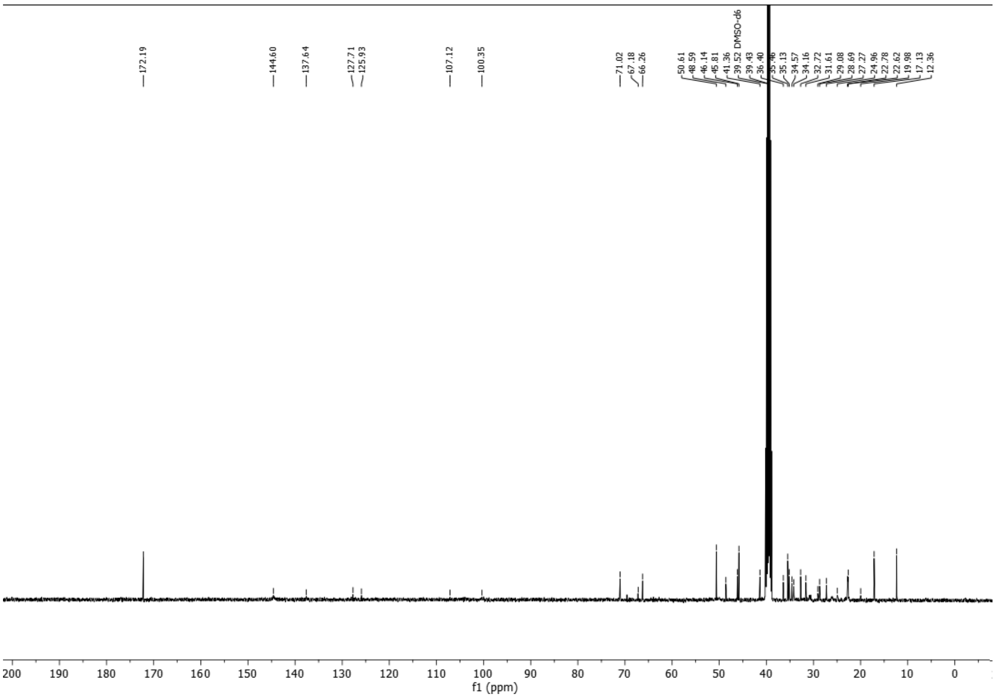

7α,12α-Dihydroxy-3α-[(7-nitro-2,1,3-benzoxadiazol-4-yl)amino]-5β-oxocholan-24-yl]amino]ethane sulfonic acid (14d)

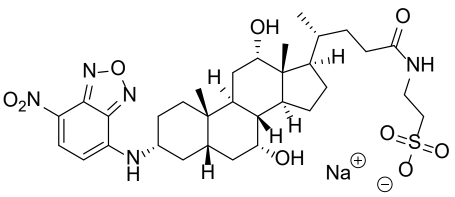

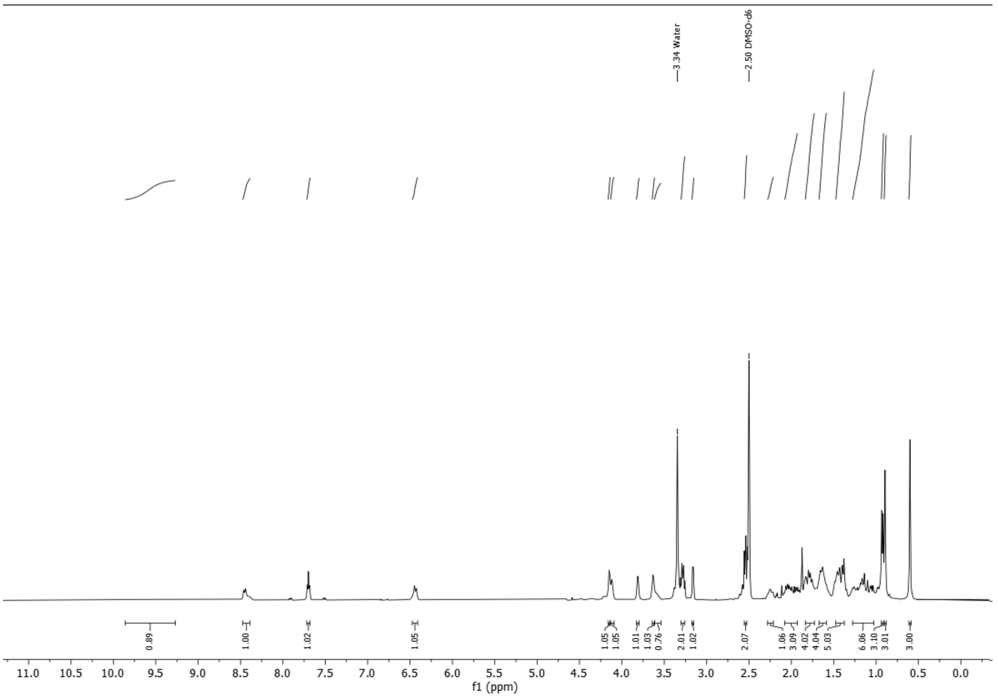

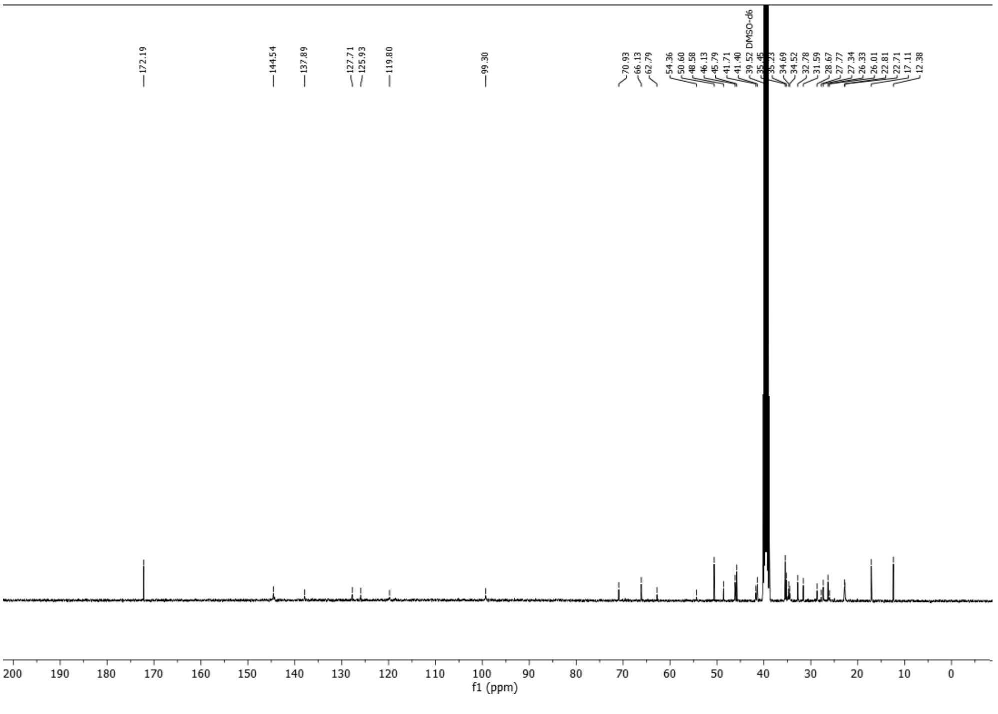

7α-Hydroxy-3β-[(7-nitro-2,1,3-benzoxadiazol-4-yl)amino]-5β-oxocholan-24-yl]amino]ethane sulfonic acid (14b)

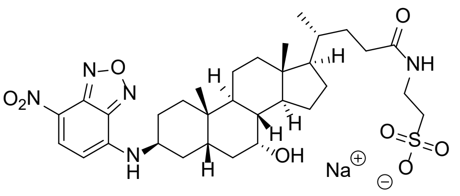

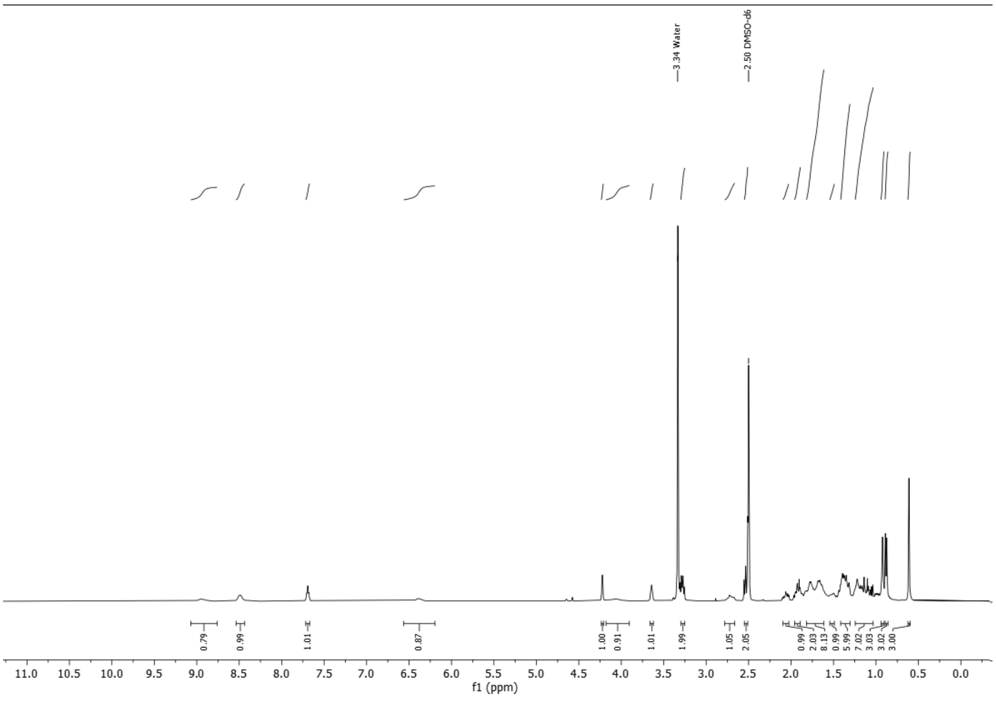

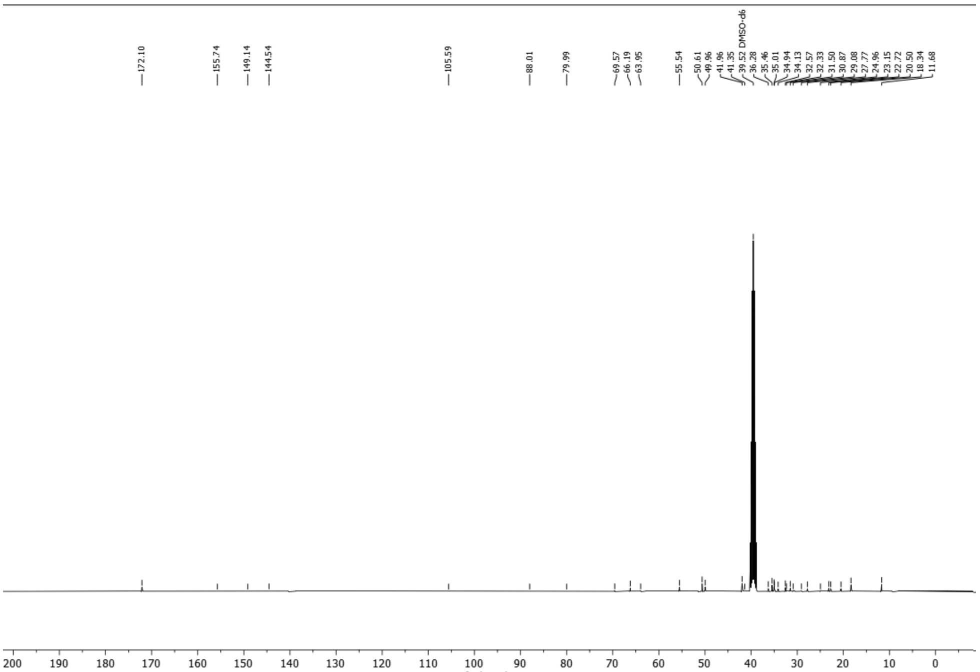

NBD G-BA Methyl Ester

7α,12α-Dihydroxy-3β-[(7-nitro-2,1,3-benzoxadiazol-4-yl)amino]-5β-oxocholan-24-yl]glycine methyl ester (15a)

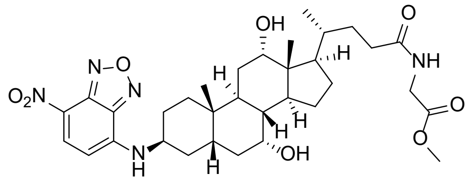

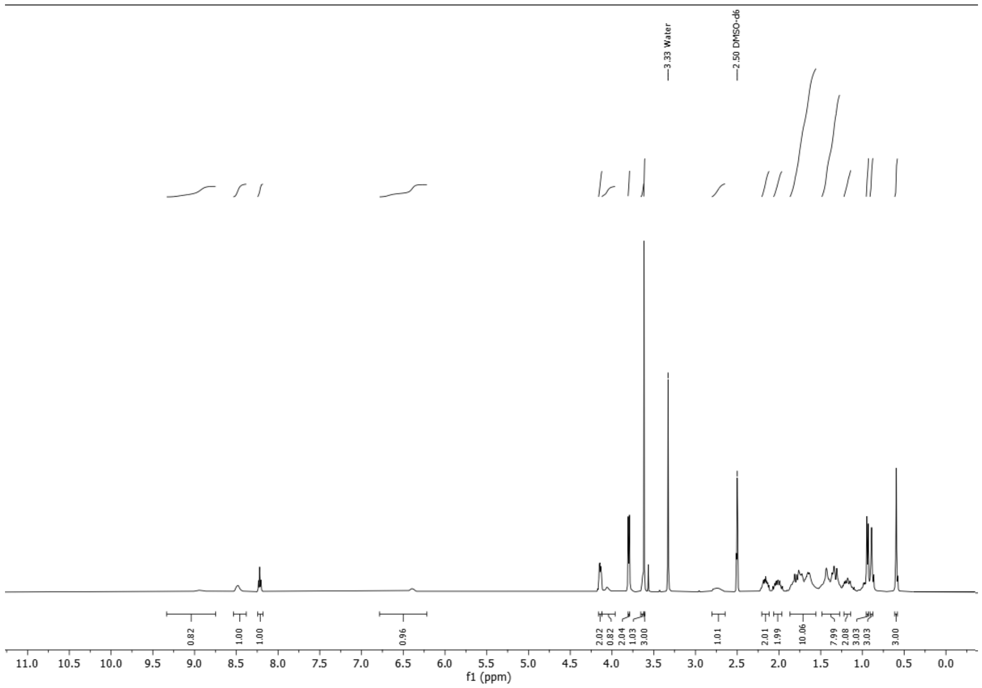

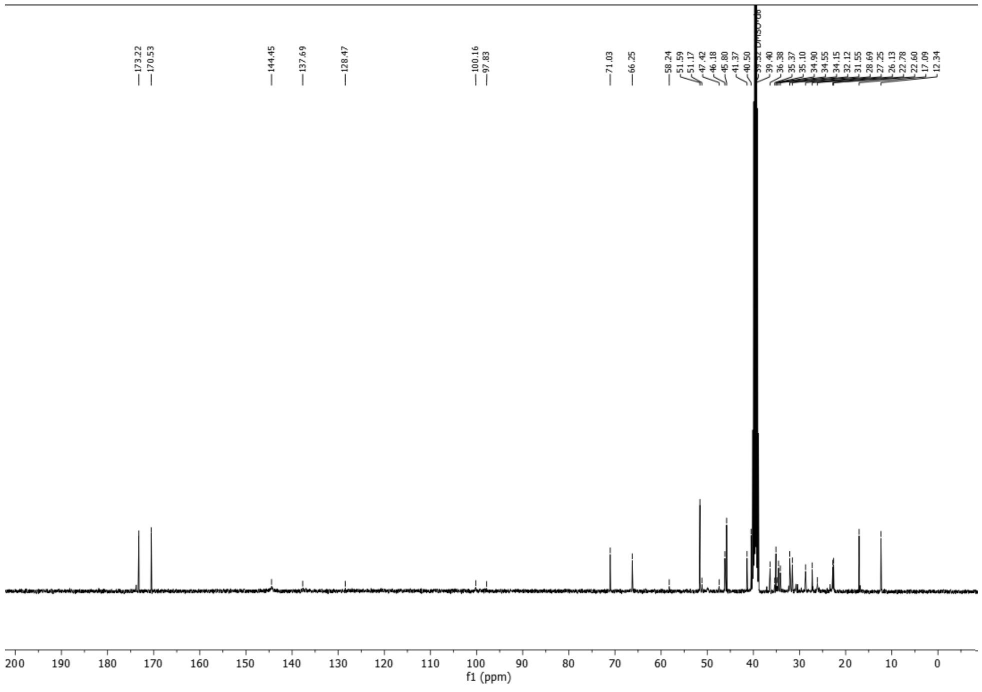

7α,12α-Dihydroxy-3α-[(7-nitro-2,1,3-benzoxadiazol-4-yl)amino]-5β-oxocholan-24-yl]glycine methyl ester (15d)

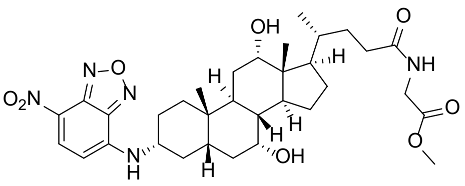

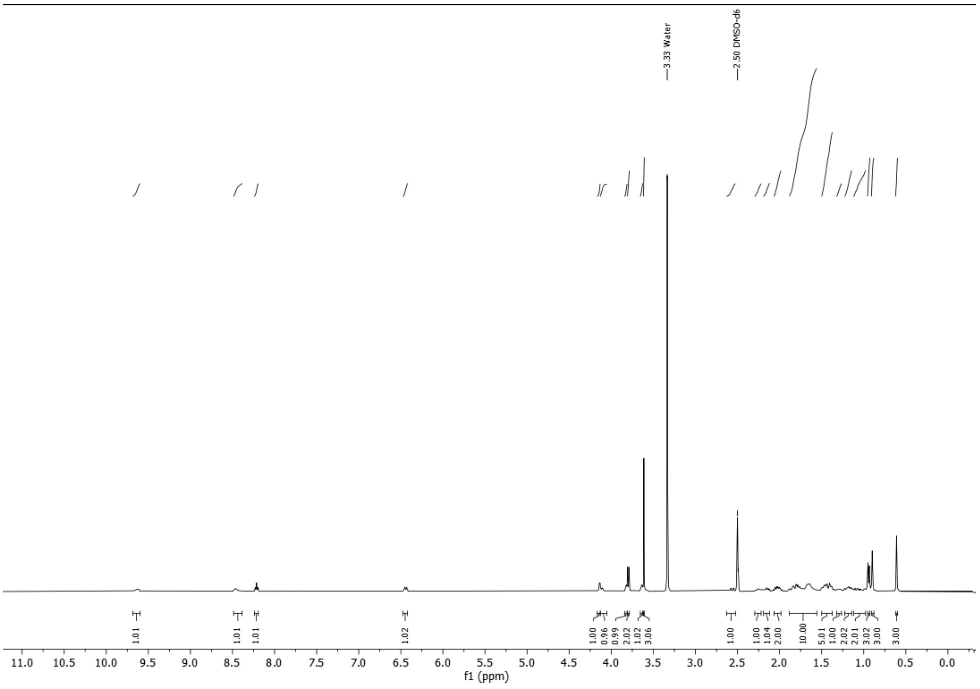

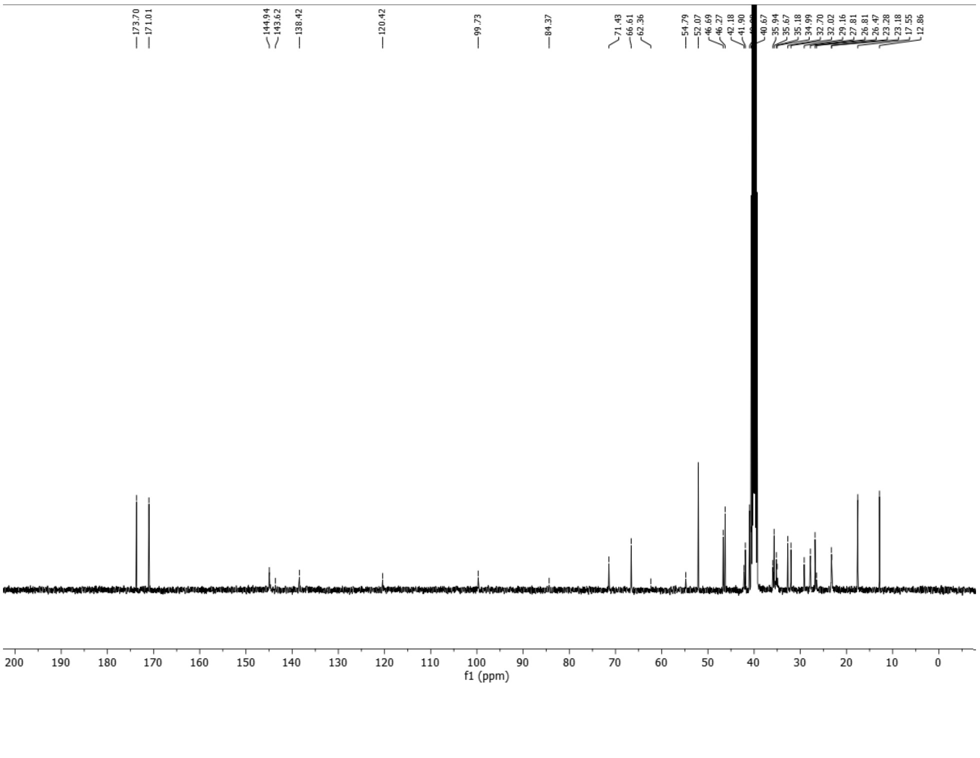

NBD G-BA

7α,12α-Dihydroxy-3β-[(7-nitro-2,1,3-benzoxadiazol-4-yl)amino]-5β-oxocholan-24-yl]glycine (16a)

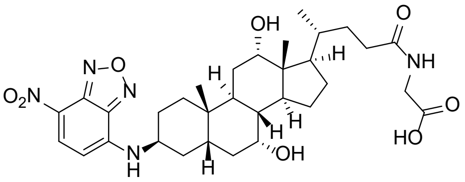

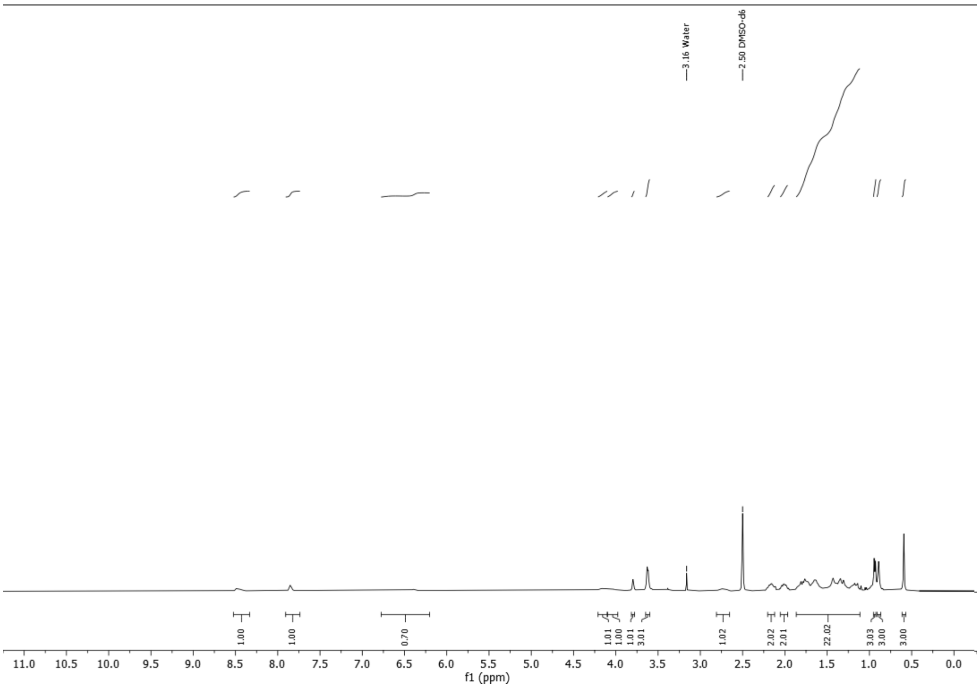

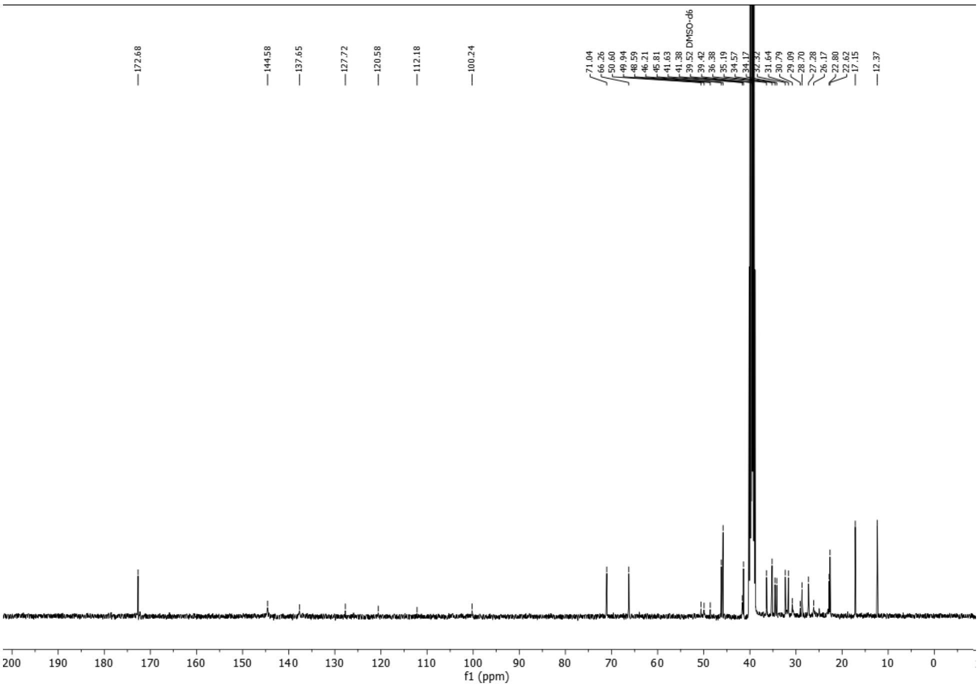

7α,12α-Dihydroxy-3α-[(7-nitro-2,1,3-benzoxadiazol-4-yl)amino]-5β-oxocholan-24-yl]glycine (16d)

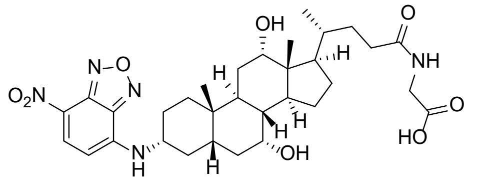

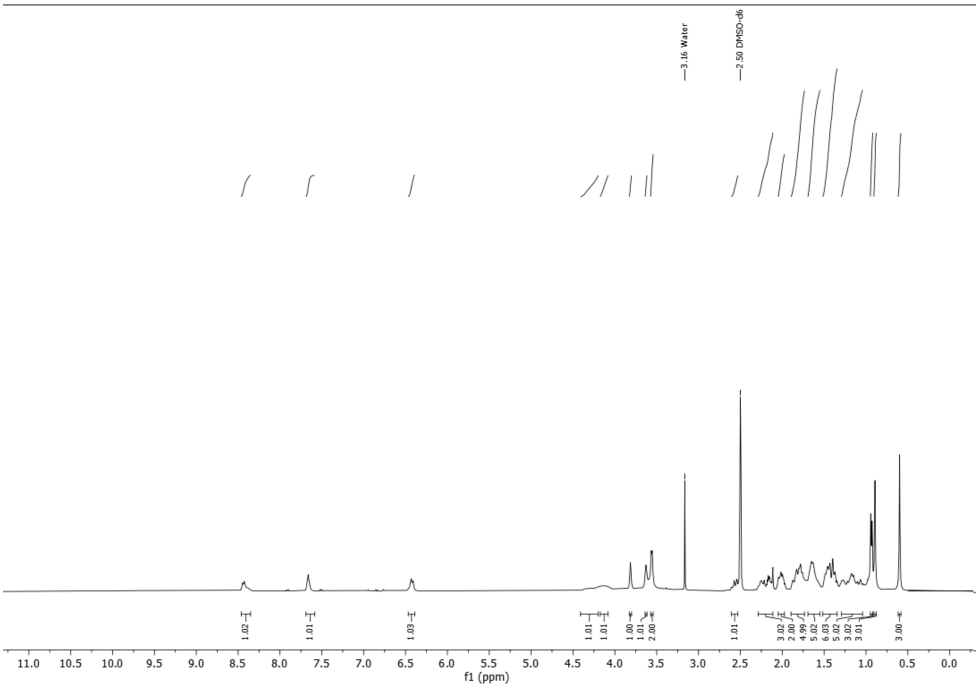

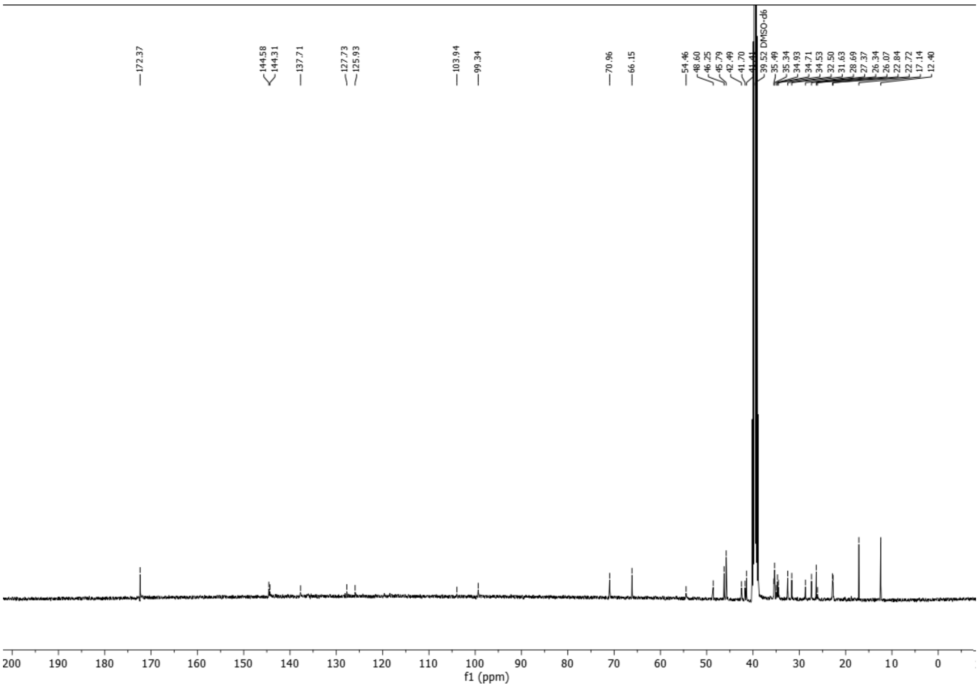

Methyl-7α,12α-dihydroxy-3β-[(methylsulfonyl)oxy]-5β-cholan-24-oate (7)

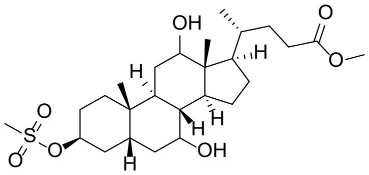

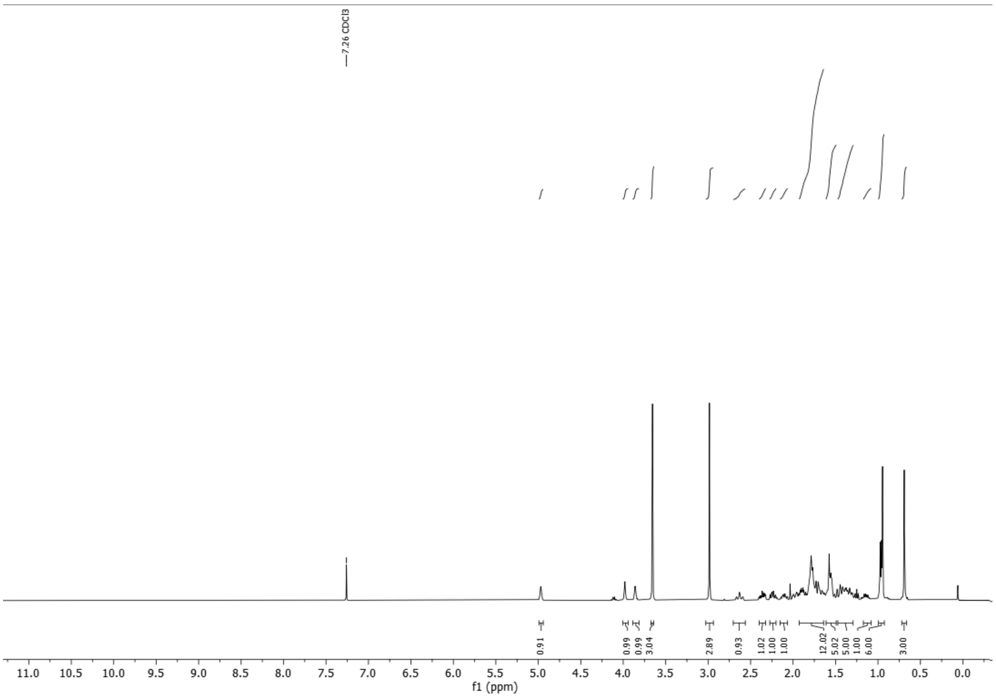

Methyl-7α,12α-dihydroxy-3β-[(trifluoroacetyl)oxy]-5β-cholan-24-oate (8)

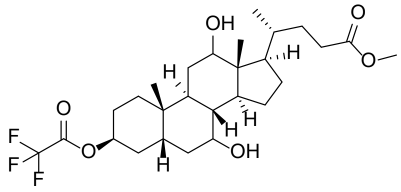

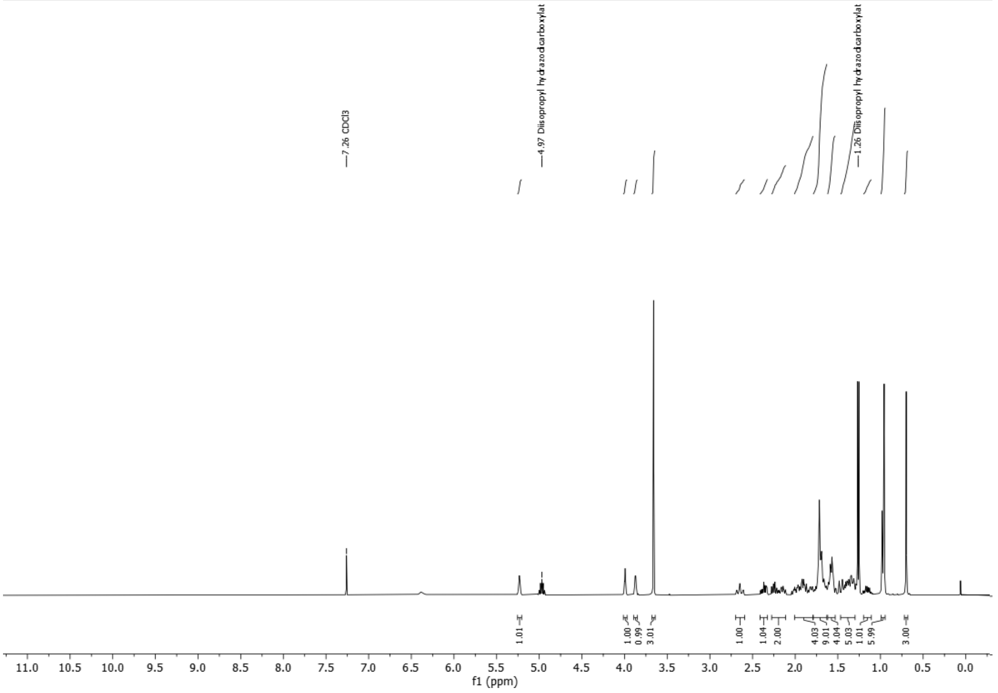

Methyl-7α,12α-dihydroxy-3β-(acetyloxy)-5β-cholan-24-oate

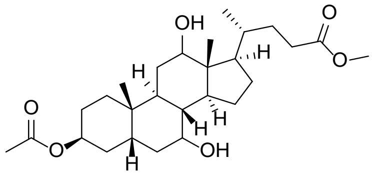

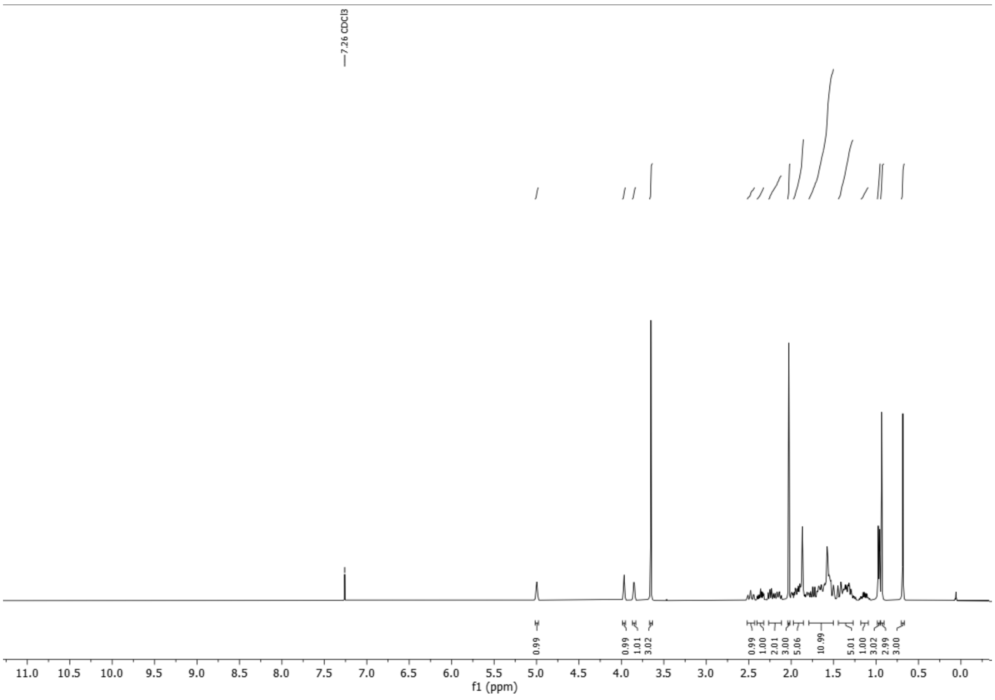

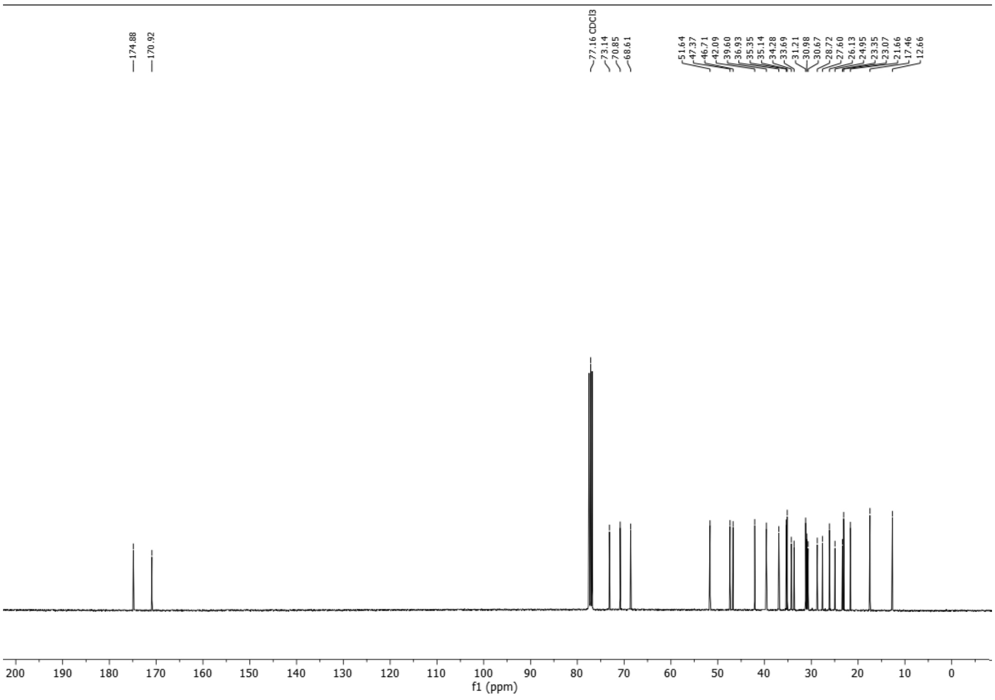

tert-Butyl-3α,7α,12α-trihydroxy-5β-cholan-24-oate

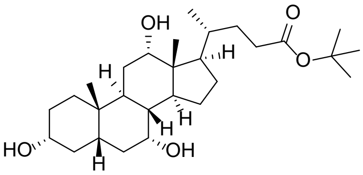

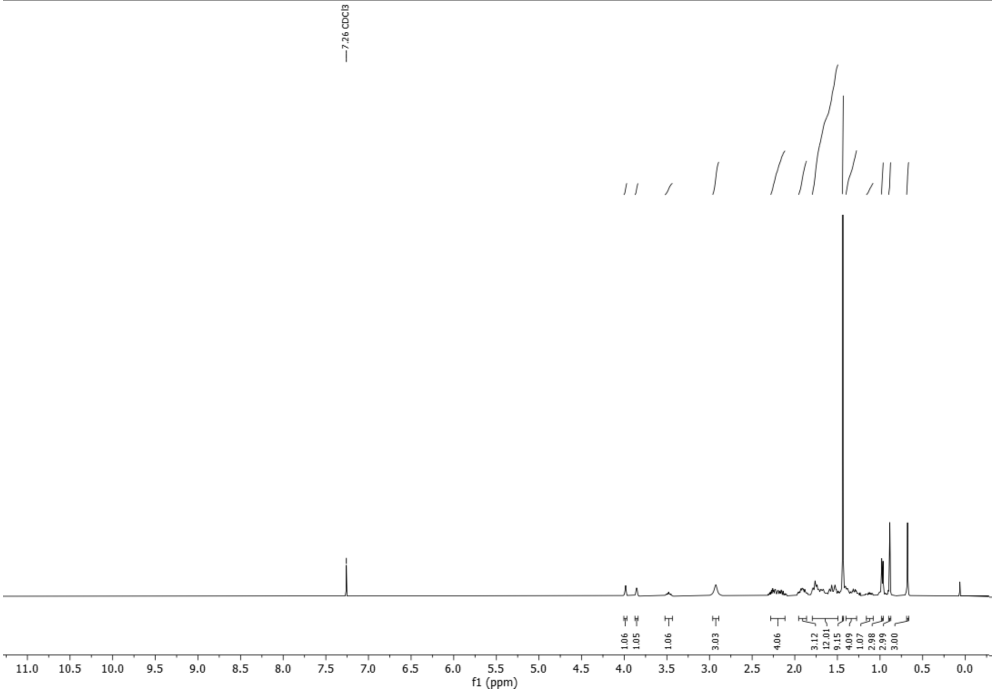

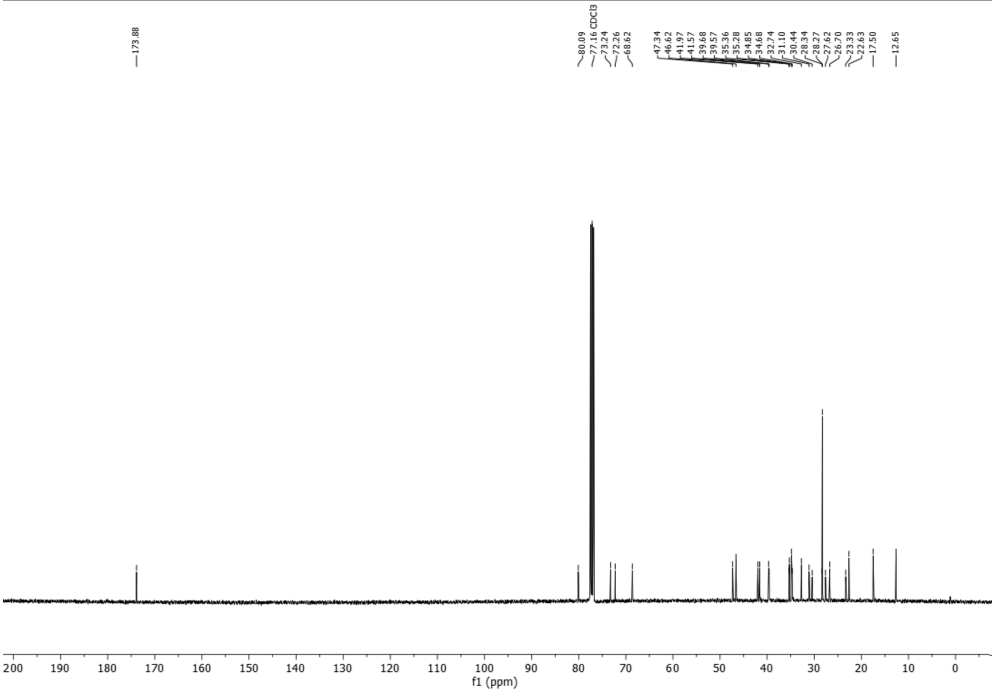

***tert*-Butyl-7α,12α-dihydroxy-3β-(acetyloxy)-5β-cholan-24-oate**

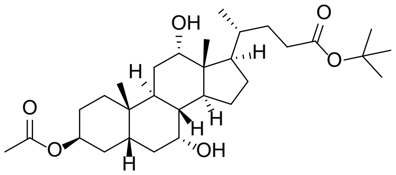

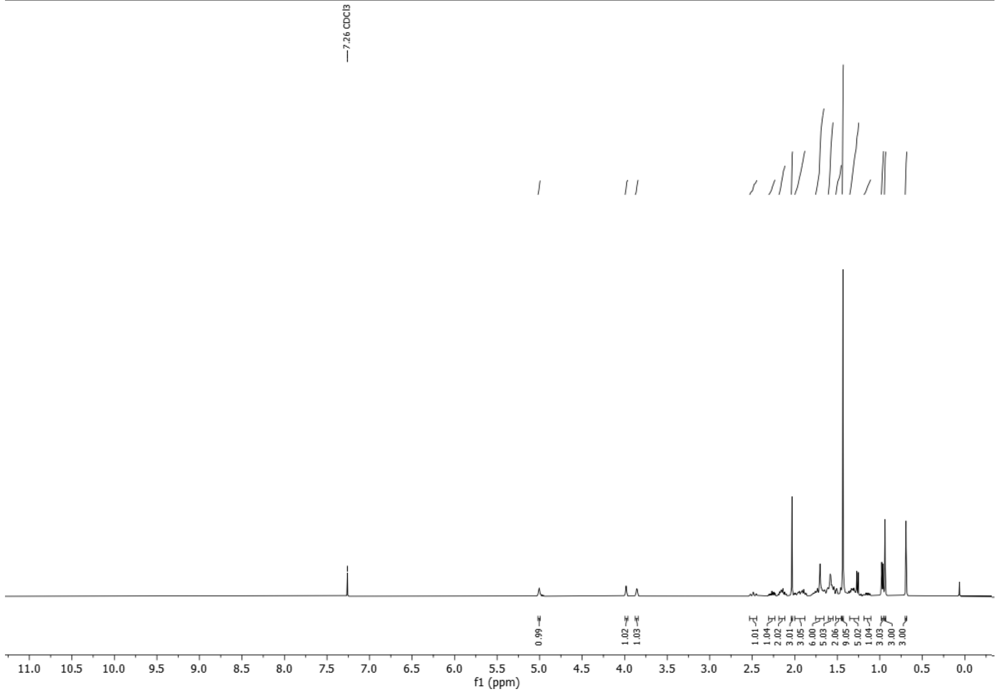

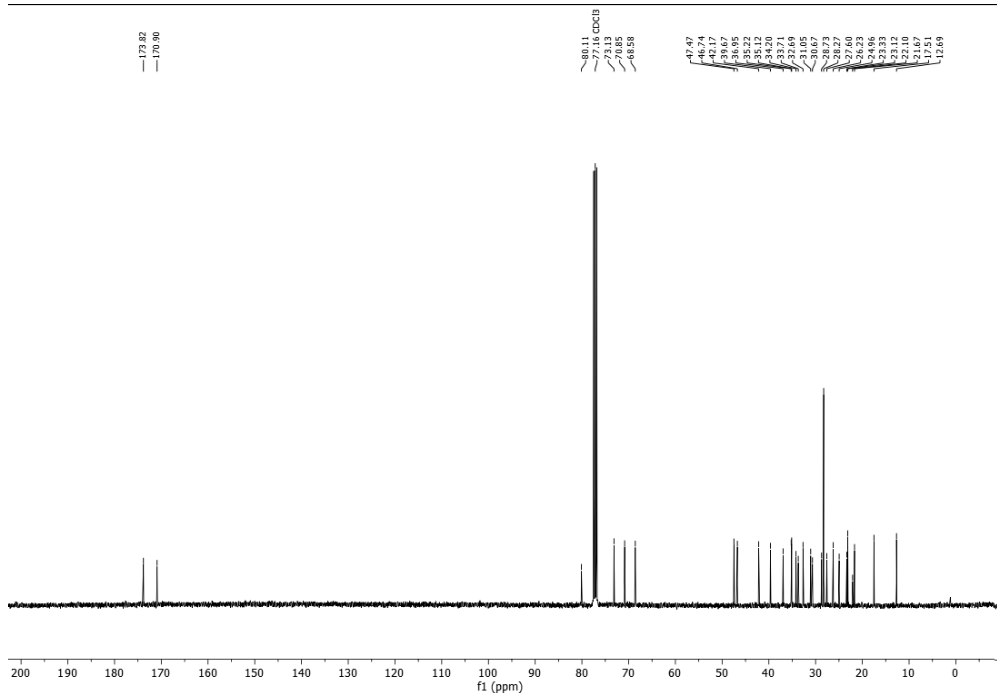

***tert*-Butyl-3β,7α,12α-trihydroxy-5β-cholan-24-oate**

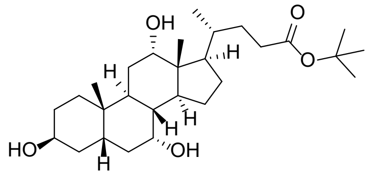

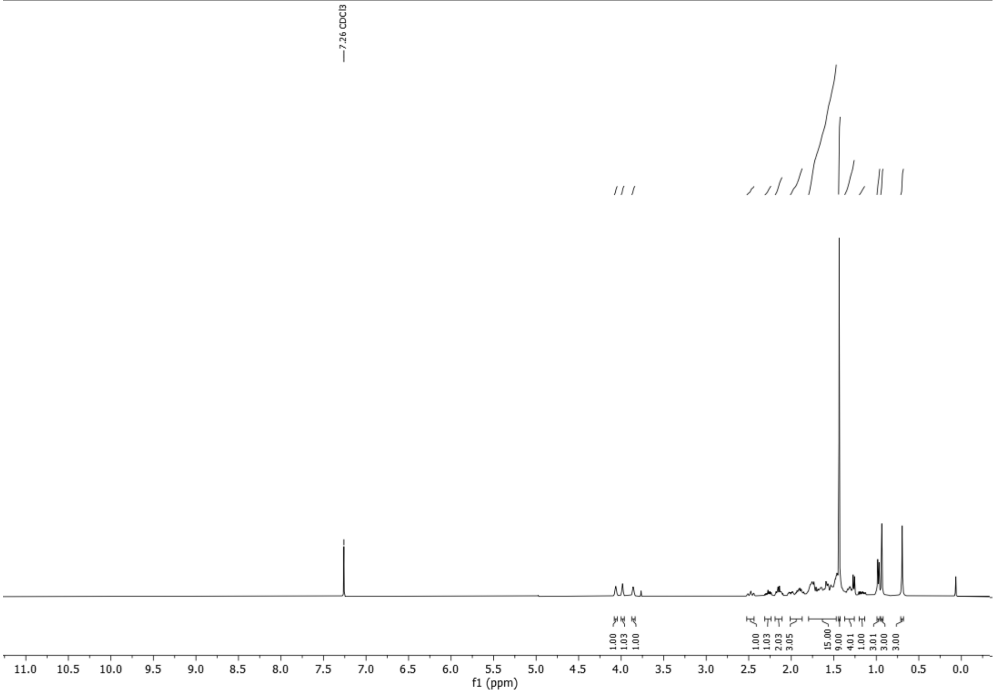

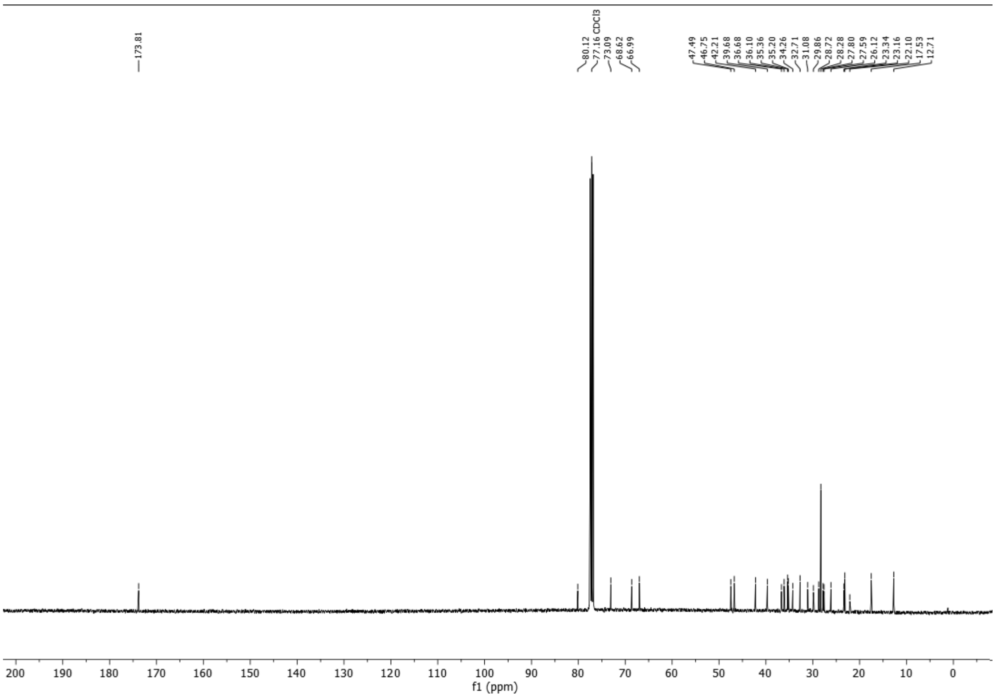

HPLC Chromatograms

7α,12α-Dihydroxy-3β-[(7-nitro-2,1,3-benzoxadiazol-4-yl)amino]-5β-cholan-24-oate (6a)

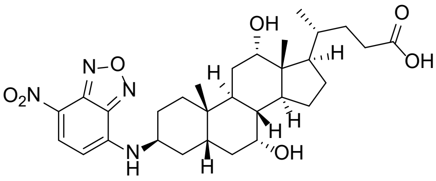

Method: Eurospher II C18H | 1 mL/min | 90 % MeOH, 10 % H_2O, 0,1 % AA | UV_VIS | 468 nm

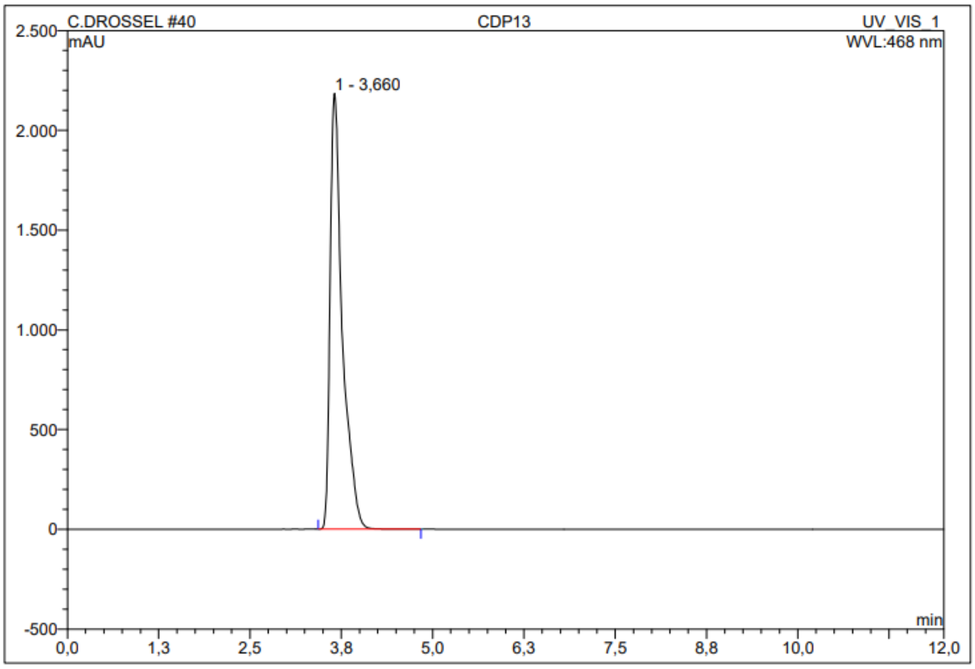

No.	Ret.Time min	Peak Name	Height mAU	Area mAU*min	Rel.Area %	Amount n.a.	Type
1	3,66	n.a.	2183,890	425,310	100,00	n.a.	BMB
Total:			2183,890	425,310	100,00	0,000	

7α,12α-Dihydroxy-3β-[(7-nitro-2,1,3-benzoxadiazol-4-yl)amino]-5β-oxocholan-24yl]amino]ethane sulfonic acid (14a)

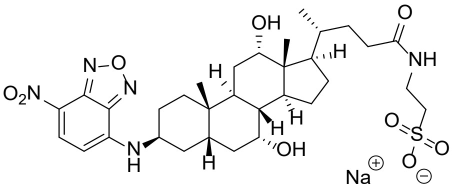

Method: Eurospher II C18H | 1 mL/min | 90 % MeOH, 10 % H_2O, 0,1 % AA | UV_VIS | 468 nm

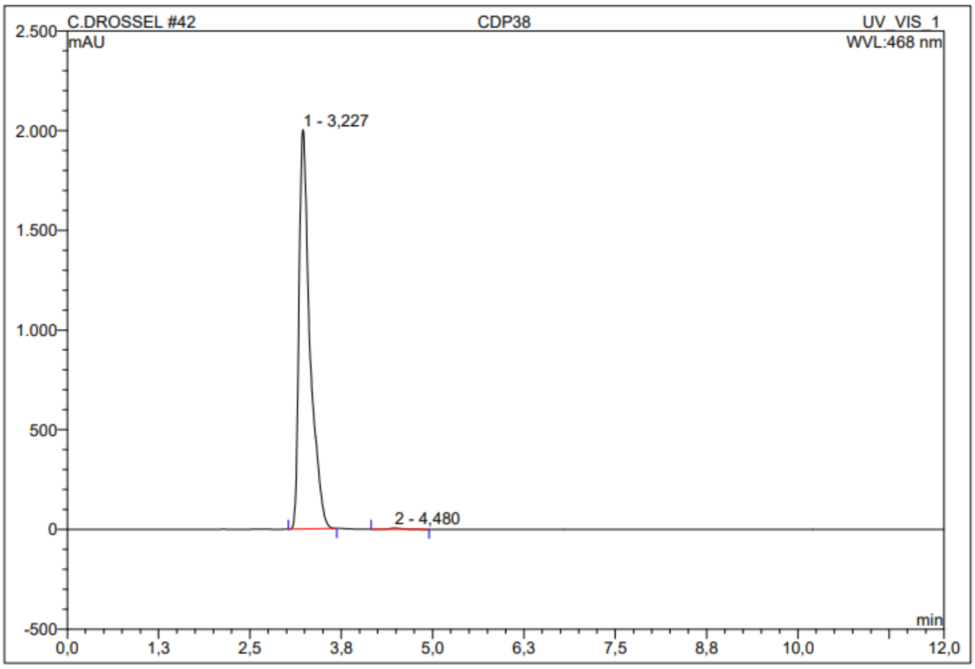

No.	Ret.Time min	Peak Name	Height mAU	Area mAU*min	Rel.Area %	Amount n.a.	Type
1	3,23	n.a.	2002,382	347,330	99,63	n.a.	BMB
2	4,48	n.a.	5,959	1,292	0,37	n.a.	BMB
Total:			2008,341	348,622	100,00	0,000	

7α,12α-Dihydroxy-3β-[(7-nitro-2,1,3-benzoxadiazol-4-yl)amino]-5β-oxocholan-24-yl]glycine (16a)

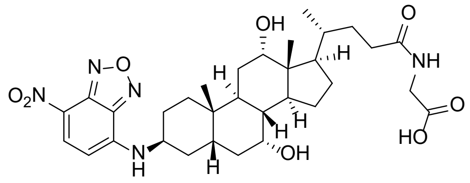

Method: Eurospher II C18H | 1 mL/min | 90 % MeOH, 10 % H_2O, 0,1 % AA | ELSD

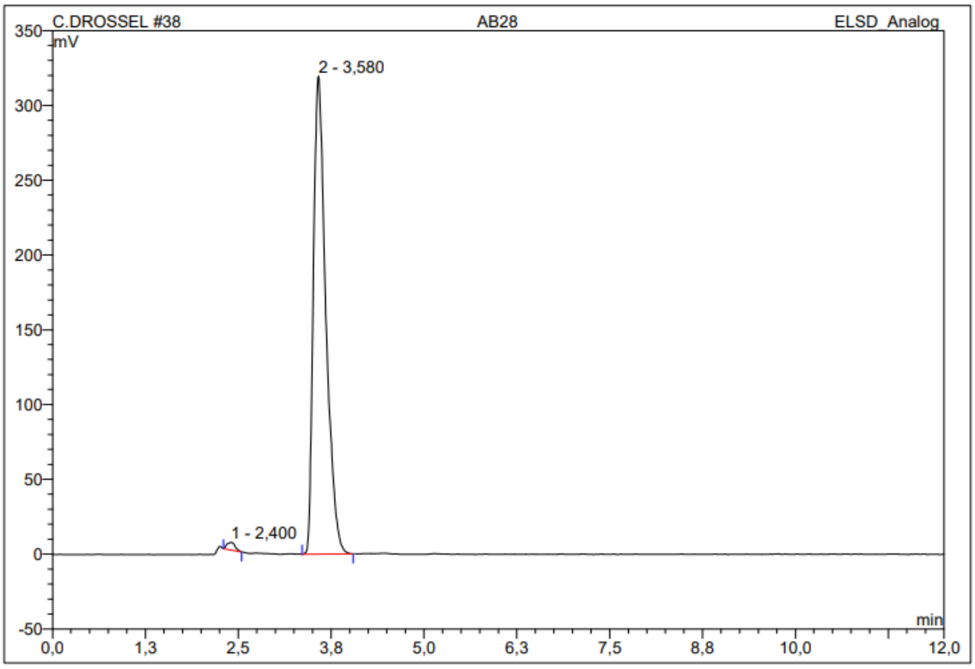

No.	Ret.Time min	Peak Name	Height mV	Area mV*min	Rel.Area %	Amount n.a.	Type
1	2,40	n.a.	5,181	0,654	1,04	n.a.	BMB
2	3,58	n.a.	319,518	61,965	98,96	n.a.	BMB
Total:			324,699	62,619	100,00	0,000	

7α,12α-Dihydroxy-3α-[(7-nitro-2,1,3-benzoxadiazol-4-yl)amino]-5β-cholan-24-oate (6d)

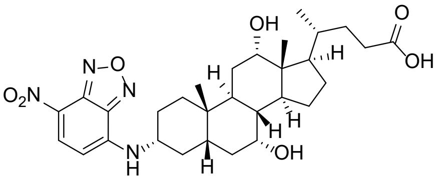

Method: Eurospher II C18H | 1 mL/min | 90 % MeOH, 10 % H_2O, 0,1 % AA | ELSD

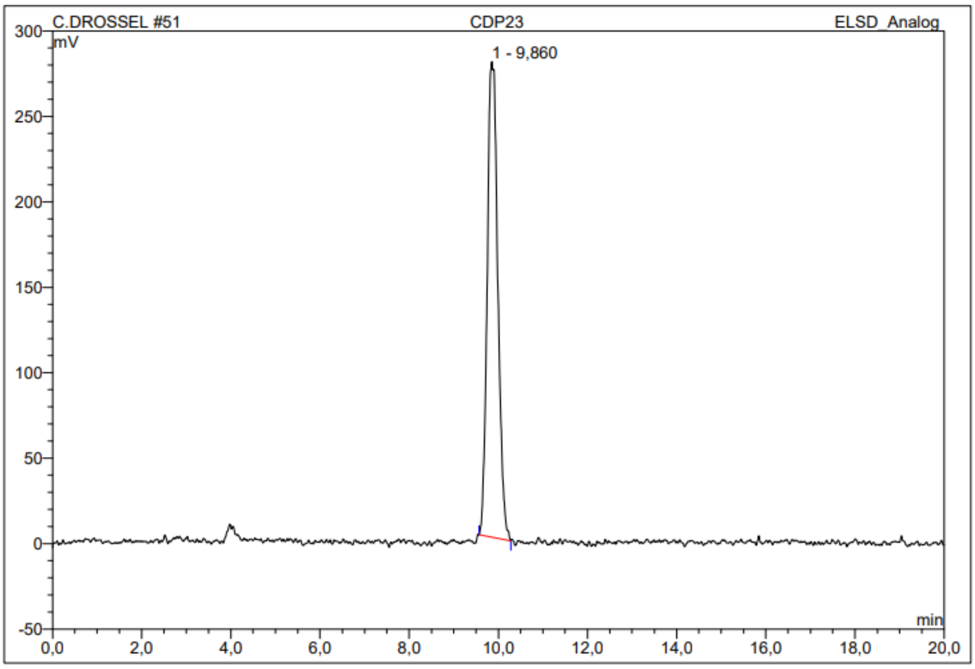

No.	Ret.Time min	Peak Name	Height mV	Area mV*min	Rel.Area %	Amount n.a.	Type
1	9,86	n.a.	278,307	76,485	100,00	n.a.	BMB
Total:			278,307	76,485	100,00	0,000	

7α,12α-Dihydroxy-3α-[(7-nitro-2,1,3-benzoxadiazol-4-yl)amino]-5β-oxocholan-24-yl]amino]ethane sulfonic acid (14d)

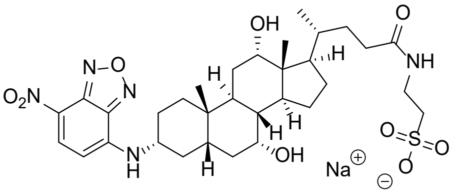

Method: Eurospher II C18H | 1 mL/min | 90 % MeOH, 10 % H_2O, 0,1 % AA | ELSD

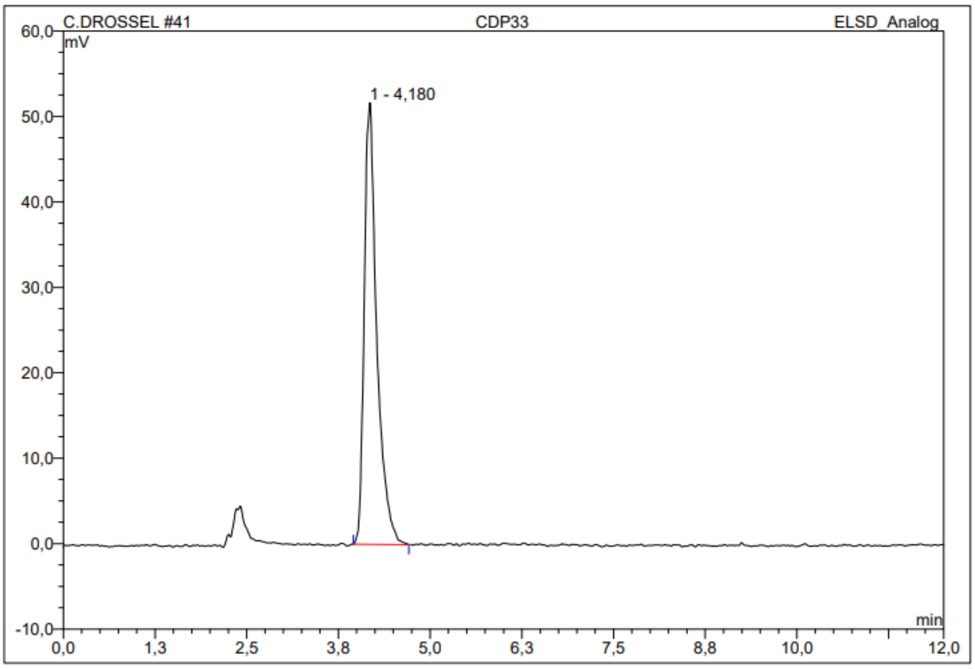

No.	Ret.Time min	Peak Name	Height mV	Area mV*min	Rel.Area %	Amount n.a.	Type
1	4,18	n.a.	51,695	10,281	100,00	n.a.	BMB
Total:			51,695	10,281	100,00	0,000	

7α,12α-Dihydroxy-3α-[(7-nitro-2,1,3-benzoxadiazol-4-yl)amino]-5β-oxocholan-24-yl]glycine (16d)

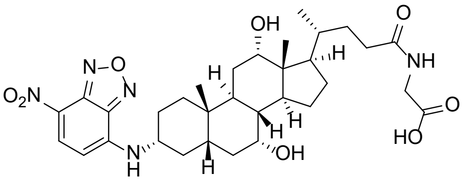

Method: Eurospher II C18H | 1 mL/min | 90 % MeOH, 10 % H_2O, 0,1 % AA | UV_VIS | 468 nm

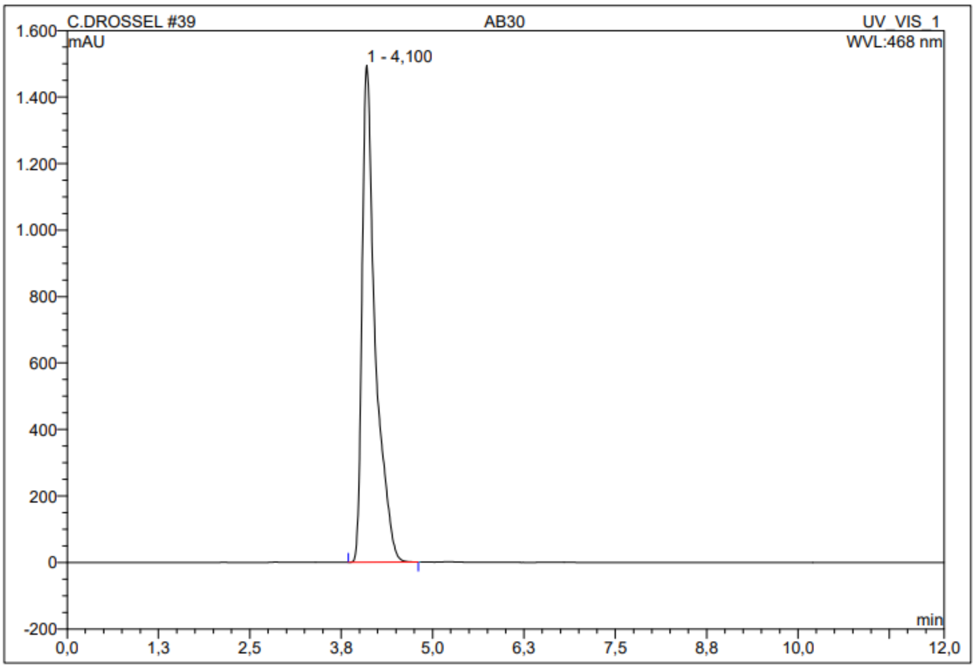

No.	Ret.Time min	Peak Name	Height mAU	Area mAU*min	Rel.Area %	Amount n.a.	Type
1	4,10	n.a.	1494,057	312,342	100,00	n.a.	BMB
Total:			1494,057	312,342	100,00	0,000	

7α-Hydroxy-3β-[(7-nitro-2,1,3-benzoxadiazol-4-yl)amino]-5β-cholan-24-oate (6b)

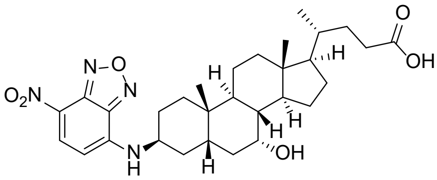

Method: Eurospher II C18H | 1 mL/min | 90 % MeOH, 10 % H_2O, 0,1 % AA | UV_VIS | 468 nm

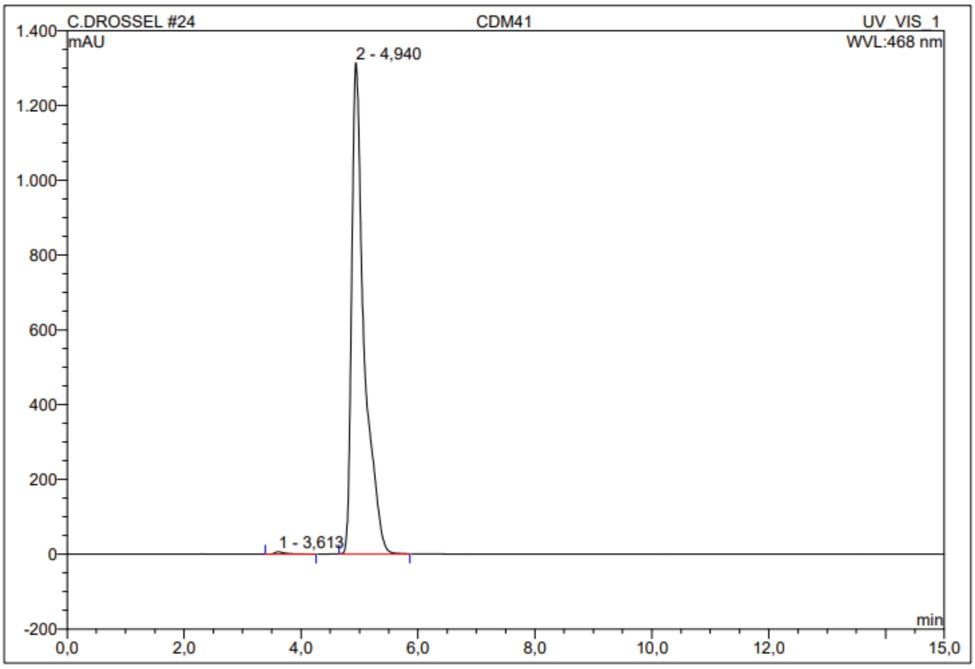

No.	Ret.Time min	Peak Name	Height mAU	Area mAU*min	Rel.Area %	Amount n.a.	Type
1	3,61	n.a.	6,259	1,299	0,40	n.a.	BMB
2	4,94	n.a.	1312,285	322,607	99,60	n.a.	BMB
Total:			1318,545	323,905	100,00	0,000	

7α-Hydroxy-3β-[(7-nitro-2,1,3-benzoxadiazol-4-yl)amino]-5β-oxocholan-24-yl]amino]ethane sulfonic acid (14b)

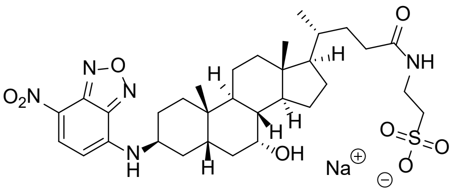

Method: Eurospher II C18H | 1 mL/min | 90 % MeOH, 10 % H_2O, 0,1 % AA | ELSD

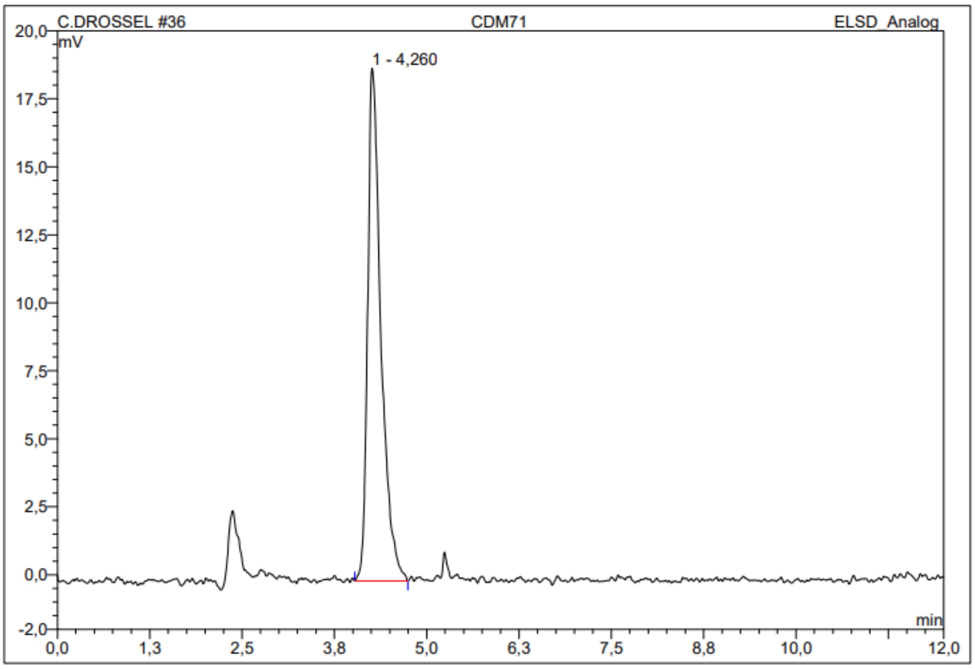

No.	Ret.Time min	Peak Name	Height mV	Area mV*min	Rel.Area %	Amount n.a.	Type
1	4,26	n.a.	18,847	3,931	100,00	n.a.	BMB
Total:			18,847	3,931	100,00	0,000	

12α-Hydroxy-3β-[(7-nitro-2,1,3-benzoxadiazol-4-yl)amino]-5β-cholan-24-oate (6c)

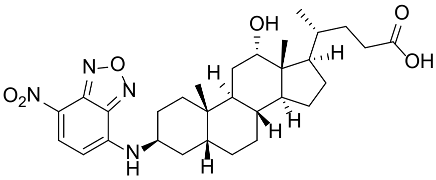

Method: Eurospher II C18H | 1 mL/min | 90 % MeOH, 10 % H_2O, 0,1 % AA | ELSD

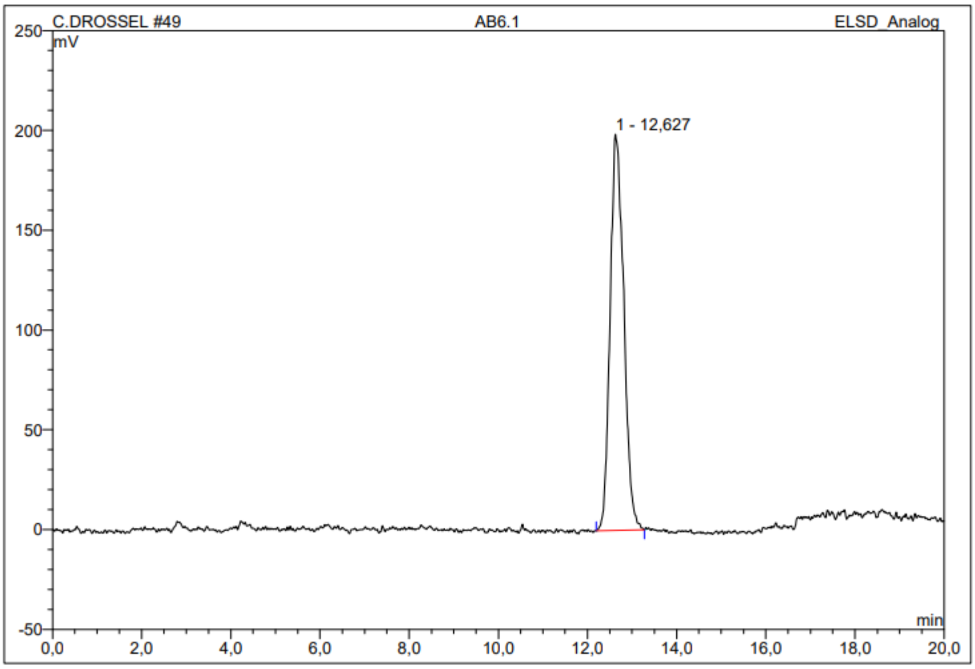

No.	Ret.Time min	Peak Name	Height mV	Area mV*min	Rel.Area %	Amount n.a.	Type
1	12,63	n.a.	198,617	70,268	100,00	n.a.	BMB
Total:			198,617	70,268	100,00	0,000	

Time-dependent Transport (Addition to Figure 6 and Figure 7)

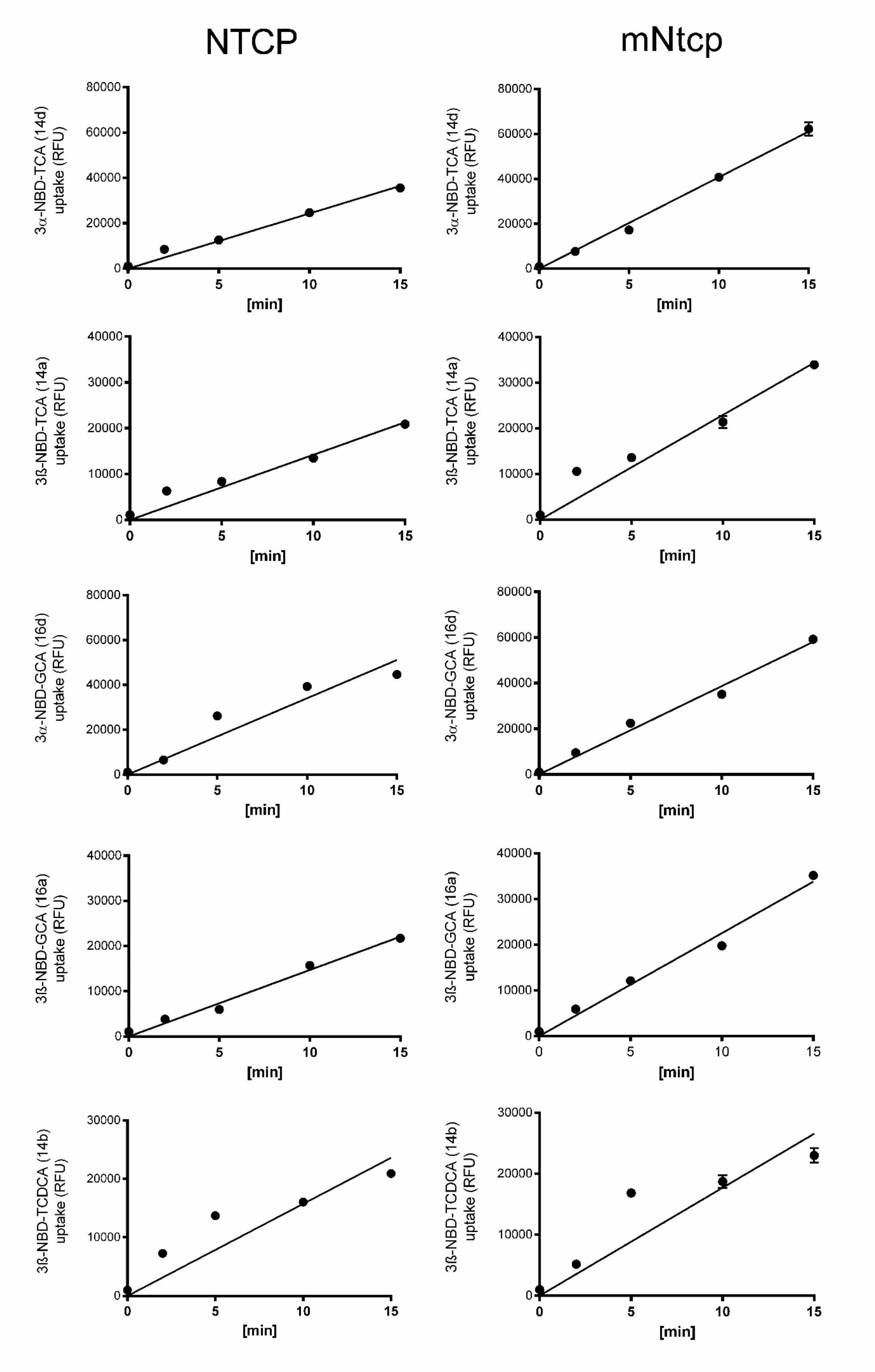

Time-dependent transport of conjugated 3-NBD-BA via NTCP and mNtcp. Substrate concentration: 50 µM.

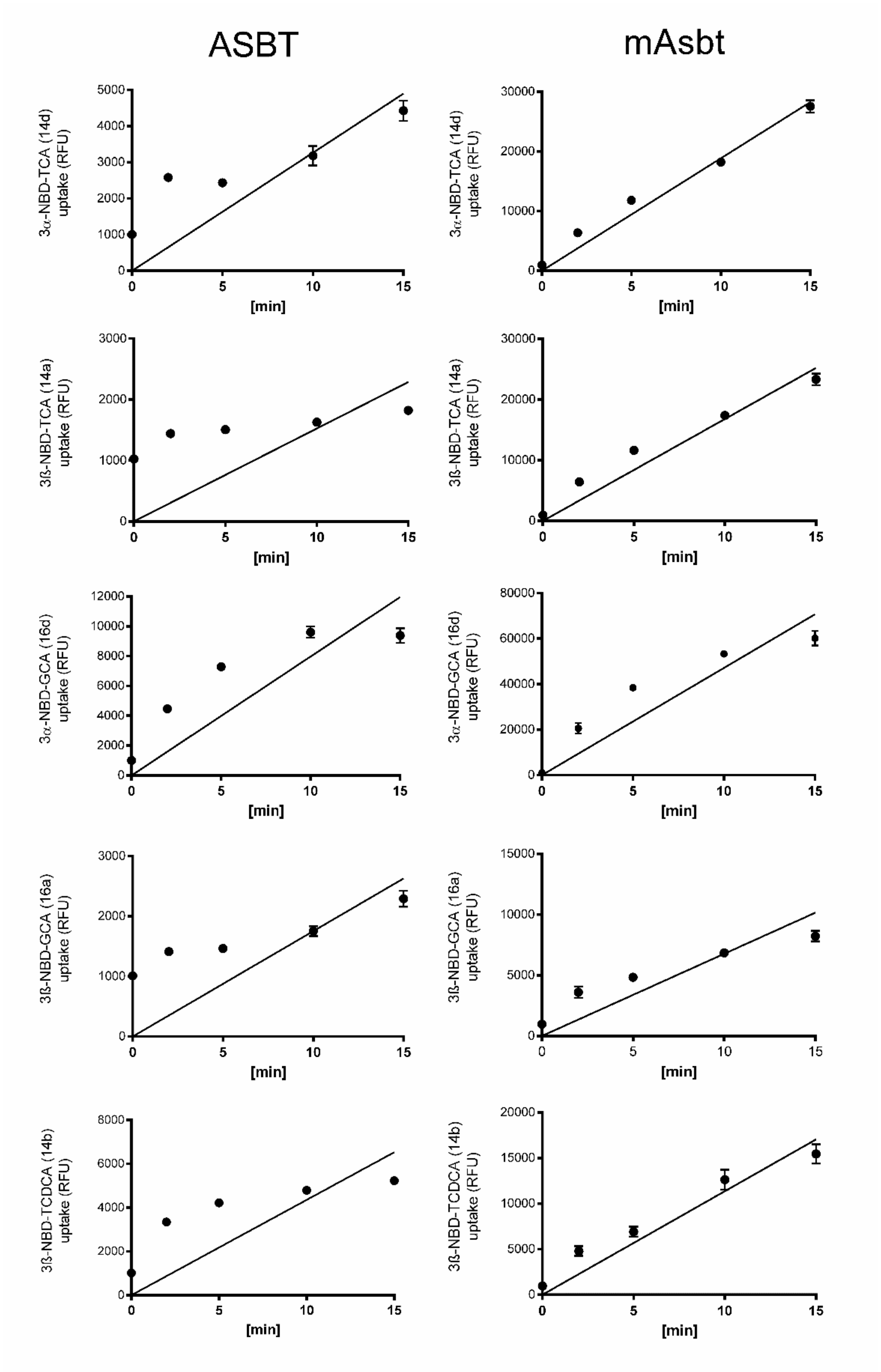

Time-dependent transport of conjugated 3-NBD-BA via ASBT and mAsbt. Substrate concentration: 50 µM.

95% Confidence Intervals (CI) of the K_m and V_{max} values (Addition to Table 1)

Mean K_m and V_{max} values are provided in **Table 1**.

	NTCP		mNtcp		ASBT		mAsbt	
	K_m [µM]	V_{max} [RFU/10 min]	K_m [µM]	V_{max} [RFU/10 min]	K_m [µM]	V_{max} [RFU/10 min]	K_m [µM]	V_{max} [RFU/10 min]
3α-NBD-TCA **(14d)**	7.1 - 32.8	20,721 – 30,762	1.1 - 18.0	43,461 – 61,716	-	-	4.9 - 17.2	28744 - 36390
3ß-NBD-TCA **(14a)**	9.9 - 49.1	19,224 – 30,201	2.8 - 38.3	21,002 – 34,122	-	-	6.9 - 33.1	29166 - 41213
3α-NBD-GCA **(16d)**	19.4 - 54.3	42,363 – 59,500	0 - 11.5	33,928 – 47,396	2.0 - 27.0	2,977 – 4,913	7.8 - 21.2	83761 - 104464
3ß-NBD-GCA **(16a)**	31.2 - 55.0	26,158 – 32,146	11.4 - 23.1	24,390 – 29,082	-	-	1.6 - 5.4	6773 - 7578
3ß-NBD-TCDCA **(14b)**	7.8 - 26.4	14,604 – 20,138	47.0 - 69.9	45,439 – 52,201	-	-	16.3 - 26.0	19223 - 21846
